# Citrullination Was Introduced into Animals by Horizontal Gene Transfer from Cyanobacteria

**DOI:** 10.1093/molbev/msab317

**Published:** 2021-11-03

**Authors:** Thomas F M Cummings, Kevin Gori, Luis Sanchez-Pulido, Gavriil Gavriilidis, David Moi, Abigail R Wilson, Elizabeth Murchison, Christophe Dessimoz, Chris P Ponting, Maria A Christophorou

**Affiliations:** 1 MRC Human Genetics Unit, Institute of Genetics and Molecular Medicine, University of Edinburgh, Western General Hospital, Edinburgh, United Kingdom; 2 Transmissible Cancer Group, Department of Veterinary Medicine, Cambridge, United Kingdom; 3 Department of Computational Biology, University of Lausanne, Lausanne, Switzerland; 4 Center for Integrative Genomics, University of Lausanne, Lausanne, Switzerland; 5 Swiss Institute of Bioinformatics, Lausanne, Switzerland; 6 Department of Genetics Evolution and Environment, University College London, London, United Kingdom; 7 Department of Computer Science, University College London, London, United Kingdom; 8 Epigenetics Department, The Babraham Institute, Cambridge, United Kingdom

**Keywords:** citrullination, posttranslational modification, horizontal gene transfer, enzyme

## Abstract

Protein posttranslational modifications add great sophistication to biological systems. Citrullination, a key regulatory mechanism in human physiology and pathophysiology, is enigmatic from an evolutionary perspective. Although the citrullinating enzymes peptidylarginine deiminases (PADIs) are ubiquitous across vertebrates, they are absent from yeast, worms, and flies. Based on this distribution *PADIs* were proposed to have been horizontally transferred, but this has been contested. Here, we map the evolutionary trajectory of *PADIs* into the animal lineage. We present strong phylogenetic support for a clade encompassing animal and cyanobacterial *PADIs* that excludes fungal and other bacterial homologs. The animal and cyanobacterial PADI proteins share functionally relevant primary and tertiary synapomorphic sequences that are distinct from a second PADI type present in fungi and actinobacteria. Molecular clock calculations and sequence divergence analyses using the fossil record estimate the last common ancestor of the cyanobacterial and animal *PADIs* to be less than 1 billion years old. Additionally, under an assumption of vertical descent, PADI sequence change during this evolutionary time frame is anachronistically low, even when compared with products of likely endosymbiont gene transfer, mitochondrial proteins, and some of the most highly conserved sequences in life. The consilience of evidence indicates that *PADIs* were introduced from cyanobacteria into animals by horizontal gene transfer (HGT). The ancestral cyanobacterial PADI is enzymatically active and can citrullinate eukaryotic proteins, suggesting that the *PADI* HGT event introduced a new catalytic capability into the regulatory repertoire of animals. This study reveals the unusual evolution of a pleiotropic protein modification.

## Introduction

Posttranslational modifications (PTMs) allow for temporal and spatial control of protein function in response to cellular and environmental signals and comprise an integral part of cellular and organismal life. The development of ever more sensitive and quantitative analytical methods has made possible the identification of PTMs within cells and has enhanced our understanding of the molecular and cellular functions they regulate. This has led to renewed interest in studying previously known, as well as newly identified modifications. Although PTMs have been classically studied in eukaryotic organisms, an increasing number of them are also found in bacteria (Koonin 2010). Some PTMs, such as phosphorylation, acetylation, and glycosylation are ubiquitous across all domains of life suggesting that the enzymes that catalyze them existed in the Last Universal Common Ancestor (LUCA) (Beltrao et al. 2013). In other cases, such as protein ubiquitylation, this is less clear. Although ubiquitin itself is absent from eubacteria and archaea, other ubiquitin-like domains have been identified and shown to be added and removed from proteins in a similar manner in bacteria (Iyer et al. 2008; Pearce et al. 2008; Hochstrasser 2009; Koonin 2010; Macek et al. 2019).

Citrullination is the posttranslational conversion of a protein arginine residue to the noncoded amino acid citrulline and is catalyzed by peptidylarginine deiminase (PADI) enzymes in a calcium-dependent manner (Sugawara et al. 1982; Wang and Wang 2013). Although citrullination involves a small mass change of only 0.98 Da, the removal of a positive charge from the arginine side chain can lead to profound biochemical changes and is known to alter protein structure, subcellular localization and affinity to other proteins and nucleic acids (Tanikawa et al. 2009, 2018; Guo and Fast 2011; Stadler et al. 2013; Christophorou et al. 2014; Snijders et al. 2015; Sharma et al. 2019). Via these alterations PADIs regulate fundamental physiological processes such as gene expression, chromatin compaction, and the innate immune response to bacterial infection (Wang et al. 2009; Wang and Wang 2013; Christophorou et al. 2014). Notably, deregulation of PADIs is strongly implicated in the etiology of a host of pathologies including autoimmunity (rheumatoid arthritis, ulcerative colitis, psoriasis, and type I diabetes), neurodegeneration (multiple sclerosis, Alzheimer’s, and prion diseases), and metastatic cancer (Suzuki et al. 2003; Musse et al. 2008; Zhang et al. 2011; Wang and Wang 2013; Yuzhalin et al. 2018), whereas loss of PADI activity compromises neurodevelopment, fertility, and embryo development (Christophorou et al. 2014; Xu et al. 2016; Falcão et al. 2019).

In an evolutionary context, *PADIs* are puzzling. Orthologs of the human *PADIs* are ubiquitous in bony fish, birds, reptiles, amphibians, and mammals, but are unexpectedly missing from many eukaryotes including plants, yeast, worms, and insects. The *PADI* gene is widely thought to have appeared first in the last common ancestor of teleosteans and mammals (Balandraud et al. 2005; Wang and Wang 2013; Nicholas and Bhattacharya 2014), with duplications in subsequent lineages resulting in five mammalian paralogs. Therefore, citrullination seemingly defies the perception that PTMs are of ancient origin (Beltrao et al. 2013).

Mammalian PADIs consist of three structural domains, the N-terminal (PAD_N, Pfam annotation: PF08526), middle (PAD_M, Pfam annotation: PF08527), and catalytic C-terminal domain (PAD_C, Pfam annotation: PF03068). Although PADI proteins are widely considered to be specific to vertebrates, their crystal structures (Arita et al. 2004; Slade et al. 2015) hint at a possibly more ancient origin as they reveal that the catalytic (PAD_C) domain adopts the same pentein fold as a variety of other widely distributed proteins that otherwise show little similarity in terms of amino acid conservation (Shirai et al. 2001; Linsky and Fast 2010) (supplementary fig. S1, Supplementary Material online). The pentein-fold containing group of proteins comprises a broad family of guanidino-group (the functional group of the side chain of arginine and agmatine) modifying enzymes that possess hydrolase, dihydrolase, and amidinotransferase catalytic activity, sharing a catalytic core of a Cys, His, and two polar guanidine-binding residues—Asp or Glu (Linsky and Fast 2010). Two such proteins with citrullinating activity are known among some bacteria and eukaryotes: pPAD, an extended agmatine deiminase found in *Porphyromonas gingivalis* and giardiaADI, an extended form of the free *L*-arginine deiminase gADI, found in the human parasite *Giardia lamblia* (Touz et al. 2008; Goulas et al. 2015). These enzymes contain a distant PAD_C domain but lack PAD_N and PAD_M domains, are highly divergent in sequence, and have different substrate specificities. In addition, mammalian genomes encode two distant homologs of the PAD_C domain: N(G),N(G)-dimethylarginine dimethylaminohydrolase (DDAH) and glycine amidinotransferase (AGAT) (Linsky and Fast 2010). Both DDAH and AGAT are divergent in sequence, also lack PAD_N and PAD_M domains and do not appear to catalyze citrullination. The presence of this ancient fold and catalytic triad within PAD_C suggests that it may have been present early in cellular life, but the evolutionary provenance of the animal PADI enzymes has remained unclear.

A 2015 study by Crisp et al., identified possible *PADI* homologs in some bacterial species. Based on the finding that a possible homolog could be identified in prokaryotes but not in multiple *Drosophila* and *Caenorhabditis* species, the authors included *PADIs* among a list of 145 genes proposed to have been transferred into the genome of a vertebrate ancestor of extant mammals by horizontal gene transfer (HGT, also known as lateral gene transfer) (Crisp et al. 2015). HGT is the nonheritable transmission of genetic material from one organism to another, often via a virus or mobile genetic element and involving endosymbiotic or commensal relationships between donor and recipient (Boto 2014; Soucy et al. 2015). HGT is widespread among prokaryotes and is recognized as a mechanism that shapes the evolution and adaptive potential of bacteria, for example, in the acquisition of antibiotic resistance (Ochman et al. 2000; Koonin et al. 2001). Although many cases of horizontal transfer have been reported between bacteria and unicellular eukaryotes, fewer bacteria-to-animal HGT events have been reported to date (Keeling and Palmer 2008; Dunning Hotopp 2011; Boto 2014). The majority of cases involve transfer into an invertebrate host, such as an insect or worm (Gladyshev et al. 2008; Moran and Jarvik 2010; Chou et al. 2015; Lacroix and Citovsky 2016; Dunning Hotopp 2018). Moreover, it has been proposed that HGT into animals with specialized germline cells is very rare (Jensen et al. 2016). These few accounts of bacteria-to-animal HGT have been the topic of intense debate (Stanhope et al. 2001; Crisp et al. 2015; Martin 2017; Salzberg 2017; Husnik and McCutcheon 2018; Leger et al. 2018). The genome-wide approach employed by Crisp et al. to search for possible HGT events in vertebrates was disputed by Salzberg, and 45 of the highest confidence candidates were reanalyzed and rebutted on a case-by-case basis. In the instance of the *PADI* gene, this reanalysis showed that a *PADI* can also be identified in *Priapulus caudatus* (a marine worm) and therefore that the lack of *PADI* in at least *Drosophila* spp. must be explained by gene loss (Salzberg 2017). Salzberg additionally recalculated the HGT index for many of the possible HGT candidates, including the *PADIs*, in light of additional sequences that can be identified showing that they no longer pass the original parametric criterion for HGT proposed by Crisp et al. (Salzberg 2017). Individual claims of HGT should be considered carefully and tested against the alternative hypothesis of widespread independent gene losses (Salzberg 2017). In light of the absence of *PADI* homologs in most invertebrate animals, *PADI* evolution requires detailed consideration.

## Results

### Comprehensive Identification of PADI Homologs

In order to understand the distribution and evolution of citrullination, we sought to identify all *PADI* homologs from across life. We started by collecting orthologous *PADIs* using the EggNOG database, employing an unsupervised clustering algorithm of all proteins contained in 2031 genomes across cellular life (Huerta-Cepas et al. 2016). To expand on this list, we used HMMER searches to identify all sequences in current sequence databases that contain a PAD_C domain, as defined by having significant sequence similarity (E-value <1×10^−3^), and assessed these for the presence of critical substrate-binding and calcium-binding residues annotated to human PADIs (Slade et al. 2015). This was supplemented by additional iterative jackhmmer searches as well as TBlastN and Position-Specific Iterated BLAST (PSI-BLAST) searches of genomic databases.

The taxonomic distribution of *PADIs* and proportion of species that harbor a PADI ortholog are presented in table 1. PADIs are not ubiquitous across the metazoa but are present across major branches of vertebrates, including jawless fish, sharks and rays, bony fish, amphibians, reptiles, birds, and mammals. Out of all species whose genomes have been sequenced to date, the earliest diverging invertebrate animals with a *PADI* gene are *P. caudatus* (an ecdysozoan), *Saccoglossus kowalevskii* (a hemichordate), and *Branchiostoma belcheri* (a cephalochordate). In addition, we identified a number of PADI sequences with conservation of substrate and calcium-binding residues in bacteria and fungi. *PADIs* are also not ubiquitous across bacteria (found in fewer than 1% of bacterial species), and are most prevalent within cyanobacteria (found in 11% of cyanobacteria). No eukaryotes diverging before opisthokonta have a detectable *PADI* homolog. Our searches also returned two outliers, one in archaea and one in viruses. However, upon closer inspection, both hits were determined to be due to misattribution (supplementary figs. S2 and S3, Supplementary Material online; see also Materials and Methods) and were therefore not included in further analyses. This taxonomic distribution could suggest an evolutionary model in which *PADI* genes were lost independently in many separate lineages. In this scenario, gene loss occurred in all early branching lineages leading to at least 306 nonopisthokont eukaryotes and in other lineages, for example, those leading to *Drosophila* and *Caenorhabditis*.

**Table 1.** The Number and Proportion of Species Harboring a Putative PADI Ortholog.

Group	NCBI Taxonomy ID	Unique Species with a PADI	Species with Proteomes in UniprotKB	Percentage of Species with a PADI
Bacteria	2	295	38,842	0.76
Cyanobacteria	1,117	56	506	11.07
Actinobacteria	201,174	136	4,870	2.79
Proteobacteria	1,223	69	16,196	0.43
Eukaryotes	2,759	406	2,241	18.12
Animals (Metazoa)	33,208	229	612	37.42
Insects	50,557	0	142	0.00
Worms (Annelida)	6,340	0	2	0.00
Fungi	4,751	177	1,098	16.12
Yeast (Ascomycota)	4,890	176	760	23.16
Yeast (Saccharomyces)	4,930	0	13	0.00
Plants (Viridiplantae)	33,090	0	244	0.00
Opisthokonta (metazoa and fungi)	33,208 and 4,751	406	1,710	23.74
Pre-opisthokonta (eukarya, not metazoa or fungi)	2,759 and NOT (33,208|4,751)	0	531	0.00
Archaea	2,157	1	2,107	0.05
Viruses	10,239	1	99,210	0.001

Note.—HMM searches (https://www.ebi.ac.uk/Tools/hmmer, last accessed June 7, 2020) for similarity to the vertebrate PAD_C domain from human PADI2, were carried out using HmmerWeb version 2.41.1 against the UniProtKB (v.2019_09) database. Unique species with significant sequence similarity (E-value <1×10^−3^) are presented. Proportions are given relative to the total number of species in within UniProtKB, for each group.

To explore the relationship of PADIs to other distantly related sequences, we aligned fungal, bacterial, and animal PADIs with sequences possessing significant HMMER similarity to pPAD and gADI and conducted phylogenetic analysis under a time-reversible model (supplementary fig. S4, Supplementary Material online). Bacterial, fungal, and animal PADIs form a single outgroup that excludes both pPAD and gADI enzyme types, showing that each of the three types of protein is phyletically distinct. The pPAD and gADI type proteins can therefore be excluded from further consideration of the evolutionary origin of animal PADIs.

### A Strongly Supported Clade Contains Cyanobacterial and Animal but Not Fungal PADIs

Firstly, we used HMMER to obtain all PADI sequences in the UniProtKB rp55 database and performed phylogenetic analysis using MrBayes and IQTree, recovering a clade of animal and bacterial PADIs distinct from fungal and other bacterial PADIs (supplementary fig. S5*a* and *b*, Supplementary Material online). We then repeated the phylogenetic analyses on a subset of 150 sequences, ensuring the length of the alignment of PADI sequences (495 columns) was at least three times the number of taxa considered in the tree, to limit “rough likelihood surface” issues that may arise with data sets of relatively few sites and many taxa (Stamatakis et al. 2020) (supplementary fig. S5*c*, Supplementary Material online). To avoid possible biases in subsampling, we took all bacterial PADI sequences contained within the Pathosystems Resource Integration Center (PATRIC) database for analysis (82 sequences). We then included 35 fungal sequences that cover the broadest span in HMMER sequence similarity to the human sequence (E-values between 5×10^−26^ and 1.4×10^−46^). Finally, we subsampled metazoan sequences to maximize lineage representation in species maintaining a PADI (the five paralogs in *Homo sapiens*, *Pongo abelii*, and in *Mus musculus*, the three paralogs found in *Gallus*, *Chelonia mydas*, and *Alligator mississipiensis*, and the single paralog found in *Xenopus laevis*, *Takifugu rubripes*, *Tetraodon nigroviridis*, *Astyanax mexicanus*, *Danio rerio*, *Oncorhynchus mykiss*, *Callorhinchus milii*, *B. floridae*, and *P. caudatus*). Amino acid sequences were used as this enables more reliable alignment among widely divergent taxa. This is especially important as PADI sequences span across bacteria, fungi, and metazoa. All sequences, intermediate alignments, and trees are provided in supplementary files 1–5, Supplementary Material online. Very strong bootstrap support (>95%) was obtained for a clade restricted to certain cyanobacterial and animal PADIs that excludes a fully supported outgroup clade containing fungal, actinobacterial, and proteobacterial sequences (supplementary fig. S5*a*–*c*, Supplementary Material online). With full branch support, the fungal and actinobacterial sequences were recovered as clades and found to be sister taxa in the tree. This tree topology, whereby animal sequences have closer affinity to those in cyanobacteria than to other eukaryotic (fungal) sequences is surprising because it is inconsistent with the known species tree.

Phylogenetic tree inferences, in particular those obtained from single genes, are subject to errors. It is possible that the observed topology represents the failure of phylogenetic inference in the case of this individual gene, such that an artifact (e.g., model misspecification) might explain the affinity of the separate eukaryotic *PADIs* to different bacterial *PADI* types. For instance, using a fixed rate matrix of amino acid substitutions to produce the tree (Jones et al. 1992; Whelan and Goldman 2001; Kalyaanamoorthy et al. 2017) can be inappropriate if there is evolutionary rate variation over different parts of the tree or deviation from typical protein substitution rates. In particular, attention has been drawn previously to heterotachous evolution, where the evolutionary substitution rate of a given site may change over time (Lopez et al. 2002). Heterotachy is particularly plausible in the case of the *PADI* gene tree because *PADI* is found in species across the tree of life (animals, fungi, cyanobacteria, actinobacteria). This could be detected if the tree topology was found to vary under different models of rate variation.

**Fig. 1.**
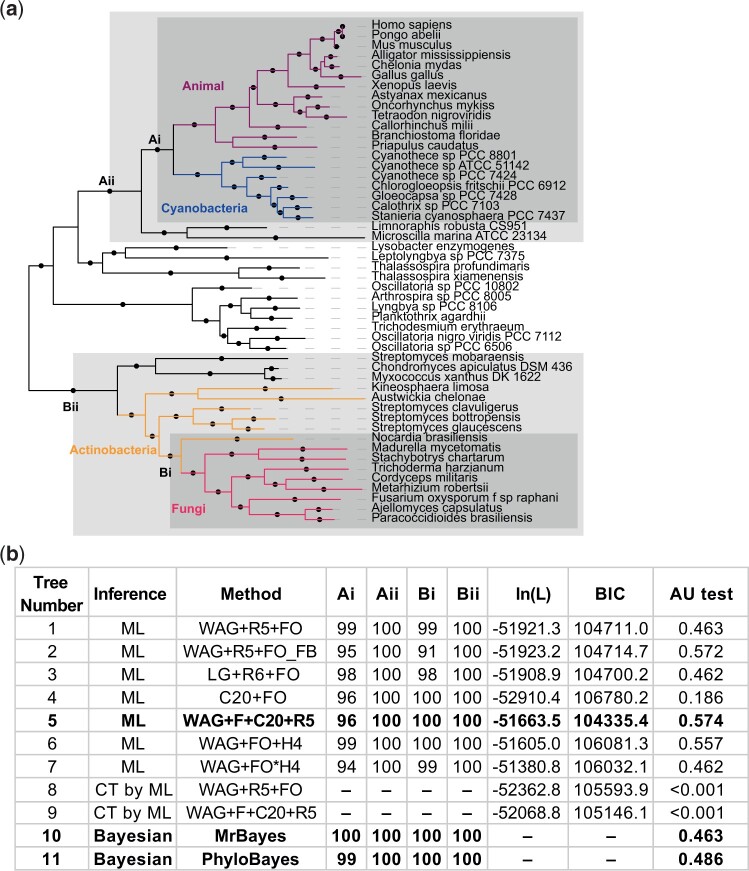
Phylogeny of the PADI sequence. (*a*) Consensus topology for all phylogenetic methods with branch lengths from Bayesian phylogenetic inference with MrBayes. Solid circles indicate consensus node support of >95%. (*b*) Summary table of the different phylogenetic analyses performed corresponding to trees shown in full in the Supplementary Material online. Ultrafast bootstrap 2 values with 1,000 replicates for trees 1, 3, 4, 5, 6, 7; Felsenstein bootstrap values with 100 replicates for tree 2; or posterior probabilities for trees 10 and 11 are presented in the table for the nodes labeled in the tree that are critical to different evolutionary scenarios. Log likelihoods and the Bayesian information criterion are presented for all maximum likelihood trees. In addition, maximum likelihood constraint trees 8 and 9 were constructed where opisthokonta were constrained to be monophyletic under the maximum likelihood models used for tree 1 and tree 5. Trees were concatenated and analyzed using the AU-test with 10,000 replicates. Nomenclature for the different models is as used in IQtree 1.6.12. The best supported maximum likelihood tree and the Bayesian trees are shown in bold.

To analyze whether our phylogenetic tree may be subject to model violation, we undertook more parameter-rich analyses on 50 sequences that were subsampled from the larger tree, and assessed their topological congruence and node support. We removed multiple paralogs in metazoa using the basal paralog PADI2 and removed sequences with close branches such that we were able to maintain the maximum sequence diversity in the tree (nine fungi, 13 metazoa, 29 bacteria). We then performed the same fixed empirical rate matrix phylogenetic analysis on the smaller set of sequences to check for congruence, before undertaking a number of phylogenetic analyses (fig. 1 and supplementary file 6, Supplementary Material online). This included a Bayesian approach that samples over different fixed empirical rate matrices (Ronquist et al. 2012); a maximum likelihood approach using a mixture model of 20 different fixed amino acid rate matrices (C20) (Quang et al. 2008); a Bayesian approach that allows for infinite mixture model categories sampled from the alignment by making use of a Dirichlet process prior (CAT-GTR) (Lartillot and Philippe 2004); and a maximum likelihood approach, designed specifically for heterotachous data sets, that allows different branch length classes across the tree (GHOST model) (Crotty et al. 2020). In addition, we produced maximum likelihood trees where eukaryotic sequences were constrained to be monophyletic under the best performing models (Trees 8 and 9, fig. 1*b* and supplementary file 7, Supplementary Material online).

All of the above analyses recovered a single topology in support of a clade of cyanobacterial and animal sequences to the exclusion of a clade of fungal and actinobacterial sequences (fig. 1*a*, clades Ai, Aii, Bi, and Bii). Posterior probabilities or bootstrap values for this topology were high, approaching 100% for each of the diverse methods (fig. 1*b*). The analysis was repeated using additional bootstrap algorithms, including the full nonparametric bootstrap, obtaining full support (Felsenstein 1985; Hoang et al. 2018). Topology constraint tests rejected a number of randomly generated trees, which confirmed the high branch support values. These alternative trees and the constrained trees for the expected model where eukaryotic PADIs are restricted to a monophyletic group were all significantly rejected (*P* < 0.001) by multiple statistical tests including the AU-test (Shimodaira 2002; Strimmer and Rambaut 2002; Susko 2014) (fig. 1*b*). We conclude that the topology of a clade of cyanobacterial and animal PADI sequences to the exclusion of fungal and actinobacterial sequences is robust to differently specified models.

### Cyanobacterial and Animal PADIs Share Unique Synapomorphies

The high bootstrap values and congruent topologies across a wide variety of methods lend strong support to our tree topology. Nevertheless, we sought to identify features of the protein sequence that may independently validate the phylogenetic topology.

**Fig. 2.**
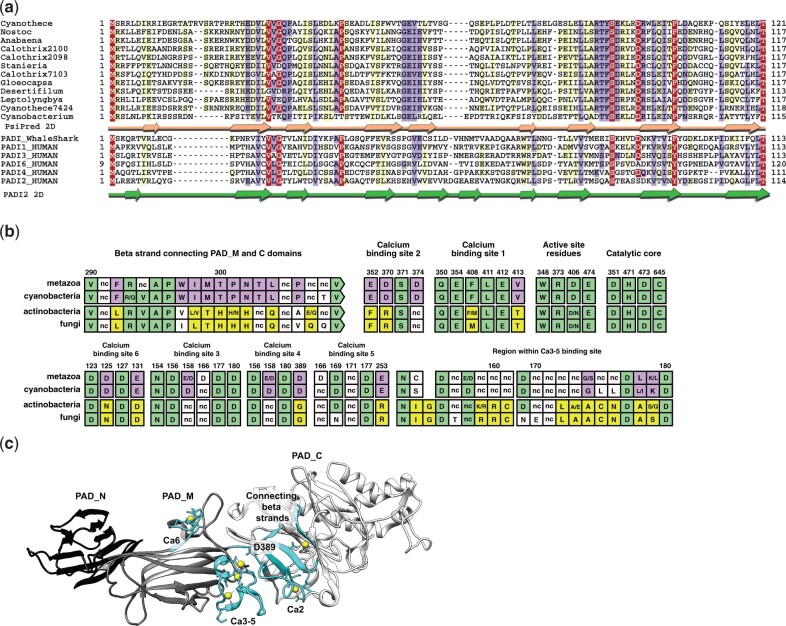
Synapomorphic features among PADI orthologs. (*a*) Alignment of putative PAD_N domains from *SPM/NX* clade cyanobacterial PADI sequences with the PAD_N domain from human PADI paralogs and *Rhincodon typus* (whale shark). The coloring scheme indicates the average BLOSUM62 scores of each alignment column: red (>3.5), violet (between 3.5 and 2), and light yellow (between 2 and 0.5). Peach arrows shown below the cyanobacterial sequences indicate PsiPred predicted secondary structure (beta sheets). Green arrows (beta sheets) correspond to the known secondary structure of the PAD_N domain of human PADI2. (*b*) Analysis of synapomorphic regions, representing six PADI sequences from each of metazoa, cyanobacteria, actinobacteria, and fungi. Consensus sites across the six species are shown with standard single letter amino acid abbreviations. “nc” (nonnonserved) represents the absence of consensus conservation to one or two amino acids across the six species. The numbering given above the alignment and corresponds to the ungapped site of human PADI2 such that residues can be compared with Slade et al. Sites showing conservation across all four domains are colored in green; sequence features common to metazoan and cyanobacterial PADIs that are excluded from fungal/actinobacterial sequences are colored in purple; sequence features common to fungal and actinobacterial PADIs that are excluded from metazoanand cyanobacterial sequences are colored in yellow. The existence of both purple and yellow sequence features is indicative of synapomorphic primary sequence features. (*c*) Crystal structure of human PADI2 presented with PAD_N domain colored in black, PAD_M domain in gray, and PAD_C domain in white. Synapomorphic regions are colored in cyan and calcium ions are shown as yellow spheres.

Firstly, we examined how the PADI protein domain architecture is distributed across orthologs using Pfam annotations, which are powered by HMMER searches (Finn et al. 2015). As mentioned above, all metazoan PADIs possess the three PADI domains, PAD_N, PAD_M, and PAD_C (supplementary fig. S1, Supplementary Material online). The cyanobacterial PADIs closest to mammalian PADIs (from *SPM* and *NX* cyanobacteria) appear to possess two Pfam-annotated domains: a PAD_M domain and a PAD_C domain, but not a PAD_N domain. By contrast, other bacterial and fungal PADIs are only annotated with the PAD_C domain. To identify domains that might have been overlooked by Pfam, we carried out more sensitive profile-to-profile HMM searches (Söding 2005; Zimmermann et al. 2018) (supplementary fig. S6*a*, Supplementary Material online). We made a multiple sequence alignment firstly of cyanobacterial species contained in the clade of metazoan sequences (fig. 1*a*, clade Ai), and secondly of the remaining bacterial and fungal sequences (fig. 1*a*, sequences outside of clade Aii). Regions corresponding to each of the PAD_N, PAD_M, and PAD_C domains from human PADI2 were extracted and searched against a database of profiles of all domains contained in Pfam. This revealed that the bacterial and fungal sequences outside Clade Aii possess a divergent version of the PAD_M domain, but do not possess a PAD_N domain: the PAD_N region is completely absent from those fungal and bacterial orthologs, including cyanobacteria diverging earlier than *SPM/NX*. By contrast, the cyanobacterial homologs contained within Clade Ai (diverging after *SPM* and *NX* clades) possess all three domains including a degenerate metazoan PAD_N cupredoxin type domain (PAD_N domain: E-value <1×10^−7^). We then identified the cyanobacterial sequence that is predicted to adopt the PAD_N secondary structure using PsiPred and aligned this with animal PAD_N sequences. The predicted cyanobacterial PAD_N sequence aligns well with the human PAD_N domain, as determined experimentally using PADI2 crystal structure data (Slade et al. 2015) (fig. 2*a*), confirming that the Clade Ai cyanobacterial PADIs possess a degenerate PAD_N domain.

Secondly, we analyzed representative fungal, actinobacterial, cyanobacterial, and metazoan PADI sequences for the conservation of calcium-binding and active site residues (fig. 2*b*). The allosteric binding of up to six calcium ions allows formation of the PADI2 active site cleft and is an absolute requirement for catalytic activity (Slade et al. 2015). All catalytic residues and substrate-binding residues are fully conserved among all PADI homologs (fig. 2*b*). In addition, calcium-binding sites 3 and 1 appear to be fully conserved, whereas calcium site 5 is also likely conserved. Calcium-binding site 6 is likely to be conserved functionally, as the substitution of D125 to N and E131 to D, which are present in both actinobacterial and fungal sequences, are expected to preserve ion binding. Intriguingly, however, calcium sites 2 and 4 appear to be exclusive to Clade Ai (late diverging cyanobacterial and metazoan) sequences. The fungal and actinobacterial sequences diverge from binding sites 2 and 4 to a different amino acid motif. Critically, only Clade Ai PADI sequences conserve the calcium switch residue D389 (residues: 369–389). In actinobacterial and fungal sequences, this residue is substituted to glycine and therefore incompetent for metal coordination (Slade et al. 2015) (fig. 2*b*). This indicates that the ordered, sequential calcium binding in the PAD_M domain, which is responsible for the allosteric communication between PAD_M and the catalytic PAD_C domain in human PADI2 (Slade et al. 2015) is likely to be conserved only in Clade Ai PADIs. As a result, a potentially different mode of calcium regulation operates in the fungal and actinobacterial PADIs.

In addition, we find that fungal and actinobacterial sequences share features that are not present in the Clade Ai PADIs. This includes a conserved region within calcium-binding sites 3–5 that is absent from the metazoan and cyanobacterial sequences (fig. 2*b*: amino acids 155–180, where differences conserved between fungal and actinobacterial sequences are highlighted in yellow). Also of interest is a highly conserved ten amino acid beta sheet that connects the PAD_M and PAD_C domains (fig. 2*b*: amino acids 292–302). This region is conserved closely in fungal and actinobacterial sequences, but to a different ten amino acid sequence containing a distinctive triple histidine motif (fig. 2*b*: amino acids 300–302).

We therefore find primary and tertiary amino acid sequences that are specific to either the cyanobacterial/metazoan or the actinobacterial/fungal PADIs. It is implausible that blocks of sequence of up to ten amino acids were derived convergently and independently in these two groups of PADIs. Thus these sequence features are indicative of a common ancestry of actinobacterial and fungal PADIs that is distinct from the ancestry of cyanobacterial and metazoan PADIs and constitute synapomorphies. The phylogenetic topology presented in figure 1 is consistent whether built with or without the above synapomorphic sequence features and PAD_N domain (supplementary fig. S6*b*, Supplementary Material online). As these features occur at the level of the amino acid sequence and at the level of a whole protein domain (fig. 2*c*), they are robust to differences in rate variation across the tree and to saturated sequence artifacts (Doolittle 1994; Zhang and Kumar 1997; Bazykin et al. 2007; Baalsrud et al. 2018). These features therefore provide strong additional support of the phylogenetic topology presented in figure 1.

### The PADI Sequence Divergence between Cyanobacteria and Animals Is Anachronistically Low

**Fig. 3.**
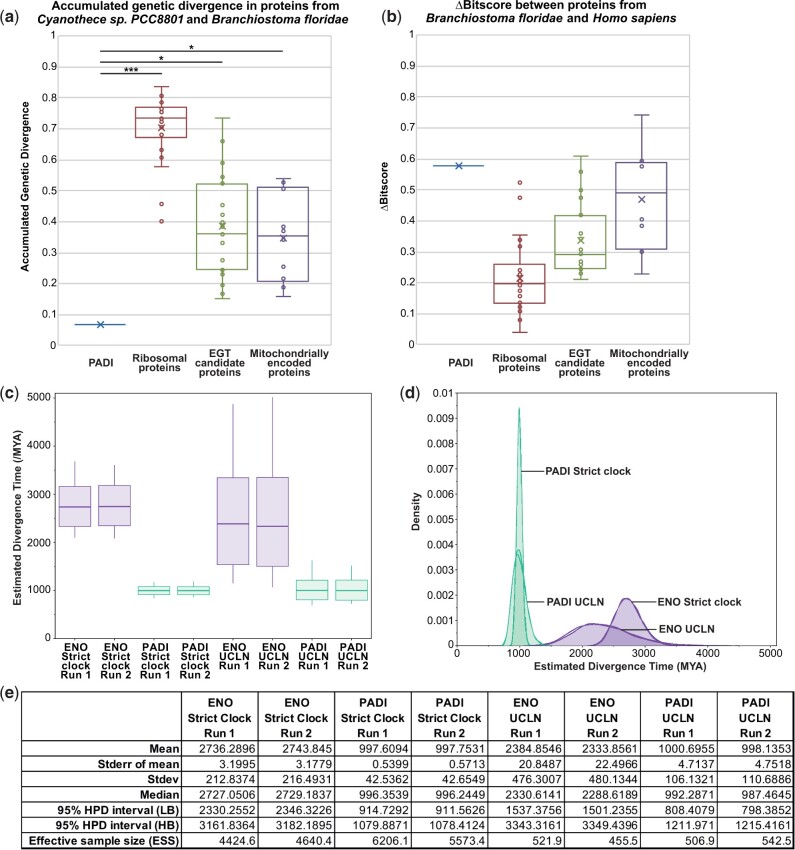
Sequence divergence analyses. (*a*, *b*) Analysis of the sequence divergence of 26 vertically transferred proteins, 19 candidate EGT proteins, and ten proteins encoded in the mitochondrial genome. (*a*) Box and whisker plot showing the calculated AGD between *Cyanothece sp. PCC 8801* and *Branchiostoma floridae* relative to *Homo sapiens*. (*b*) Box and whisker plot showing the normalized Δbitscore between *B. floridae* and *H. sapiens*. The cross represents the mean. All protein values are plotted with outliers exceeding 1.5× the interquartile range shown. The null hypothesis that PADIs fall within the normal distribution of each set of proteins was rejected with *P* < 0.0001 denoted as ***; or *P* < 0.05 denoted as *. (*c*, *d*) Estimated divergence time of late diverging *SPM/NX* clade cyanobacteria and metazoa based on their PADI sequences, as calibrated using geologically defined constraints from the fossil record. Metazoan and *SPM/NX* DNA sequences were used for Bayesian phylogenetic analysis in BEAST2 under the strict clock and the UCLN clock models. A calibrated Yule model was used as the tree prior using a GTR model with five gamma distributed rate categories. Divergence times from the fossil record were used as normally distributed node age priors centered on the median ages of six different nodes from metazoa with a sigma value covering the uncertainty of the estimate. The marginal posterior distribution of the age of the root of the whole tree was used to estimate the divergence time. (*c*) Box and whisker plot for the estimate divergence time from each analysis showing two independent runs per analysis. (*d*) Kernel density estimate for each analysis showing two independent runs per analysis. (*e*) Table of summary statistics for the estimated divergence time.

The remarkably high similarity of Clade Ai cyanobacterial and animal PADIs prompted us to examine the rate of sequence change between them in more detail. To do this, we firstly sought to understand the extent of change of PADIs relative to other highly conserved proteins in the species that bridge the closest PADI homologs. We therefore analyzed a large number of the most conserved proteins in life to approximate a mean minimum extent of accumulated genetic divergence (AGD), represented by sequence change, occurring between *Cyanothece sp. 8801* and *B. belcheri* and compared this with the divergence of the PADI sequence between these two species (fig. 3*a*). As a negative control, we compared the difference in bitscore density (Δbitscore) for these conserved proteins and for the PADI sequence between *B. belcheri* and *H. sapiens* (fig. 3*b*). We also analyzed 19 proteins of likely endosymbiont gene transfer (EGT) origin and ten proteins encoded in mitochondrial genomes. The approach used to calculate the AGD of a given protein between its homologs in *H. sapiens*, *Branchiostoma*, and *Cyanothece* is described in supplementary figure S7, Supplementary Material online. Since mitochondrial and EGT-derived proteins in metazoa may be closer to their bacterial homologs than might be expected for other vertically inherited genes, the AGD for these classes of protein may be even lower than the AGD for highly conserved ribosomal proteins. We reasoned that the AGD of mitochondrial and EGT-derived proteins may therefore mimic that of an anciently horizontally transferred gene into eukaryotes and the AGD calculated for PADIs may be even lower than these classes of proteins if it was acquired more recently than the mitochondrion (as we hypothesize for the PADI gene). Indeed, EGT and mitochondrially encoded proteins have an average AGD that is significantly lower than that of vertically acquired proteins between *Cyanothece sp. 8801* and *B. belcheri* (fig. 3*a*), but not between *B. belcheri* and *H. sapiens* (fig. 3*b*). We find that the AGD of PADI falls below that calculated for vertically transferred protein sequences, as assessed over the same timescale (fig. 3*a*), falling six SDs below that of vertically transferred protein sequences, but behaves as expected between *B. belcheri* and *H. sapiens* (fig. 3*b*). PADIs show less sequence change than all proteins individually analyzed over this timescale and less even than ribosomal RNA (see Materials and Methods). Indeed, they fall two SDs below even the mean of EGT candidate genes (fig. 3*a*). Finally, we calculated the AGD for each mitochondrially encoded protein as compared with its own closest bacterial homolog (as opposed to the homolog from *Cyanothece sp. 8801*). PADIs exhibit a lower AGD than any of the individual mitochondrially encoded proteins relative to each of their nearest bacterial homologs. With a *P* value of 0.0073 (see Materials and Methods), we reject the null hypothesis that PADIs fall within the normal distribution of AGD values calculated for mitochondrially encoded proteins relative to their closest bacterial homolog. A model of vertical descent of PADIs from bacteria, or PADI acquisition via EGT, requires that, across lineages where PADIs cannot be observed in modern genomes, in addition to the large number of independent gene losses, PADIs would have been under greater constraint than any other known sequence in life (Isenbarger et al. 2008).

We then used a Bayesian phylogenetic approach to predict the divergence time between Ai Clade cyanobacterial and animal PADI sequences under a strict molecular clock model and under an uncorrelated lognormal (UCLN) relaxed clock model, using known fossil ages of metazoans as calibrations (Drummond et al. 2006; Drummond and Suchard 2010; Bouckaert et al. 2014). In the relaxed UCLN clock model, distinct rates are given along each branch with rates drawn at random from a lognormal distribution. Under a model of descent from bacteria, or under a model of EGT, these predictions are expected to be at least as old as the last eukaryotic ancestor, since horizontal transfer is known to be common in bacteria and archaea (Betts et al. 2018). In general, the prediction of the divergence time of a node derived from analysis of a single gene would be significantly greater than the global estimate, as evolutionary rates for a single gene may be greater than the minimum in either lineage.

We performed parallel analysis on the median gene from our EGT candidates above (enolase or ENO) to provide an internal comparison for the divergence time predicted by PADI sequences and calibrations from fossil ages. Our analysis yielded an estimate of less than 1 Gy for the age of the root of the tree as estimated by PADI sequences (fig. 3*c*–*e*). Under all approaches, the divergence times were not congruent with the geologically defined divergence and were found to be 1.7 Gy (strict clock) or 1.3 Gy (UCLN relaxed clock) lower than that predicted by the ENO gene (fig. 3*c*–*e*). The upper bound of our divergence times (95% credible interval) was found to be below the lower bound of the range of globally and geographically defined estimates for the date of the LUCA (>3,900 Ma), the date for eukaryogenesis (1,866–1,679 or 1,842–1,210 Ma), and the date of the symbiotic origin of mitochondria (2,053–1,210 Ma). The use of ENO as a control is likely to be conservative as seen from its AGD, which is lower than any individual ribosomal protein (fig. 3*a*). These divergence time estimates are therefore inconsistent with vertical descent of metazoan PADIs from bacteria or with descent via EGT and are instead consistent with a horizontal acquisition event that is more recent than the acquisition of the mitochondrion by eukarya. The divergence times predicted by these clock models are approximately dated at the time of divergence of the last common ancestor of PADI-harboring metazoa.

### The Cyanobacterial PADI Protein Is Catalytically Active

**Fig. 4.**
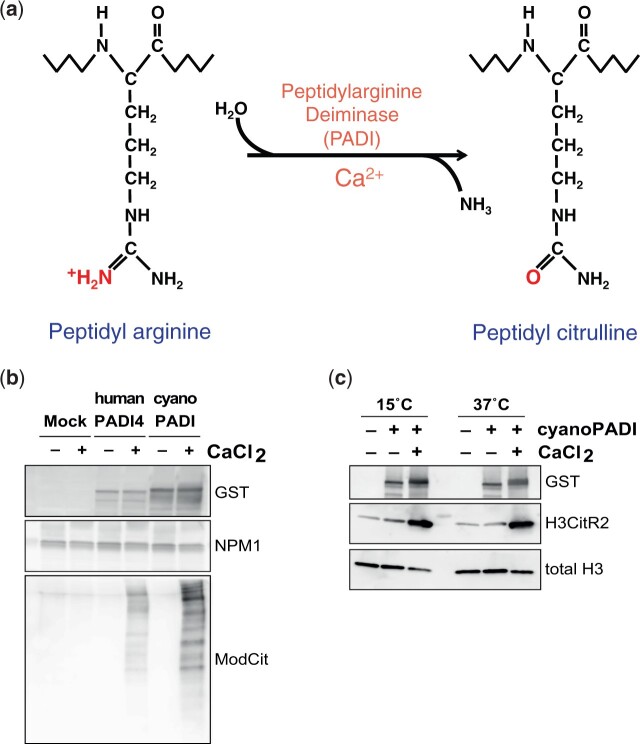
Biochemical analyses of the cyanobacterial PADI enzyme from *Cyanothece sp. 8801* (cyanoPADI). (*a*) The citrullination reaction results in the converison of a positively charged peptidyl arginine residue to a neutral peptidyl citrulline and it is carried out by PADI enzymes in a calcium-dependent manner. (*b*, *c*) Immunoblot analyses of citrullination assays using GST-His-tagged recombinant enzymes. (*b*) Whole cell lysates from mouse embryonic stem cells were used as substrate and the presence of citrullination in a protein sequence-independent manner was assessed using the ModCit antibody. Nucleophosmin (NPM1) is used as a loading control. (*c*) Recombinant human histone H3 was used as substrate and citrullination of H3 arginine 2 was assessed. Total histone H3 is used as loading control.

Considering the high degree of similarity between Clade Ai cyanobacterial and metazoan PADIs, including all necessary catalytic residues and calcium-binding residues, we hypothesized that the ancestral cyanobacterial protein is likely to be catalytically active and calcium dependent (fig. 4*a*). To test this, we prepared a recombinant version of the three-domain PADI from *Cyanothece sp. 8801* (here referred to as “cyanoPADI”) and assayed its catalytic activity alongside human PADI4. Analogously to the human enzyme, cyanoPADI can citrullinate multiple proteins in mouse cell lysates (fig. 4*b*). In addition, cyanoPADI shows absolute dependence on calcium for activity. This demonstrates that the calcium-dependent regulation found in mammalian PADIs is also a feature of the ancestral cyanobacterial protein and suggests that the conserved calcium-binding sites, which were used in the evolutionary analysis as signifiers of synapomorphy, are functional (figs. 2*b* and 4). Remarkably, and despite the absence of histones from bacteria, cyanoPADI catalyzes citrullination of histone H3 (fig. 4*c*), which is a known target of mammalian PADI4. The enzyme is additionally active at a physiologically relevant temperature for cyanobacteria (fig. 4*c*). Thus cyanoPADI is a bona fide calcium-dependent PADI with sufficient similarity or promiscuity to catalyze citrullination of mammalian substrates.

## Discussion

It has been hypothesized that very few protein modification types existed in the LUCA and these have been diversified to give rise to the >200 PTMs known today (Beltrao et al. 2013). We sought to map the evolutionary origin of citrullination, which is implicated in the regulation of a variety of physiological and pathological processes in humans. Our analyses of PADI homologs across life reveal the existence of two clearly discernible PADI types: one containing three structural domains and sharing functionally relevant sequence features and one containing two structural domains and divergent sequence features. The taxonomic distribution of these two types of homologs is highly unusual, in that three-domain PADIs are present in animal and late-diverging cyanobacteria, whereas two-domain PADIs are present in fungi and all other bacteria (figs. 1 and 2; supplementary fig. S6, Supplementary Material online). This evidence can be reconciled with vertical evolutionary descent if the last eukaryotic common ancestor (LECA) harbored two paralogous *PADI* genes which underwent widespread and mutually exclusive losses throughout evolution: firstly, the three-domain *PADI* present in late-diverging cyanobacteria and metazoa was lost from lineages leading to every other species in life; and secondly, the two-domain *PADI* present in fungi, actinobacteria, and proteobacteria must be separately accounted for in independent gene losses in lineages leading to all other species. In lineages that harbor no *PADI*, the two paralogs must have been lost independently (supplementary fig. S8, Supplementary Material online). It is notable that no species is observed to possess both PADI types.

The above scenario, although highly unparsimonious, would be supported if rates of *PADI* sequence evolution across a species phylogeny were consistent with respect to geologically defined timings and with genes well known to have been inherited vertically from bacteria or by EGT from the LECA. Our analyses of sequence divergence provide evidence to the contrary. In absolute terms, the similarity of cyanobacterial and branchiostomal PADIs to human PADIs is almost identical: 70.20% versus 70.90%, respectively, by pairwise amino acid similarity. However, a much greater amount of time has elapsed since the cyanobacterial and human genes have shared a last common ancestor than the genes from the other species pair (branchiostoma and humans). Even under assumptions of heterotachy, where rates of evolution may differ between different lineages, a minimal amount of nearly neutral genetic divergence nonetheless accumulates over evolutionary timescales in all lineages (Takahata 1996; Isenbarger et al. 2008). Under the assumption of vertical descent, the observed *PADI* sequence changes are anachronistically low even compared with the most highly conserved genomic sequences in life, including ribosomal proteins and even EGT candidates and genes encoded in the mitochondrion.

**Fig. 5.**
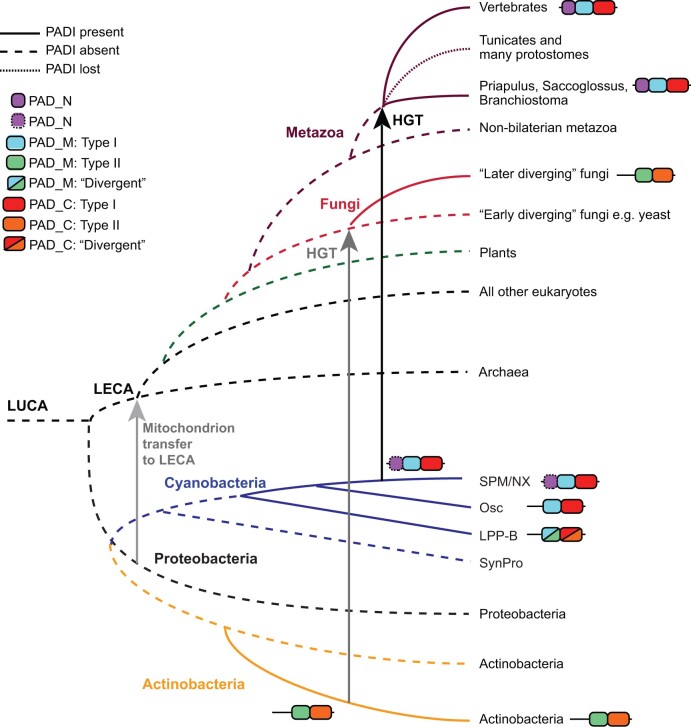
Proposed model of PADI evolution. Domain architecture is denoted in the figure legend. Horizontal transfer of the three-domain sequence from cyanobacteria to metazoa denoted by a black arrow, likely horizontal transfer of the two-domain sequence from actinobacteria to fungi denoted by a dark gray arrow and transfer of the mitochondrion to the LECA denoted by a light gray arrow. Proposed origin for the PADI sequence is within bacterial evolution and emergence of the three-domain PADI is within the *SPX/NM* cyanobacterial clade. Gene losses observed in various metazoan lineages after the HGT are indicated with a narrow dashed line.

The explanation for the observation of such little sequence change is more mundane under the assumption of horizontal transfer (fig. 5). A HGT event from late-diverging *SPM/NX* clade cyanobacteria to a last common ancestor within the animal lineage, although ancient, would have occurred much more recently than the LUCA and also more recently than the mitochondrion. HGT can therefore fully account for the phylogenetic distribution, as well as the slow rates of evolution observed. The two lines of evidence are complementary and independent. The timing of transfer (neoproterozoic: 1,000–542 Ma) is consistent with the presence of marine nitrogen fixing cyanobacteria with specialized arginine catabolic pathways (Schriek et al. 2007), and with the emergence of metazoa in the cyanobacterial habitat (Erwin et al. 2011; Yuan et al. 2011; Sánchez-Baracaldo et al. 2014). A second HGT event, from actinobacterial species that are known to be fungal pathogens, most parsimoniously explains the existence of the two-domain fungal PADI (Clade Bi in fig. 1 and supplementary fig. S6, Supplementary Material online; figs. 2 and 5). This is consistent with the absence of a *PADI* gene either in eukaryotic species diverging before opisthokonts or in early diverging fungi such as yeast.

Closer examination of *PADI* phylogeny in bacteria provides additional support for HGT and indicates the directionality of horizontal transfer (supplementary figs. S9 and S10, Supplementary Material online). Firstly, strong support is found for bacterial PADIs that form an outgroup to both the two-domain and three-domain PADI sequences (supplementary figs. S5 and S10, Supplementary Material online). These bacterial outgroup sequences suggest that PADIs were not horizontally acquired by bacteria. Secondly, the fact that the metazoan-type three-domain PADI only emerges in the late-diverging *SPM* and *NX* clades of cyanobacteria, and the cyanobacterial PADI phylogeny mirrors the expected species tree (Uyeda et al. 2016) (supplementary fig. S9, Supplementary Material online), indicates that the three-domain PADI did not exist in the LUCA. The existence of cyanobacterial outgroup sequences, with a discernable origin within bacterial evolution, specifically implies the direction of HGT of the three-domain PADI was from cyanobacteria into metazoa and not in reverse (supplementary fig. S9, Supplementary Material online).

All but one metazoan PADI sequence identified by our comprehensive searches in genomic and proteomic databases were found in deuterostomes—the exception being found in the *P. caudatus* genome, a protostome. This suggests that the HGT took place either at the root of the deuterostomes, or possibly at the root of bilateria. Note that this part of the tree of life remains poorly resolved, with an extremely short branch between the bilaterian common ancestor and the deuterostomes (Philippe et al. 2019).

Biochemical analyses of the ancestral three-domain PADI (cyanoPADI) show that it is competent for catalysis (fig. 4), whereas a recent study has identified catalytically active PADI homologs in the thermotolerant fungi *Emericella dentata* and *Aspergillus nidulans* (El-Sayed et al. 2019). The discovery of catalytically active PADI orthologs in bacteria and fungi offers fertile ground for investigation of the roles of citrullination in these organisms.

Our finding that the cyanoPADI can citrullinate mammalian substrates (fig. 4) indicates that a novel catalytic capability was added to the regulatory repertoire of metazoan cells by HGT. The newly acquired regulatory function is likely to have enhanced biochemical diversity in animals. Fish genomes contain a single *PADI* gene, but duplications resulted in five tandem repeated paralogs in mammalian genomes (Chavanas et al. 2004) (supplementary fig. S10, Supplementary Material online). The fact that these duplicated genes were retained across many animal genomes suggests that they were unlikely to be functionally redundant. In the course of vertebrate evolution, citrullination was thus expanded in scope and adapted to a variety of cellular contexts, ranging from neutrophil extracellular trap release to stem cell potency, and from oligodendrocytes to bone marrow and keratinocytes (Nicholas and Bhattacharya 2014). The emerging physiological roles of the vertebrate PADIs, such as in the regulation of pluripotency and embryonic development (Brahmajosyula and Miyake 2013; Christophorou et al. 2014; Xu et al. 2016; Xiao et al. 2017), and the newly described role of the fish PADI in tissue regeneration (Golenberg et al. 2020), point to possible selective advantages conferred to metazoans by PADIs and offer a possible explanation for the fact that PADIs were retained so widely (Huang 2013). In a similar vein, it is interesting to consider our findings in light of the proposal that genes with a role in antimicrobial defence are amenable to co-option by eukaryotic innate immune systems (Chou et al. 2015). The extent to which the molecular mechanisms that regulate the human PADIs were also conserved from cyanobacteria or were newly co-opted in vertebrates remains an intriguing open question.

It is notable that no citrullination-reversing enzyme has been identified in any species to date. The evolutionary analysis of PADIs presented here adds extra complexity as to whether the reverse catalytic process might have also arisen or been propagated. It has been postulated that “toolkits” of PTM writer, eraser, and reader enzymes may have evolved in a coordinated fashion and this has been studied formally in the context of protein phosphorylation (Lim and Pawson 2010). In this context, the investigation into potential reverse catalysis for citrullination should be extended to include bacterial and fungal enzymes.

A related consideration is prompted by the known role of PADIs in autoimmunity. It has been proposed that the exogenous citrullinating activity of pPAD at sites of periodontal infection is an initiating event in the development of rheumatoid arthritis, by predisposing individuals with prior periodontal infection to the development of autoantibodies against citrullinated endogenous proteins (Anti-Citrullinated Protein Antibodies, ACPAs) (Mikuls et al. 2012). It is therefore of note that pPAD and gADI genes are more widespread than previously thought (supplementary fig. S4, Supplementary Material online) and that the PADIs described in this paper can be found in a number of human pathogens and in *Stachybotrys chlorohalonata* (black mold). A re-evaluation of the initiating events responsible for citrullination-specific breaks in immune tolerance may therefore be warranted.

This work reveals the remarkable evolutionary trajectory of the *PADI* gene family and uncovers the origin of a protein modification with diverse functions in human physiology and disease. In combination, the pieces of evidence presented above comprise a compelling case of ancient horizontal transfer of a bacterial gene into animals.

## Materials and Methods

### Structural Analyses

Structural homology searches were performed using the Dali server v3.1 with the extracted PAD_C domain used as query (Holm and Rosenström 2010). Superposition of known structures was performed in Chimera (Pettersen et al. 2004) using the MatchMaker tool (Meng et al. 2006). Briefly, the two structures (PDB: 4n2c and 1xkn) were aligned for the best-aligning pair of chains using the Needleman–Wunsch algorithm and BLOSUM62 matrix. A secondary structure score of 30% was included. The superposition was iterated by pruning long atom pairs such that no pair exceeds 2.0 Å.

### Identification of PADI Orthologs

A graph-based unsupervised clustering algorithm used by the EggNOG database was used to infer PADI orthologous groups from 2,031 genomes across the tree of life (ENOG410ZKF3: 217 proteins from 74 species) (Huerta-Cepas, Szklarczyk, et al. 2016). Phylogenetic reconstruction for the identified PADI orthologs was performed within EggNOG as implemented within the ETE3 suite (eggnog41) and described at http://eggnogdb.embl.de/#/app/methods (last accessed June 7, 2020) (Huerta-Cepas, Serra, et al. 2016; Huerta-Cepas, Szklarczyk, et al. 2016). In addition, a list of proteins with significant similarity (E-value < 1×10^−^^3^) to the metazoan PAD_C domain from human PADI2 were collected using HMMER searches against *Reference Proteomes* and *UniProtKB* databases (Potter et al. 2018). Additional more sensitive sequence searches and iterative searches were performed using TBlastN and psiblast against nr/nt; jackhammer against reference proteomes and *UniProtKB*; and hhpred against Pfam-A, COG_KOG, and PDB_mmCIF70 (Altschul et al. 1997; Söding 2005; Alva et al. 2016). To verify the exhaustive nature of our search for PADI homologs, we employed two state-of-the-art remote homology detection tools. The first, HHBlits, is used to search databases of hidden Markov models (HMM) generated with clustered proteomic data sets with a query hmm and is available in the HHSuite (Hildebrand et al. 2009; Remmert et al. 2012). The second tool, hmmsearch, is included in the HMMER suite (Eddy 2011) and used to search proteomic data sets with an HMM. A PADI alignment was generated from our initial data set of known homologs for use with both tools using clustal omega on default parameters for three iterations to generate our query HMM. HHBlits was used to search the Uniclust30 database. Its construction and contents are detailed on the MMseqs website (Steinegger and Söding 2017) (Uniclust, cited May 5, 2020; Available from: https://uniclust.mmseqs.com/). Hmmsearch was used to search the NCBI NR protein database. Its contents and construction are detailed on the NCBI web page (Download-NCBI, cited May 5, 2020; Available from: https://www.ncbi.nlm.nih.gov/home/download/). The HHBlits search results were filtered with a cutoff of 90− probability and 50 amino acids. No additional hits were found to sequences in clades that were not in the starting data set.

#### Analysis of Spurious Viral and Archaeal Hits

The HMMsearch results were filtered with an E-value cutoff of 10^−^^10^ and 50 amino acids. Two sequences attributed to unexpected clades were found: RefSeq identifiers AXN91134.1 and RCV64870.1 which are found in *Namao virus* and *Methanophagales archaeon*, respectively. The two anomalous sequences are the only representatives of the *PADI* family within their taxonomic kingdoms and this extremely sparse distribution of these sequences would either imply many independent gene loss events or an extremely recent horizontal transfer event of *PADI* to these clades if the genes are in fact correctly attributed to their genome. To verify the validity of the attribution of these sequences to their respective genomes, we calculated a phylogeny including by aligning a subset of high confidence *PADI* sequences with the two putative homologs. The alignment was then used with IQTree (Trifinopoulos et al. 2016) on default parameters and automatic selection of the appropriate model to generate a phylogeny. The resulting tree was visualized with figtree (Rambaut 2016) (supplementary fig. S2, Supplementary Material online). The placement of the two sequences in the phylogeny does not agree well with a plausible evolutionary scenario considering their taxonomic origin; rather their placement suggests that the allegedly *Methanophagales archaeon* sequence is in fact a cyanobacterial sequence, and that the allegedly *Namao virus* sequence, is in fact a fish sequence. These hypotheses are corroborated by the origin of the samples used to obtain them: metagenomic isolates in the case of *Methanophagales archaeon*, and infected tissue samples taken from fish in the case of *Namao virus.* In both cases, the samples were susceptible to gene misattribution due to incorrect binning (Sangwan et al. 2016) or contamination. To further probe the association of these genes to their respective genomes and discount the possibility of the *PADI* genes belonging to a transferred genomic segment, we used the k-mer spectra of the genomes to study the possibility of horizontal transfer events. These analytics are regularly used to find transferred regions in prokaryotic genomes (Bernard et al. 2018). Normalized k-mer spectra for DNA sequences were generated by counting occurrences of all k-mers and normalizing by the total amount of words counted to give a unit vector. The results presented in supplementary figure S3, Supplementary Material online, were derived using 4-mers. To detect possible horizontally transferred genomic regions, an average spectrum for the entire genome was calculated. A spectrum was then calculated for a sliding window of 1 kb using 500-bp steps and subtracted from the genomic average at each window position. The absolute value of the difference between the genomic average and window spectra is represented over the entire genome. The code for running these kmer-based analyses is available at https://github.com/DessimozLab/PADI (last accessed June 7, 2020).

### Phylogenetic Methods

For all phylogenetic trees, branch support information was visualized and figures produced using FigTree v1.4.3 and iTOL (Letunic and Bork 2016). Amino acid sequences for PADI homologs were obtained from UniProtKB, NCBI, and Pathosystems Resource Integration Center (PATRIC) databases using HMMER and BLAST searches (Altschul et al. 1990; Finn et al. 2015; Wattam et al. 2017). PADI2 was used for species with multiple PADI paralogs, as it closest resembles the PADI gene in metazoa with one PADI (such as fish; György et al. 2006), and with the PADI2 from metazoan species with three PADIs such as birds or reptiles (supplementary fig. S7, Supplementary Material online).

#### Phylogenetic Analysis of Other Citrullinating Enzymes

Sequences of the arginine deiminase from *Giardia lamblia* (gADI; Touz et al. 2008) and the porphyromonas-type peptidylarginine deiminase from *Porphyromonas gingivalis* (pPAD; McGraw et al. 1999) were used as a seed for HMM searches of reference proteomes to identify sequences from other species of similar length and most significant similarity (Finn et al. 2015; Potter et al. 2018). These amino acid sequences were aligned with 25 representative PADI sequences using MAFFT L-ins-I (Katoh et al. 2018) and singly aligning columns were removed. IQTree was used to produce a maximum likelihood phylogenetic tree (Nguyen et al. 2015; Trifinopoulos et al. 2016). The LG empirical rate matrix with eight categories of rate variation under the FreeRate model (LG + R8) was used, as determined by ModelFinder (Le and Gascuel 2008; Kalyaanamoorthy et al. 2017) according to the corrected Akaike Information Criterion. The Ultrafast Bootstrap 2 with 1,000 replicates (Hoang et al. 2018), Shimodaira–Hasegawa (SH)-like approximate likelihood-ratio test (aLRT) with 1,000 replicates (Shimodaira and Hasegawa 1999, 2001; Guindon et al. 2010), and aBayes parametric tests (Anisimova et al. 2011) were used to assess node support.

#### Phylogenetic Analysis of PADI Orthologs

All PADI sequences in the UniProtKB rp55 database were obtained using HMMER and fragment sequences (<450 amino acids) were removed (Chen et al. 2011). Sequences were aligned using MAFFT L-ins-I (Katoh et al. 2018) and the alignment trimmed with TrimAL using *gappyout* settings (495 columns) (Capella-Gutiérrez et al. 2009). Bayesian phylogenetic analysis was performed using MrBayes v3.2.7 x64 using the CIPRES Gateway on XSEDE with the Markov chain Monte Carlo (MCMC) sampling different amino acid rate matrices according to their probability (Aamodelpr=mixed) and five gamma distributed rate categories to allow among site rate variation (Ronquist et al. 2012). Maximum likelihood phylogenetic analysis was performed using IQTree (WAG+R5+F) with node support tested using Ultrafast Bootstrap 2 with 1,000 replicates (Nguyen et al. 2015; Trifinopoulos et al. 2016; Hoang et al. 2018).

Meaningful statistical inference becomes challenging if the number of parameters exceeds the sample size. A useful proxy for the sample size in phylogenetic analysis is given by the number of columns in the alignment setting a constraint for the total number of taxa that can be analyzed in the single gene tree. An unbiased subsample of bacterial sequences was obtained by including all bacterial PADI sequences contained in the PATRIC database when the analysis was performed (82 taxa in total) (Wattam et al. 2017). Metazoan sequences were subsampled to maximize representation of lineages maintaining a PADI (33 in total: the five paralogs in *Homo sapiens*, *Pongo abelii*, and in *Mus musculus*, the three paralogs found in *Gallus gallus, Chelonia mydas*, and *Alligator mississipiensis*, and the single paralog found in *Xenopus laevis, Takifugu rubripes, Tetraodon nigroviridis, Astyanax mexicanus, Danio rerio*, *Oncorhynchus mykiss, Callorhinchus milii, Branchiostoma floridae*, and *Priapulus caudatus*). Finally, 35 fungal sequences were subsampled to span the range of sequence diversity with respect to the human sequence according to HMMER bitscore spanning a range of significances of fungal proteins giving E-values between 5.0×10^−^^26^ and 1.4×10^−^^46^. Sequences are provided in full in supplementary files 3 and 6, Supplementary Material online. The collected amino acid sequences were aligned using MAFFT L-ins-I and singly aligning columns were removed (1,100 columns) (Katoh et al. 2018). IQTree was used to produce maximum likelihood phylogenetic trees (Nguyen et al. 2015; Trifinopoulos et al. 2016). The WAG empirical rate matrix with ten categories of rate variation under the FreeRate model with base frequencies counted from the alignment (WAG+R10+F) was used, as determined by ModelFinder according to the corrected Akaike Information Criterion (Whelan and Goldman 2001; Kalyaanamoorthy et al. 2017). Ultrafast Bootstrap 2 with 1,000 replicates, SH-like aLRT with 1,000 replicates, and aBayes parametric tests were used to assess node support (Shimodaira and Hasegawa 1999, 2001; Guindon et al. 2010; Anisimova et al. 2011; Hoang et al. 2018). The tree is shown rooted at the midpoint with solid circles indicating consensus node support of >95%. The critical nodes for testing different evolutionary hypotheses mentioned in later analyses are labeled in full.

#### Phylogenetic Analysis of Subsampled PADI Orthologs for Topology Testing

For parameter rich analyses, 50 sequences were subsampled from the larger tree. We removed multiple paralogs in metazoa using the basal paralog PADI2 and removed sequences with close branches so as to maintain the maximum sequence diversity in the tree (9 fungi, 13 metazoa, 29 bacteria). In addition, both the closest and the most distant bacterial homologs with respect to the metazoan sequence were retained to allow for the broadest distribution of protein sequences. To assess the effect of sequence subsampling, a maximum likelihood phylogenetic tree using the fixed WAG empirical rate matrix was performed, with five categories of rate heterogeneity across sites allowed under the FreeRate model, and with base frequencies estimated by maximum likelihood (WAG+R5+FO) to check for congruence with the larger tree topology. This analysis was then repeated using the original Felsenstein bootstrap with 100 replicates.

Additional maximum likelihood phylogenetic analyses were performed using IQTree using the CIPRES Gateway on XSEDE (Quang et al. 2008) or using IQTree version 1.6.12 with node support assessed by Ultrafast Boostrap 2 with 1,000 replicates. Additional parameter rich models included the C20 mixture model of fixed empirical rate matrices with base frequencies estimated from the alignment (C20+FO), the C20 mixture model of empirical rate matrices where rate heterogeneity across sites was also relaxed according to the free rate model (WAG+F+C20+R5), the GHOST model (Crotty et al. 2020), which is specifically designed to analyze heterotachous data sets, where different classes of branch lengths are inferred across the tree (WAG+FO+H4), and the most general form of the GHOST model, where relative rate and base frequency parameters are unlinked and separated across the different branch length classes (WAG+FO*H4). In addition, a constraint tree was inferred using the WAG+F+C20+R5 model, where sequences from opisthokonta were constrained to be monophyletic. All trees were concatenated and used for topology testing in IQTree using the AU test. Log likelihoods and the Bayesian Information Criterion are presented alongside *P* values for the AU test in figure 1*b*.

Bayesian phylogenetic inference was firstly performed using MrBayes v3.2.6 x64 using CIPRES Gateway on XSEDE with mixed model MCMC jumping across different fixed empirical rate matrices and five different gamma distributed rate categories (Ronquist et al. 2012). Analysis was performed with four runs each of 1,000,000 chains. The average SD of split frequencies was observed to be <0.005, parameters all had an effective sample size (ESS) >500 and potential scale reduction factor of 1.000 (to four significant figures). The summary tree was generated with a burn-in of 25% over the runs. Posterior probability was used for node support—that is, where posterior probability was 100, the topology was congruent in every tree sampled by the MCMC after burn-in. The *aminoacid model* prior was set as “mixed” such that the MCMC jumps across different models, that is, mixture of models with fixed rate matrices. Poisson, Jones, Dayhoff, Mtrev, Mtmam, Wag, Rtrev, Cprev, Vt, and Blosum models were used and all have equal prior probability. The WAG model had posterior probability of 1.000, and SD <0.0001—and was exclusively sampled from the posterior (Whelan and Goldman 2001). This is consistent with the WAG model being identified as the best empirical matrix identified according to ModelFinder and the corrected Akaike information criterion from the maximum likelihood analysis in IQTree.

A second approach to Bayesian phylogenetic inference was performed using PhyloBayes under the CAT-GTR model (Lartillot and Philippe 2004; Lartillot et al. 2013). This is an infinite mixture model of rate matrices making use of a Dirichlet process prior. Eight chains were performed in parallel for 24 h such that more than 20,000 cycles were achieved as recommended in the PhyloBayes manual using the MRC IGMM and University of Edinburgh computing cluster Eddie3. Readpb, bpcomp, tracecomp tools in PhyloBayes and Tracer software were then used to analyze runs. Posterior consensus trees were generated for each run and were reproducible across the eight different runs. The trace plots for independent runs were also analyzed to assess for apparent stationarity aiming for an ESS of at least 100. Maxdiff was observed to be <0.1 (maxdiff = 0.06209, meandiff = 0.00330).

Tree topologies were congruent across the different methods with tree files provided in full (fig. 1*b* and supplementary files 4 and 5, Supplementary Material online). Topology testing of parameter rich models and maximum likelihood constraint trees was performed using IQTree version 1.6.12 and results are provided in figure 1*b*. Additional topology testing was performed in PAUP*4.0a163, where 100 random trees were generated along with the maximum likelihood constrained tree with fungal and metazoan sequences constrained to be monophyletic. The SH test, approximately unbiased (AU) test and expected likelihood weight (ELW) tests were performed and all other alternative trees, including the constraint tree were rejected (*P* < 0.001) (Shimodaira and Hasegawa 2001; Shimodaira 2002; Strimmer and Rambaut 2002; Susko 2014).

#### Phylogenetic Analysis Excluding Synapomorphic Regions

Phylogenetic analyses from figure 1 were repeated using an alignment with the PAD_N domain removed and with an alignment in which both the PAD_N domain and regions of synapomorphy (supplementary fig. S4, Supplementary Material online) were removed. Maximum likelihood analysis using IQTree with ModelFinder using the same best performing fixed empirical rate matrix (WAG+R5+FO) as above (Nguyen et al. 2015; Trifinopoulos et al. 2016; Kalyaanamoorthy et al. 2017; Hoang et al. 2018). Topologies were congruent with analysis of the whole alignment and node support values (Ultrafast Bootstrap 2) for the clades labeled in figure 1 are provided in supplementary figure S4*b*, Supplementary Material online.

### PADI Domain Annotation

To identify putative locations for the three PAD domains within PADI homolog sequences from bacteria and fungi, each target PADI sequence was aligned to five metazoan sequences using TCoffee (Di Tommaso et al. 2011). Putative domain sequence regions were then used as a target query for HMMER or HHPred searches (Söding et al. 2005; Finn et al. 2015). HMMER searches were made against the *UniProtKB* database and HHPred searches were performed, firstly against a database of HMM profiles of protein domains in the Protein Data Bank (PDB_mmCIF_4_Aug) and secondly, against a database of profiles from Pfam (Pfam-A_v31.0) (Alva et al. 2016). Once individual sequences were identified as possessing a specific domain architecture, multiple sequence alignments of groups of sequences with common putative domain architecture were made and these were used as queries for each type of search.

For the reported E-values in supplementary figure S4*a*, Supplementary Material online, the following method was used. All sequences from the highlighted clade in the phylogenetic tree were aligned using TCoffee. PAD_C, PAD_M, and PAD_N domains from the cyanobacterial sequences, and secondly PAD_C and PAD_M domains from the clade containing a mixture of bacterial and fungal sequences were extracted. These alignments were used as a seed for searches with HHPred against a database of profiles made of the entire human proteome, and against a database of profiles of Pfam domains (Pfam-A_v31.0). HHPred searches were performed using the MPI Bioinformatics Toolkit of the Max Planck Institute for Developmental Biology, Tübingen, Germany (Alva et al. 2016; Zimmermann et al. 2018).

### Multiple Sequence Alignment of PAD_N Domain

Amino acid sequences were aligned using the TCoffee algorithm (Edgar 2004; Di Tommaso et al. 2011) and visualized using Jalview (Waterhouse et al. 2009). Putative PAD_N domains from the *SPM*/*NX* clade cyanobacterial PADI sequences were identified using HHPred as showing significant statistical evidence for affinity (E-value: 2.5×10^−^^5^) (Alva et al. 2016; Zimmermann et al. 2018). These were aligned with the PAD_N domain from human PADI paralogs and *Rhincodon typus* (whale shark). The alignment was presented with the program Belvu using a coloring scheme indicating the average BLOSUM62 scores (which are correlated with amino acid conservation) of each alignment column (Henikoff and Henikoff 1992), as represented in figure 2*a*. PsiPred (Jones 1999) was used to predict secondary structure for the cyanobacterial PAD_N domains (beta sheets) and presented with the alignment. The experimental secondary structure of the PAD_N domain of human PADI2 was identified from the crystal structure (PDB: 4n2a) (Slade et al. 2015).

### Synapomorphy Analysis of PADI Calcium-Binding Sites

Representative fungal, actinobacterial, cyanobacterial, and metazoan PADI sequences were analyzed for the conservation of all of the calcium-binding sites (a minimum of three residues coordinate each calcium-binding site) and for other critical residues contained at the active site (fig. 2). PADIs from the following species were used: 1) metazoan PADIs from *Homo sapiens*, *Xenopus laevis*, *Oncorhynchus mykiss*, *Callorhinchus milii*, *Branchiostoma floridae*, *Priapulus caudatus*; 2) cyanobacterial PADIs from *Cyanothece sp. 8801, Stanieria cyanosphaera, Chlorogloeopsis fritschii PCC 6912, Crocosphaera subtropica, Aphanothece sacrum, Cyanothece sp. 7424;* 3) fungal PADIs from *Fusarium sp. FOSC 3-a, Periconia macrospinosa, Paracoccidioides lutzii, Blastomyces parvus, Ajellomyces capsulatus, Emmonsia crescens* and; 4) actinobacterial PADIs from *Streptomyces silvensis, Alteromonas lipolytica, Streptomyces sp. 3214.6, Erythrobacter xanthus, Kibdelosporangium aridum, Nocardia brasiliensis ATCC 700358*. Sequences were aligned using MAFFT L-ins-I and compared with functionally annotated regions from Slade et al. (2015) and from crystal structures (Arita et al. 2004; Slade et al. 2015; Katoh et al. 2018).

### Accumulated Genetic Divergence Analysis Relative to Other Proteins

Bitscore density is calculated by taking the bitscore of a query sequence to the target sequence produced by HMMER and dividing by the bitscore of the query sequence to itself (longer sequences have higher bitscores), which gives a value between 0 and 1 (Finn et al. 2015). The bitscore densities of the similarity of 1) the cyanobacterial homolog to the human sequence: ×bitscoreD_Cy-Hu_ (AC+AH) and 2) of the branchiostomal homolog to the human sequence: ΔbitscoreD_Br-Hu_ (XB+XH) were both calculated (supplementary fig. S7, Supplementary Material online). A measure of the total accumulated genetic divergence between late-diverging cyanobacteria (*Cyanothece* spp.) and the last common ancestor of *Branchiostoma* spp. and *Homo sapiens* was then calculated by subtracting ΔbitscoreD_Br-Hu_ from the ΔbitscoreD_Cy-Hu_. This accumulated genetic divergence (AGD) value was calculated for: 1) 26 ribosomal proteins (uS2, uS3, uS4, uS5, uS7, uS8, uS9, uS10, uS11, uS12, uS13, uS17, uS19, uL1, uL2, uL3, uL4, uL5, uL6, uL11, uL13, uL14, uL15, uL22, uL23, uL24), 2) 19 sequences whose proteins are mitochondrially located so are reasonable EGT candidates from the mitochondrion (OTC, ASS1, ARLY, CPS1, PGK, ENO, GAPDH, PK, NAXE, G6PD, RPIA, FUMH, SDHB, SDHA, CS, MDHM, DLAT, DLDH, ACLY) (Timmis et al. 2004), and 3) all ten proteins still encoded in the mitochondrial genome (MT-ATP6, MT-CO1, MT-CO2, MT-CO3, MT-CYB, MT-1, MT-2, MT-3, MT-4, MT-5). It is notable that by definition, only very highly conserved proteins have an AGD that can be calculated in this extreme example between the last common ancestor of late diverging cyanobacteria and humans: if a protein has diverged substantially then the similarity of the human homolog to the cyanobacterial will not be discernible and no bitscore can be calculated. AGD values of proteins in each category were tested for deviation from a normal distribution using the Shapiro–Wilk test (*W*=*b*^2^/ SS) (Shapiro and Wilk 1965). Where the calculated *P* value exceeded 0.05, the null hypothesis was retained, and the data treated as being normally distributed. Kurtosis and skew were also within the range of the normal distribution. The AGD for PADI proteins (AGD_PADI proteins_ = 0.07) was then compared with the mean AGD of each category of control proteins (e.g., AGD_ribosomal proteins_ = 0.70) and the *z*-scores were calculated and are presented as *P* values.

To compare the extent of divergence relative to ribosomal RNA (rRNA), nucleotide sequences for rRNA were obtained from the SILVA database (Yilmaz et al. 2014). Nucleotide sequences for PADIs were obtained from NCBI and exons extracted. Comparisons were made with EMBOSS Needle using the Needleman–Wunsch global alignment algorithm (Needleman and Wunsch 1970) (gap open: 10, gap extend: 0.5).

### Sequence Divergence Analyses

From the AGD analysis performed above, the median EGT candidate protein was selected as a control (ENO). Sequence divergence analysis was performed on ENO and PADI DNA sequences. BEAST v2.4.8 was used to produce a time tree of the clade of subsampled metazoan PADIs and the full clade of closest *SPM*/*NX* cyanobacteria contained within the PATRIC database using the GTR model with four gamma-distributed rate categories (Drummond and Rambaut 2007; Bouckaert et al. 2014; Uyeda et al. 2016). ENO sequences from the same species under the same model specifications as PADIs were used for the control analysis. The following metazoan species were used: *Homo sapiens* (HS), *Mus musculus* (MM), *Alligator mississippiensis* (AM), *Chelonia mydas* (CM), *Gallus gallus* (GG), *Xenopus laevis* (XL), *Oncorhynchus mykiss* (OM), *Callorhinchus milii* (CM), *Branchiostoma floridae* (BF), *Priapulus caudatus* (PC). To calibrate nodes on the tree, node times were set as the following normally distributed priors: mean 797.0, sigma 72.5 (clade of HS, MM, AM, CM, GG, XL, OM, CM, BF, PC); mean 692.5, sigma 57.5 (clade of HS, MM, AM, CM, GG, XL, OM, CM, BF); mean 473.5, sigma 14.0 (clade of HS, MM, AM, CM, GG, XL, OM, CM); mean 435.0, sigma 6.5 (clade of HS, MM, AM, CM, GG, XL, OM); mean 311.0, sigma 7.5 (clade of HS, MM, AM, CM, GG); mean 89.5, sigma 3.0 (clade of HS, MM). DNA sequences were translated in silico and sequence before the start codon and after the stop codon was removed. DNA sequences were aligned using MAFFT L-ins-I and singly aligning columns were removed. Metazoan divergence times from the fossil record were obtained from timetree.org with bounds on the distributions chosen to span the range of times reported in the literature centered on the median value (Kumar and Hedges 2011). The calibrated Yule model was used as the tree prior. XML files were generated in BEAUti and the MCMC analysis was run using BEAST2 on the CIPRES Gateway on XSEDE. An initial MCMC run of 5,000,000 chains was run for each clock model (Drummond et al. 2006; Drummond and Suchard 2010). Then analysis was performed with two independent runs of 10,000,000 chains under two different clock models—the strict clock model and the relaxed uncorrelated lognormal (UCLN) clock model. The UCLN model relaxes the strict clock by allowing rate heterogeneity across branches: each branch is assumed to have its own rate that is drawn from a shared parametric rate distribution (the log-normal distribution). The different analyses were additionally run under the tree prior (i.e., in the absence of sequence data). Analysis of parameters was performed in Tracer to assess apparent stationarity for the different tree parameters and for acceptable ESS values and congruence was assessed across the independent runs. The predicted divergence time of the metazoan and cyanobacterial clades was given by the marginal posterior distribution of the age of the root of the whole tree. This is given by the TreeHeight parameter. These data were plotted with the kernel density estimate against the TreeHeight parameter for the different runs and summarized in a box and whisker plot. Summary data for the TreeHeight parameter are provided in figure 3*E* and include the highest posterior density 95% credible interval.

In calculating AGD for each mitochondrially encoded protein as compared with its own closest bacterial homolog (as opposed to the homolog from *Cyanothece sp 8801*), we tested for normality using the Shapiro–Wilk test (*P* = 0.109), retaining the null hypothesis that the points are normally distributed. We then calculate the z-statistic for the PADI AGD to its nearest homolog (*z*=−2.439). This corresponds to a *P* value = 0.0073. With a *P* value of 0.0073, we therefore reject the null hypothesis that PADIs fall within the normal distribution of AGD values calculated for mitochondrially encoded proteins relative to their closest bacterial homolog.

### Preparation of Recombinant Proteins

PADI gene sequences were obtained from NCBI and synthesized by Thermo GeneArt with flanking EcoRI (at the 5′ end) and XhoI (at the 3′ end) restriction sites. *Cyanothece sp. 8801* PADI and human PADI4 sequences were subcloned into a modified pGEX vector, which included an additional 10× His tag immediately N-terminal of the enzyme sequence (generous gift from Dr Martin Reijns, MRC Human Genetics Unit), by InFusion cloning. GST-His-PADI4 and GST-His-cyanoPADI were expressed in BL21 (DE3) in 2TY cultures. Cells were grown (37 °C; 180 rpm) to an OD_600_ of 0.6 and induced overnight at 18 °C with 0.5 mM β-D-1-thiogalactopyranoside (IPTG). Bacterial pellets were harvested by centrifugation (8,000×g; 10 min) and frozen at −80 °C. Cell pellets were resuspended in 50 mM Tris pH 7.5, 500 mM NaCl, 20 mM imidazole, 5% glycerol, 1 mM DTT (1 g dry cell mass in 4 ml), 1× EDTA-free protease inhibitors (Roche), 5 mM MgCl_2_, and 10 units benzonase at 4 °C with stirring. Cells were lysed on ice by sonication (7×45 s, with 45 s breaks) and the lysate was cleared by centrifugation (20,000×g; 20 min). Supernatant was sterile filtered (0.2 μm) before loading by Superloop onto a 5 ml HisTrap column, which was pre-equilibrated with binding buffer. Proteins were purified using an AKTA FPLC system (GE Healthcare). The column was washed with 50 mM Tris pH 7.5, 500 mM NaCl, 25 mM imidazole, 5% glycerol, 1 mM DTT, and the recombinant proteins were eluted with 50 mM Tris pH 7.5, 500 mM NaCl, 250 mM imidazole, 5% glycerol, 1 mM DTT. The purified sample was concentrated using Vivaspin MWCO filters into 50 mM HEPES pH 7.5, 150 mM NaCl, 5 mM DTT, 5% (v/v) glycerol, and the concentration determined using Nanodrop.

### Citrullination Activity Assays

#### Using Mouse Cell Lysates

E14 mouse embryonic stem cells were cultured in GMEM supplemented with 10% fetal calf serum, 0.1 mM nonessential amino acids, 2 mM L-glutamine, 1 mM sodium pyruvate, 0.1 mM beta-mercaptoethanol, and 10^6^ units/l leukemia inhibitory factor (ESGRO, Millipore) and grown on a six well plate until 70% confluent. Cells were harvested in 0.5% NP-40, 20 mM Tris pH 7.6, 1× EDTA-free protease inhibitors, 5 mM DTT, after two washes in PBS (one in PBS containing 2 mM EDTA and one in plain PBS). To shear chromatin and clarify lysates, benzonase and 2 mM MgCl_2_ were added and samples were rotated at 4 °C for 30 min, sheared by passing through a 25G needle and centrifuged at 20,000xg for 5 min. Citrullination activity assays were performed with 500 nM recombinant enzyme in 50 mM HEPES pH 7.5, 150 mM NaCl, 5 mM DTT, 5% (v/v) glycerol, either in the presence of 5 mM CaCl_2_ or water. Reactions were incubated for 30 min at 37 °C and quenched by boiling at 95 °C for 5 min. Samples were stored at −80 °C before immunoblotting.

#### Using Recombinant Histone H3 Substrate

Reactions were performed in 50 mM HEPES, 137 mM NaCl, 5 mM DTT with 1.5 μM recombinant H3 (New England Biolabs), vehicle or 500 nM recombinant enzyme, and in the presence of either 5 mM CaCl_2_ or water. Reactions incubated for 30 min at 15 °C or 37 °C and quenched by boiling at 95 °C for 5 min before immunoblotting.

### Immunoblotting

Proteins were separated by SDS–PAGE and transferred to nitrocellulose membrane using wet transfer. Membranes were blocked in 5% BSA in TBS containing 0.1% Tween-20 for 1 h at room temperature. Proteins were detected using primary antibodies against anti-H3 (Abcam ab10799, 1:1,000), anti-H3CitR2 (Abcam ab176843, 1:2,000), anti-NPM1 (Abcam ab37659, 1:200), and anti-GST (Abcam ab19256, 1:1,000) overnight at 4 °C and in secondary antibody at 1:5,000 for 1 h at room temperature. Membranes were incubated in Pierce ECL reagent and imaged using ImageQuant LAS 4000 (GE). Citrulline-containing proteins were modified on the membrane and detected using the antimodified citrulline detection kit (Millipore, 17-347) according to manufacturer’s instructions.

## Supplementary Material

Supplementary data are available at *Molecular Biology and Evolution* online.

## Supplementary Material

msab317_Supplementary_Data

## References

[msab317-B91] Altschul SF , Gish W, Miller W, Myers EW, Lipman DJ. 1990. Basic local alignment search tool. J Mol Biol. 215(3):403–410. 2231712 10.1016/S0022-2836(05)80360-2

[msab317-B92] Altschul SF , Madden TL, Schäffer AA, Zhang J, Zhang Z, Miller W, Lipman DJ. 1997. Gapped BLAST and PSI-BLAST: a new generation of protein database search programs. Nucleic Acids Res. 25(17):3389–3402. 9254694 10.1093/nar/25.17.3389 PMC146917

[msab317-B93] Alva V , Nam SZ, Söding J, Lupas AN. 2016. The MPI bioinformatics Toolkit as an integrative platform for advanced protein sequence and structure analysis. Nucleic Acids Res. 44(W1):W410–W415. 27131380 10.1093/nar/gkw348 PMC4987908

[msab317-B94] Anisimova M , Gil M, Dufayard JF, Dessimoz C, Gascuel O. 2011. Survey of branch support methods demonstrates accuracy, power, and robustness of fast likelihood-based approximation schemes. Syst Biol. 60(5):685–699. 21540409 10.1093/sysbio/syr041 PMC3158332

[msab317-B1] Arita K , Hashimoto H, Shimizu T, Nakashima K, Yamada M, Sato M. 2004. Structural basis for Ca2+-induced activation of human PAD4. Nat Struct Mol Biol. 11(8):777–783. 15247907 10.1038/nsmb799

[msab317-B2] Baalsrud HT , Tørresen OK, Solbakken MH, Salzburger W, Hanel R, Jakobsen KS, Jentoft S. 2018. De novo gene evolution of antifreeze glycoproteins in codfishes revealed by whole genome sequence data. Mol Biol Evol. 35(3):593–606. 29216381 10.1093/molbev/msx311 PMC5850335

[msab317-B3] Balandraud N , Gouret P, Danchin EGJ, Blanc M, Zinn D, Roudier J, Pontarotti P. 2005. A rigorous method for multigenic families’ functional annotation: the peptidyl arginine deiminase (PADs) proteins family example. BMC Genomics 6:153. 16271148 10.1186/1471-2164-6-153 PMC1310624

[msab317-B4] Bazykin GA , Kondrashov FA, Brudno M, Poliakov A, Dubchak I, Kondrashov AS. 2007. Extensive parallelism in protein evolution. Biol Direct. 2:20. 17705846 10.1186/1745-6150-2-20 PMC2020468

[msab317-B5] Beltrao P , Bork P, Krogan NJ, Van Noort V. 2013. Evolution and functional cross-talk of protein post-translational modifications. Mol Syst Biol. 9(1):714. 24366814 10.1002/msb.201304521 PMC4019982

[msab317-B6] Betts HC , Puttick MN, Clark JW, Williams TA, Donoghue PCJ, Pisani D. 2018. Integrated genomic and fossil evidence illuminates life’s early evolution and eukaryote origin. Nat Ecol Evol. 2(10):1556–1562. 30127539 10.1038/s41559-018-0644-x PMC6152910

[msab317-B95] Bernard G , Greenfield P, Ragan MA, Chan CX. 2018. k-mer similarity, networks of microbial genomes, and taxonomic rank. mSystems. 3(6):e00257–18. 30505941 10.1128/mSystems.00257-18 PMC6247013

[msab317-B7] Boto L. 2014. Horizontal gene transfer in the acquisition of novel traits by metazoans. Proc R Soc B Biol Sci. 281(1777):20132450. 10.1098/rspb.2013.2450 PMC3896011 24403327

[msab317-B8] Bouckaert R , Heled J, Kühnert D, Vaughan T, Wu CH, Xie D, Suchard MA, Rambaut A, Drummond AJ. 2014. BEAST 2: a software platform for Bayesian evolutionary analysis. PLoS Comput Biol. 10(4):e1003537. 24722319 10.1371/journal.pcbi.1003537 PMC3985171

[msab317-B9] Brahmajosyula M , Miyake M. 2013. Role of peptidylarginine deiminase 4 (PAD4) in pig parthenogenetic preimplantation embryonic development. Zygote 21(4):385–393. 22793990 10.1017/S0967199412000160

[msab317-B96] Capella-Gutiérrez S , Silla-Martínez JM, Gabaldón T. 2009. trimAl: a tool for automated alignment trimming in large-scale phylogenetic analyses. Bioinformatics. 25(15):1972–1973. 19505945 10.1093/bioinformatics/btp348 PMC2712344

[msab317-B10] Chavanas S , Méchin MC, Takahara H, Kawada A, Nachat R, Serre G, Simon M. 2004. Comparative analysis of the mouse and human peptidylarginine deiminase gene clusters reveals highly conserved non-coding segments and a new human gene, *PADI6*. Gene 330:19–27. 15087120 10.1016/j.gene.2003.12.038

[msab317-B97] Chen C , Natale DA, Finn RD, Huang H, Zhang J, Wu CH, Mazumder R. 2011. Representative proteomesz: a stable, scalable and unbiased proteome set for sequence analysis and functional annotation. PLoS One. 6(4):e18910. 21556138 10.1371/journal.pone.0018910 PMC3083393

[msab317-B11] Chou S , Daugherty MD, Peterson SB, Biboy J, Yang Y, Jutras BL, Fritz-Laylin LK, Ferrin MA, Harding BN, Jacobs-Wagner C, et al 2015. Transferred interbacterial antagonism genes augment eukaryotic innate immune function. Nature 518(7537):98–101. 25470067 10.1038/nature13965 PMC4713192

[msab317-B12] Christophorou MA , Castelo-Branco G, Halley-Stott RP, Oliveira CS, Loos R, Radzisheuskaya A, Mowen KA, Bertone P, Silva JCR, Zernicka-Goetz M, et al 2014. Citrullination regulates pluripotency and histone H1 binding to chromatin. Nature 507(7490):104–108. 24463520 10.1038/nature12942 PMC4843970

[msab317-B13] Crisp A , Boschetti C, Perry M, Tunnacliffe A, Micklem G. 2015. Expression of multiple horizontally acquired genes is a hallmark of both vertebrate and invertebrate genomes. Genome Biol. 16(1):50. 25785303 10.1186/s13059-015-0607-3 PMC4358723

[msab317-B14] Crotty SM , Minh BQ, Bean NG, Holland BR, Tuke J, Jermiin LS, Von Haeseler A. 2020. GHOST: recovering historical signal from heterotachously evolved sequence alignments. Syst Biol. 69(2):249–264. 31364711 10.1093/sysbio/syz051

[msab317-B128] Di Tommaso P , Moretti S, Xenarios I, Orobitg M, Montanyola A, Chang JM, Taly JF, Notredame C. 2011. T-Coffee: a web server for the multiple sequence alignment of protein and RNA sequences using structural information and homology extension. Nucleic Acids Res. 39(Suppl 2):W13–W17. 21558174 10.1093/nar/gkr245 PMC3125728

[msab317-B15] Doolittle RF. 1994. Convergent evolution: the need to be explicit. Trends Biochem Sci. 19(1):15–18. 8140615 10.1016/0968-0004(94)90167-8

[msab317-B16] Drummond AJ , Ho SYW, Phillips MJ, Rambaut A. 2006. Relaxed phylogenetics and dating with confidence. PLoS Biol. 4(5):e88. 16683862 10.1371/journal.pbio.0040088 PMC1395354

[msab317-B98] Drummond AJ , Rambaut A. 2007. BEAST: Bayesian evolutionary analysis by sampling trees. BMC Evol Biol. 7:214. 17996036 10.1186/1471-2148-7-214 PMC2247476

[msab317-B17] Drummond AJ , Suchard MA. 2010. Bayesian random local clocks, or one rate to rule them all. BMC Biol. 8:114. 20807414 10.1186/1741-7007-8-114 PMC2949620

[msab317-B18] Dunning Hotopp JC. 2011. Horizontal gene transfer between bacteria and animals. Trends Genet. 27(4):157–163. 21334091 10.1016/j.tig.2011.01.005 PMC3068243

[msab317-B19] Dunning Hotopp JC. 2018. Grafting or pruning in the animal tree: lateral gene transfer and gene loss? BMC Genomics 19(1):470. 10.1186/s12864-018-4832-5 PMC6006793 29914363

[msab317-B99] Eddy SR. 2011. Accelerated profile HMM searches. PLoS Comput Biol. 7(10):e1002195. 22039361 10.1371/journal.pcbi.1002195 PMC3197634

[msab317-B100] Edgar RC. 2004. MUSCLE: multiple sequence alignment with high accuracy and high throughput. Nucleic Acids Res. 32(5):1792–1797. 15034147 10.1093/nar/gkh340 PMC390337

[msab317-B20] El-Sayed ASA , Shindia AA, AbouZaid AA, Yassin AM, Ali GS, Sitohy MZ. 2019. Biochemical characterization of peptidylarginine deiminase-like orthologs from thermotolerant *Emericella dentata* and *Aspergillus nidulans*. Enzyme Microb Technol. 124:41–53. 30797478 10.1016/j.enzmictec.2019.02.004

[msab317-B21] Erwin DH , Laflamme M, Tweedt SM, Sperling EA, Pisani D, Peterson KJ. 2011. The Cambrian conundrum: early divergence and later ecological success in the early history of animals. Science 334(6059):1091–1097. 22116879 10.1126/science.1206375

[msab317-B22] Falcão AM , Meijer M, Scaglione A, Rinwa P, Agirre E, Liang J, Larsen SC, Heskol A, Frawley R, Klingener M, et al 2019. PAD2-mediated citrullination contributes to efficient oligodendrocyte differentiation and myelination. Cell Rep. 27(4):1090–1102. 31018126 10.1016/j.celrep.2019.03.108 PMC6486480

[msab317-B23] Felsenstein J. 1985. Confidence limits on phylogenies: an approach using the bootstrap. Evolution 39(4):783. 28561359 10.1111/j.1558-5646.1985.tb00420.x

[msab317-B24] Finn RD , Clements J, Arndt W, Miller BL, Wheeler TJ, Schreiber F, Bateman A, Eddy SR. 2015. HMMER web server: 2015 update. Nucleic Acids Res. 43(W1):W30–W38. 25943547 10.1093/nar/gkv397 PMC4489315

[msab317-B25] Gladyshev EA , Meselson M, Arkhipova IR. 2008. Massive horizontal gene transfer in bdelloid rotifers. Science 320(5880):1210–1213. 18511688 10.1126/science.1156407

[msab317-B26] Golenberg N , Squirrell JM, Bennin DA, Rindy J, Pistono PE, Eliceiri KW, Shelef MA, Kang J, Huttenlocher A. 2020. Citrullination regulates wound responses and tissue regeneration in zebrafish. J Cell Biol. 219(4):e201908164. 32328635 10.1083/jcb.201908164 PMC7147109

[msab317-B27] Goulas T , Mizgalska D, Garcia-Ferrer I, Kantyka T, Guevara T, Szmigielski B, Sroka A, Millan C, Uson I, Veillard F, et al 2015. Structure and mechanism of a bacterial host-protein citrullinating virulence factor, *Porphyromonas gingivalis* peptidylarginine deiminase. Sci Rep. 5:11969. 26132828 10.1038/srep11969 PMC4487231

[msab317-B101] Guindon S , Dufayard JF, Lefort V, Anisimova M, Hordijk W, Gascuel O. 2010. New algorithms and methods to estimate maximum-likelihood phylogenies: assessing the performance of PhyML 3.0. Syst Biol. 9(3):307–321. 10.1093/sysbio/syq010 20525638

[msab317-B28] Guo Q , Fast W. 2011. Citrullination of inhibitor of growth 4 (ING4) by peptidylarginine deminase 4 (PAD4) disrupts the interaction between ING4 and p53. J Biol Chem. 286(19):17069–17078. 21454715 10.1074/jbc.M111.230961 PMC3089551

[msab317-B102] György B , Tóth E, Tarcsa E, Falus A, Buzás EI. 2006. Citrullination: a posttranslational modification in health and disease. Int J Biochem Cell Biol. 38(10):1662–1677. 16730216 10.1016/j.biocel.2006.03.008

[msab317-B103] Henikoff S , Henikoff JG. 1992. Amino acid substitution matrices from protein blocks. Proc Natl Acad Sci U S A. 89(22):10915–10919. 1438297 10.1073/pnas.89.22.10915 PMC50453

[msab317-B104] Hildebrand A , Remmert M, Biegert A, Söding J. 2009. Fast and accurate automatic structure prediction with HHpred. Proteins Struct Proteins. 77(Suppl 9):128–132. 10.1002/prot.22499 19626712

[msab317-B29] Hoang DT , Chernomor O, Von Haeseler A, Minh BQ, Vinh LS. 2018. UFBoot2: improving the ultrafast bootstrap approximation. Mol Biol Evol. 35(2):518–522. 29077904 10.1093/molbev/msx281 PMC5850222

[msab317-B105] Holm L , Rosenström P. 2010. Dali server: conservation mapping in 3D. Nucleic Acids Res. 38(Suppl 2):W545–W549. 20457744 10.1093/nar/gkq366 PMC2896194

[msab317-B30] Hochstrasser M. 2009. Origin and function of ubiquitin-like proteins. Nature 458(7237):422–429. 19325621 10.1038/nature07958 PMC2819001

[msab317-B31] Huang J. 2013. Horizontal gene transfer in eukaryotes: the weak-link model. Bioessays 35(10):868–875. 24037739 10.1002/bies.201300007 PMC4033532

[msab317-B106] Huerta-Cepas J , Serra F, Bork P. 2016. ETE 3: reconstruction, analysis, and visualization of phylogenomic data. Mol Biol Evol. 33(6):1635–1638. 26921390 10.1093/molbev/msw046 PMC4868116

[msab317-B32] Huerta-Cepas J , Szklarczyk D, Forslund K, Cook H, Heller D, Walter MC, Rattei T, Mende DR, Sunagawa S, Kuhn M, et al 2016. EGGNOG 4.5: a hierarchical orthology framework with improved functional annotations for eukaryotic, prokaryotic and viral sequences. Nucleic Acids Res. 44(D1):D286–D293. 26582926 10.1093/nar/gkv1248 PMC4702882

[msab317-B33] Husnik F , McCutcheon JP. 2018. Functional horizontal gene transfer from bacteria to eukaryotes. Nat Rev Microbiol. 16(2):67–79. 29176581 10.1038/nrmicro.2017.137

[msab317-B34] Isenbarger TA , Carr CE, Johnson SS, Finney M, Church GM, Gilbert W, Zuber MT, Ruvkun G. 2008. The most conserved genome segments for life detection on earth and other planets. Orig Life Evol Biosph. 38(6):517–533. 18853276 10.1007/s11084-008-9148-z

[msab317-B35] Iyer LM , Burroughs AM, Aravind L. 2008. Unraveling the biochemistry and provenance of pupylation: a prokaryotic analog of ubiquitination. Biol Direct. 3:45. 18980670 10.1186/1745-6150-3-45 PMC2588565

[msab317-B36] Jensen L , Grant JR, Laughinghouse HD, Katz LA. 2016. Assessing the effects of a sequestered germline on interdomain lateral gene transfer in Metazoa. Evolution 70(6):1322–1333. 27139503 10.1111/evo.12935

[msab317-B37] Jones DT , Taylor WR, Thornton JM. 1992. The rapid generation of mutation data matrices from protein sequences. Bioinformatics 8(3):275–282. 10.1093/bioinformatics/8.3.275 1633570

[msab317-B107] Jones DT. 1999. Protein secondary structure prediction based on position-specific scoring matrices. J Mol Biol. 292(2):195–202. 10493868 10.1006/jmbi.1999.3091

[msab317-B38] Kalyaanamoorthy S , Minh BQ, Wong TKF, Von Haeseler A, Jermiin LS. 2017. ModelFinder: fast model selection for accurate phylogenetic estimates. Nat Methods. 14(6):587–589. 28481363 10.1038/nmeth.4285 PMC5453245

[msab317-B108] Katoh K , Rozewicki J, Yamada KD. 2018. MAFFT online service: multiple sequence alignment, interactive sequence choice and visualization. Brief Bioinform. 20(4):1160–1166. 10.1093/bib/bbx108 PMC6781576 28968734

[msab317-B39] Keeling PJ , Palmer JD. 2008. Horizontal gene transfer in eukaryotic evolution. Nat Rev Genet. 9(8):605–618. 18591983 10.1038/nrg2386

[msab317-B40] Koonin EV. 2010. The origin and early evolution of eukaryotes in the light of phylogenomics. Genome Biol. 11(5):209. 20441612 10.1186/gb-2010-11-5-209 PMC2898073

[msab317-B41] Koonin EV , Makarova KS, Aravind L. 2001. Horizontal gene transfer in prokaryotes: quantification and classification. Annu Rev Microbiol. 55:709–742. 11544372 10.1146/annurev.micro.55.1.709 PMC4781227

[msab317-B109] Kumar S , Hedges SB. 2011. Timetree2: species divergence times on the iPhone. Bioinformatics. 27(14):2023–2024. 21622662 10.1093/bioinformatics/btr315 PMC3129528

[msab317-B42] Lacroix B , Citovsky V. 2016. Transfer of DNA from bacteria to eukaryotes. MBio. 7(4):e00863–16. 27406565 10.1128/mBio.00863-16 PMC4958254

[msab317-B43] Lartillot N , Philippe H. 2004. A Bayesian mixture model for across-site heterogeneities in the amino-acid replacement process. Mol Biol Evol. 21(6):1095–1109. 15014145 10.1093/molbev/msh112

[msab317-B110] Lartillot N , Rodrigue N, Stubbs D, Richer J. 2013. Phylobayes mpi: phylogenetic reconstruction with infinite mixtures of profiles in a parallel environment. Syst Biol. 62(4):611–615. 23564032 10.1093/sysbio/syt022

[msab317-B111] Le SQ , Gascuel O. 2008. An improved general amino acid replacement matrix. Mol Biol Evol. 25(7):1307–1320. 18367465 10.1093/molbev/msn067

[msab317-B44] Leger MM , Eme L, Stairs CW, Roger AJ. 2018. Demystifying eukaryote lateral gene transfer. BioEssays 40(5):1700242. 10.1002/bies.201700242 29543982

[msab317-B112] Letunic I , Bork P. 2016. Interactive tree of life (iTOL) v3: an online tool for the display and annotation of phylogenetic and other trees. Nucleic Acids Res. 44(W1):W242–W245. 27095192 10.1093/nar/gkw290 PMC4987883

[msab317-B45] Lim WA , Pawson T. 2010. Phosphotyrosine signaling: evolving a new cellular communication system. Cell 142(5):661–667. 20813250 10.1016/j.cell.2010.08.023 PMC2950826

[msab317-B46] Linsky T , Fast W. 2010. Mechanistic similarity and diversity among the guanidine-modifying members of the pentein superfamily. Biochim Biophys Acta Proteins Proteomics. 1804(10):1943–1953. 10.1016/j.bbapap.2010.07.016 PMC4104755 20654741

[msab317-B47] Lopez P , Casane D, Philippe H. 2002. Heterotachy, an important process of protein evolution. Mol Biol Evol. 19(1):1–7. 11752184 10.1093/oxfordjournals.molbev.a003973

[msab317-B48] Macek B , Forchhammer K, Hardouin J, Weber-Ban E, Grangeasse C, Mijakovic I. 2019. Protein post-translational modifications in bacteria. Nat Rev Microbiol. 17(11):651–664. 31485032 10.1038/s41579-019-0243-0

[msab317-B49] Martin WF. 2017. Too much eukaryote LGT. BioEssays 39(12):1700115. 10.1002/bies.201700115 29068466

[msab317-B113] McGraw WT , Potempa J, Farley D, Travis J. 1999. Purification, characterization, and sequence analysis of a potential virulence factor from *Porphyromonas gingivalis*, peptidylarginine deiminase. Infect Immun. 67(7):3248–3256. 10377098 10.1128/iai.67.7.3248-3256.1999 PMC116503

[msab317-B114] Meng EC , Pettersen EF, Couch GS, Huang CC, Ferrin TE. 2006. Tools for integrated sequence-structure analysis with UCSF Chimera. BMC Bioinformatics. 7:339. 16836757 10.1186/1471-2105-7-339 PMC1570152

[msab317-B50] Mikuls TR , Thiele GM, Deane KD, Payne JB, O’Dell JR, Sayles H, Weisman MH, Gregersen PK, Buckner JH, et al 2012. *Porphyromonas gingivalis* and disease-related autoantibodies in individuals at increased risk of rheumatoid arthritis. Arthritis Rheum. 64(11):3522–3530. 22736291 10.1002/art.34595 PMC3467347

[msab317-B51] Moran NA , Jarvik T. 2010. Lateral transfer of genes from fungi underlies carotenoid production in aphids. Science 328(5978):624–627. 20431015 10.1126/science.1187113

[msab317-B52] Musse AA , Li Z, Ackerley CA, Bienzle D, Lei H, Poma R, Harauz G, Moscarello MA, Mastronardi FG. 2008. Peptidylarginine deiminase 2 (PAD2) expression in a transgenic mouse leads to specific central nervous system (CNS) myelin instability. Dis Model Mech. 1(4–5):229–240. 19093029 10.1242/dmm.000729 PMC2590822

[msab317-B115] Needleman SB , Wunsch CD. 1970. A general method applicable to the search for similarities in the amino acid sequence of two proteins. J Mol Biol. 48(3):443–453. 5420325 10.1016/0022-2836(70)90057-4

[msab317-B116] Nguyen LT , Schmidt HA, Von Haeseler A, Minh BQ. 2015. IQ-TREE: a fast and effective stochastic algorithm for estimating maximum-likelihood phylogenies. Mol Biol Evol. 32(1):268–274. 25371430 10.1093/molbev/msu300 PMC4271533

[msab317-B53] Nicholas AP , Bhattacharya SK. 2014. Protein deimination in human health and disease. New York, Heidelberg, Dordrecht, London: Springer.

[msab317-B54] Ochman H , Lawrence JG, Groisman EA. 2000. Lateral gene transfer and the nature of bacterial innovation. Nature 405(6784):299–304. 10830951 10.1038/35012500

[msab317-B55] Pearce MJ , Mintseris J, Ferreyra J, Gygi SP, Darwin KH. 2008. Ubiquitin-like protein involved in the proteasome pathway of *Mycobacterium tuberculosis*. Science 322(5904):1104–1107. 18832610 10.1126/science.1163885 PMC2698935

[msab317-B117] Pettersen EF , Goddard TD, Huang CC, Couch GS, Greenblatt DM, Meng EC, Ferrin TE. 2004. UCSF chimera – a visualization system for exploratory research and analysis. J Comput Chem. 5(13):1605–1612. 10.1002/jcc.20084 15264254

[msab317-B56] Philippe H , Poustka AJ, Chiodin M, Hoff KJ, Dessimoz C, Tomiczek B, Schiffer PH, Müller S, Domman D, Horn M. 2019. Mitigating anticipated effects of systematic errors supports sister-group relationship between Xenacoelomorpha and Ambulacraria. Curr Biol. 29(11):1818–1826. 31104936 10.1016/j.cub.2019.04.009

[msab317-B118] Potter SC , Luciani A, Eddy SR, Park Y, Lopez R, Finn RD. 2018. HMMER web server: 2018 update. Nucleic Acids Res. 46(W1):W200–W204. 29905871 10.1093/nar/gky448 PMC6030962

[msab317-B57] Quang LS , Gascuel O, Lartillot N. 2008. Empirical profile mixture models for phylogenetic reconstruction. Bioinformatics 24(20):2317–2323. 18718941 10.1093/bioinformatics/btn445

[msab317-B119] Rambaut A. 2016. FigTree, version 1.4.3. Edinburgh (United Kingdom): The University of Edinburgh.

[msab317-B120] Remmert M , Biegert A, Hauser A, Söding J. 2012. HHblits: lightning-fast iterative protein sequence searching by HMM-HMM alignment. Nat Methods. 9(2):173–175. 10.1038/nmeth.1818 22198341

[msab317-B58] Ronquist F , Teslenko M, Van Der Mark P, Ayres DL, Darling A, Höhna S, Larget B, Liu L, Suchard MA, Huelsenbeck JP. 2012. MrBayes 3.2: efficient Bayesian phylogenetic inference and model choice across a large model space. Syst Biol. 61(3):539–542. 22357727 10.1093/sysbio/sys029 PMC3329765

[msab317-B59] Salzberg SL. 2017. Horizontal gene transfer is not a hallmark of the human genome. Genome Biol. 18(1):85. 28482857 10.1186/s13059-017-1214-2 PMC5422933

[msab317-B60] Sánchez-Baracaldo P , Ridgwell A, Raven JA. 2014. A neoproterozoic transition in the marine nitrogen cycle. Curr Biol. 24(6):652–657. 24583016 10.1016/j.cub.2014.01.041

[msab317-B121] Sangwan N , Xia F, Gilbert JA. 2016. Recovering complete and draft population genomes from metagenome datasets. Microbiome. 4:8. 26951112 10.1186/s40168-016-0154-5 PMC4782286

[msab317-B61] Schriek S , Rückert C, Staiger D, Pistorius EK, Michel KP. 2007. Bioinformatic evaluation of L-arginine catabolic pathways in 24 cyanobacteria and transcriptional analysis of genes encoding enzymes of L-arginine catabolism in the cyanobacterium *Synechocystis sp. PCC 6803*. BMC Genomics 8:437. 18045455 10.1186/1471-2164-8-437 PMC2242806

[msab317-B122] Shapiro SS , Wilk MB. 1965. An analysis of variance test for normality (complete samples). Biometrika. 52(3/4):591.

[msab317-B62] Sharma P , Lioutas A, Fernandez-Fuentes N, Quilez J, Carbonell-Caballero J, Wright RHG, Di Vona C, Le Dily F, Schüller R, Eick D, et al 2019. Arginine citrullination at the C-terminal domain controls RNA polymerase II transcription. Mol Cell. 73(1):84–96.e7. 30472187 10.1016/j.molcel.2018.10.016

[msab317-B63] Shimodaira H. 2002. An approximately unbiased test of phylogenetic tree selection. Syst Biol. 51(3):492–508. 12079646 10.1080/10635150290069913

[msab317-B123] Shimodaira H , Hasegawa M. 1999. Multiple comparisons of log-likelihoods with applications to phylogenetic inference. Mol Biol Evol. 16(8):1114.

[msab317-B124] Shimodaira H , Hasegawa M. 2001. CONSEL: for assessing the confidence of phylogenetic tree selection. Bioinformatics. 17(12):1246–1247. 11751242 10.1093/bioinformatics/17.12.1246

[msab317-B64] Shirai H , Blundell TL, Mizuguchi K. 2001. A novel superfamily of enzymes that catalyze the modification of guanidino groups. Trends Biochem Sci.26(8):465–468. 11504612 10.1016/s0968-0004(01)01906-5

[msab317-B65] Slade DJ , Fang P, Dreyton CJ, Zhang Y, Fuhrmann J, Rempel D, Bax BD, Coonrod SA, Lewis HD, Guo M, et al 2015. Protein arginine deiminase 2 binds calcium in an ordered fashion: implications for inhibitor design. ACS Chem Biol. 10(4):1043–1053. 25621824 10.1021/cb500933j PMC4569063

[msab317-B66] Snijders AP , Hautbergue GM, Bloom A, Williamson JC, Minshull TC, Phillips HL, Mihaylov SR, Gjerde DT, Hornby DP, Wilson SA, et al 2015. Arginine methylation and citrullination of splicing factor proline- and glutamine-rich (SFPQ/PSF) regulates its association with mRNA. RNA 21(3):347–359. 25605962 10.1261/rna.045138.114 PMC4338332

[msab317-B67] Söding J. 2005. Protein homology detection by HMM-HMM comparison. Bioinformatics. 21(7):951–960. 15531603 10.1093/bioinformatics/bti125

[msab317-B125] Söding J , Biegert A, Lupas AN. 2005. The HHpred interactive server for protein homology detection and structure prediction. Nucleic Acids Res. 33(Suppl 2):W244–W248. 15980461 10.1093/nar/gki408 PMC1160169

[msab317-B68] Soucy SM , Huang J, Gogarten JP. 2015. Horizontal gene transfer: building the web of life. Nat Rev Genet. 16(8):472–482. 26184597 10.1038/nrg3962

[msab317-B69] Stadler SC , Vincent CT, Fedorov VD, Patsialou A, Cherrington BD, Wakshlag JJ, Mohanan S, Zee BM, Zhang X, Garcia BA, et al 2013. Dysregulation of PAD4-mediated citrullination of nuclear GSK3β activates TGF-β signaling and induces epithelialto-mesenchymal transition in breast cancer cells. Proc Natl Acad Sci U S A. 110(29):11851–11856. 23818587 10.1073/pnas.1308362110 PMC3718105

[msab317-B70] Stamatakis A , Kozlov AM, Kozlov A. 2020. Efficient maximum likelihood tree building methods. In: Scornavacca C, Delsuc F, Galtier N, editors. Phylogenetics in the genomic era. p. 1.2:1–1.2:18.

[msab317-B71] Stanhope MJ , Lupas A, Italia MJ, Koretke KK, Volker C, Brown JR. 2001. Phylogenetic analyses do not support horizontal gene transfers from bacteria to vertebrates. Nature 411(6840):940–944. 11418856 10.1038/35082058

[msab317-B126] Steinegger M , Söding J. 2017. MMseqs2 enables sensitive protein sequence searching for the analysis of massive data sets. Nat Biotechnol. 35(11):1026–1028. 29035372 10.1038/nbt.3988

[msab317-B72] Strimmer K , Rambaut A. 2002. Inferring confidence sets of possibly misspecified gene trees. Proc R Soc B Biol Sci. 269(1487):137–142. 10.1098/rspb.2001.1862 PMC1690879 11798428

[msab317-B73] Sugawara K , Oikawa Y, Ouchi T. 1982. Identification and properties of peptidylarginine deiminase from rabbit skeletal muscle. J Biochem. 91(3):1065–1071. 7076645 10.1093/oxfordjournals.jbchem.a133755

[msab317-B74] Susko E. 2014. Tests for two trees using likelihood methods. Mol Biol Evol. 31(4):1029–1039. 24401182 10.1093/molbev/msu039

[msab317-B75] Suzuki A , Yamada R, Chang X, Tokuhiro S, Sawada T, Suzuki M, Nagasaki M, Nakayama-Hamada M, Kawaida R, Ono M, et al 2003. Functional haplotypes of *PADI4*, encoding citrullinating enzyme peptidylarginine deiminase 4, are associated with rheumatoid arthritis. Nat Genet. 34(4):395–402. 12833157 10.1038/ng1206

[msab317-B76] Takahata N. 1996. Neutral theory of molecular evolution. Curr Opin Genet Dev. 6(6):767–772. 8994850 10.1016/s0959-437x(96)80034-7

[msab317-B77] Tanikawa C , Ueda K, Nakagawa H, Yoshida N, Nakamura Y, Matsuda K. 2009. Regulation of protein citrullination through p53/PADI4 Network in DNA damage response. Cancer Res. 69(22):8761–8769. 19843866 10.1158/0008-5472.CAN-09-2280

[msab317-B78] Tanikawa C , Ueda K, Suzuki A, Iida A, Nakamura R, Atsuta N, Tohnai G, Sobue G, Saichi N, Momozawa Y, et al 2018. Citrullination of RGG motifs in FET proteins by PAD4 regulates protein aggregation and ALS susceptibility. Cell Rep. 22(6):1473–1483. 29425503 10.1016/j.celrep.2018.01.031

[msab317-B127] Timmis JN , Ayliff MA, Huang CY, Martin W. 2004. Endosymbiotic gene transfer: organelle genomes forge eukaryotic chromosomes. Nat Rev Genet. 5(2):123–135. 14735123 10.1038/nrg1271

[msab317-B79] Touz MC , Rópolo AS, Rivero MR, Vranych CV, Conrad JT, Svard SG, Nash TE. 2008. Arginine deiminase has multiple regulatory roles in the biology of *Giardia lamblia*. J Cell Sci. 121(17):2930–2938. 18697833 10.1242/jcs.026963 PMC2631563

[msab317-B129] Trifinopoulos J , Nguyen LT, von Haeseler A, Minh BQ. 2016. W-IQ-TREE: a fast online phylogenetic tool for maximum likelihood analysis. Nucleic Acids Res. 44(W1):W232–W235. 27084950 10.1093/nar/gkw256 PMC4987875

[msab317-B80] Uyeda JC , Harmon LJ, Blank CE. 2016. A comprehensive study of cyanobacterial morphological and ecological evolutionary dynamics through deep geologic time. PLoS One 11(9):e0162539. 27649395 10.1371/journal.pone.0162539 PMC5029880

[msab317-B130] Waterhouse AM , Procter JB, Martin DMA, Clamp M, Barton GJ. 2009. Jalview version 2 – a multiple sequence alignment editor and analysis workbench. Bioinformatics 25(9):1189–1191. 19151095 10.1093/bioinformatics/btp033 PMC2672624

[msab317-B131] Wattam AR , Davis JJ, Assaf R, Boisvert S, Brettin T, Bun C, Conrad N, Dietrich EM, Disz T, Gabbard JL, et al 2017. Improvements to PATRIC, the all-bacterial bioinformatics database and analysis resource center. Nucleic Acids Res. 45(D1):D535–D542. 27899627 10.1093/nar/gkw1017 PMC5210524

[msab317-B81] Wang S , Wang Y. 2013. Peptidylarginine deiminases in citrullination, gene regulation, health and pathogenesis. Biochim Biophys Acta. 1829(10):1126–1135. 23860259 10.1016/j.bbagrm.2013.07.003 PMC3775966

[msab317-B82] Wang Y , Li M, Stadler S, Correll S, Li P, Wang D, Hayama R, Leonelli L, Han H, Grigoryev SA, et al 2009. Histone hypercitrullination mediates chromatin decondensation and neutrophil extracellular trap formation. J Cell Biol. 184(2):205–213. 19153223 10.1083/jcb.200806072 PMC2654299

[msab317-B83] Whelan S , Goldman N. 2001. A general empirical model of protein evolution derived from multiple protein families using a maximum-likelihood approach. Mol Biol Evol. 18(5):691–699. 11319253 10.1093/oxfordjournals.molbev.a003851

[msab317-B84] Xiao S , Lu J, Sridhar B, Cao X, Yu P, Zhao T, Chen CC, McDee D, Sloofman L, Wang Y, et al 2017. SMARCAD1 contributes to the regulation of naive pluripotency by interacting with histone citrullination. Cell Rep. 18(13):3117–3128. 28355564 10.1016/j.celrep.2017.02.070 PMC5466819

[msab317-B85] Xu Y , Shi Y, Fu J, Yu M, Feng R, Sang Q, Liang B, Chen B, Qu R, Li B, et al 2016. Mutations in *PADI6* cause female infertility characterized by early embryonic arrest. Am J Hum Genet. 99(3):744–752. 27545678 10.1016/j.ajhg.2016.06.024 PMC5010645

[msab317-B132] Yilmaz P , Parfrey LW, Yarza P, Gerken J, Pruesse E, Quast C, Schweer T, Peplies J, Ludwig W, Glöckner FO. 2014. The SILVA and “all-species Living Tree Project (LTP)” taxonomic frameworks. Nucleic Acids Res. 42(D1):D643–D648. 24293649 10.1093/nar/gkt1209 PMC3965112

[msab317-B86] Yuan X , Chen Z, Xiao S, Zhou C, Hua H. 2011. An early Ediacaran assemblage of macroscopic and morphologically differentiated eukaryotes. Nature 470(7334):390–393. 21331041 10.1038/nature09810

[msab317-B87] Yuzhalin AE , Gordon-Weeks AN, Tognoli ML, Jones K, Markelc B, Konietzny R, Fischer R, Muth A, O’Neill E, Thompson PR, et al 2018. Colorectal cancer liver metastatic growth depends on PAD4-driven citrullination of the extracellular matrix. Nat Commun. 9(1):4783. 30429478 10.1038/s41467-018-07306-7 PMC6235861

[msab317-B88] Zhang J , Kumar S. 1997. Detection of convergent and parallel evolution at the amino acid sequence level. Mol Biol Evol. 14(5):527–536. 9159930 10.1093/oxfordjournals.molbev.a025789

[msab317-B89] Zhang X , Gamble MJ, Stadler S, Cherrington BD, Causey CP, Thompson PR, Roberson MS, Kraus WL, Coonrod SA. 2011. Genome-wide analysis reveals PADI4 cooperates with Elk-1 to activate C-Fos expression in breast cancer cells. PLoS Genet. 7(6):e1002112. 21655091 10.1371/journal.pgen.1002112 PMC3107201

[msab317-B90] Zimmermann L , Stephens A, Nam SZ, Rau D, Kübler J, Lozajic M, Gabler F, Söding J, Lupas AN, Alva V. 2018. A completely reimplemented MPI bioinformatics toolkit with a new HHpred server at its core. J Mol Biol. 430(15):2237–2243. 29258817 10.1016/j.jmb.2017.12.007

