# Supplementary material for: Citrullination Was Introduced into Animals by Horizontal Gene Transfer from Cyanobacteria
Source: Mol Biol Evol. 2021 Nov 3;39(2):msab317. doi: 10.1093/molbev/msab317 (PMC8826395; doi:10.1093/molbev/msab317)
Supplement: msab317_Supplementary_Data [file msab317_supplementary_data.zip › Cummings_PADI evolution_Methods_FINAL.docx]

**Materials and Methods:**

***Structural analyses***

Structural homology searches were performed using the Dali server v3.1 with the extracted PAD_C domain used as query (Holm and Rosenström 2010). Superposition of known structures was performed in Chimera (Pettersen et al. 2004) using the MatchMaker tool (Meng et al. 2006). Briefly, the two structures (PDB: 4n2c and 1xkn) were aligned for the best-aligning pair of chains using the Needleman-Wunsch algorithm and BLOSUM62 matrix. A secondary structure score of 30% was included. The superposition was iterated by pruning long atom pairs such that no pair exceeds 2.0 angstroms.

***Identification of PADI orthologues***

A graph-based unsupervised clustering algorithm used by the EggNOG database was used to infer PADI orthologous groups from 2031 genomes across the tree of life (ENOG410ZKF3: 217 proteins from 74 species) (Huerta-Cepas, Szklarczyk, et al. 2016). Phylogenetic reconstruction for the identified PADI orthologues was performed within EggNOG as implemented within the ETE3 suite (eggnog41) and described at <http://eggnogdb.embl.de/#/app/methods> (Huerta-Cepas, Szklarczyk, et al. 2016; Huerta-Cepas, Serra, et al. 2016). In addition, a list of proteins with significant similarity (E-value < 1x10^-3^) to the metazoan PAD_C domain from human PADI2 were collected using HMMER searches against *Reference Proteomes* and *UniProtKB* databases (Potter et al. 2018). Additional more sensitive sequence searches and iterative searches were performed using tblastn and psiblast against nr/nt; jackhammer against reference proteomes and *UniProtKB*; and hhpred against Pfam-A, COG_KOG and PDB_mmCIF70 (Altschul et al. 1997; Söding 2005; Alva et al. 2016). To verify the exhaustive nature of our search for PADI homologues we employed two state of the art remote homology detection tools. The first, HHBlits, is used to search databases of Hidden Markov models (HMM) generated with clustered proteomic datasets with a query hmm and is available in the HHSuite (Hildebrand et al. 2009; Remmert et al. 2012). The second tool, hmmsearch, is included in the HMMER suite (Eddy 2011) and used to search proteomic datasets with an HMM. A PADI alignment was generated from our initial dataset of known homologues for use with both tools using clustal omega on default parameters for 3 iterations to generate our query HMM. HHBlits was used to search the Uniclust30 database. Its construction and contents are detailed on the MMseqs website (Steinegger and Söding 2017) ([Uniclust. [cited 5 May 2020]. Available:](http://paperpile.com/b/RO4le9/vH3S) <https://uniclust.mmseqs.com/>). Hmmsearch was used to search the NCBI NR protein database. Its contents and construction are detailed on the NCBI web page ([Download-NCBI. [cited 5 May 2020]. Available:](http://paperpile.com/b/RO4le9/hJ8T) <https://www.ncbi.nlm.nih.gov/home/download/>). The HHBlits search results were filtered with a cutoff of 90% probability and 50 amino acids. No additional hits were found to sequences in clades that were not in the starting dataset.

***Analysis of spurious viral and archaeal hits:*** The HMMsearch results were filtered with an E-value cutoff of 10^-10^ and 50 amino acids. Two sequences attributed to unexpected clades were found: RefSeq identifiers AXN91134.1 and RCV64870.1 which are found in *Namao virus* and *Methanophagales archaeon*, respectively. The two anomalous sequences are the only representatives of the *PADI* family within their taxonomic kingdoms and this extremely sparse distribution of these sequences would either imply many independent gene loss events or an extremely recent horizontal transfer event of *PADI* to these clades if the genes are in fact correctly attributed to their genome. To verify the validity of the attribution of these sequences to their respective genomes, we calculated a phylogeny including by aligning a subset of high confidence *PADI* sequences with the two putative homologues. The alignment was then used with IQTree (Trifinopoulos et al. 2016) on default parameters and automatic selection of the appropriate model to generate a phylogeny. The resulting tree was visualized with figtree (Rambaut 2016) (Figure S2). The placement of the two sequences in the phylogeny does not agree well with a plausible evolutionary scenario considering their taxonomic origin; rather their placement suggests that the allegedly *Methanophagales archaeon* sequence is in fact a cyanobacterial sequence, and that the allegedly *Namao virus* sequence, is in fact a fish sequence. These hypotheses are corroborated by the origin of the samples used to obtain them: metagenomic isolates in the case of *Methanophagales archaeon,* and infected tissue samples taken from fish in the case of *Namao virus.* In both cases, the sample were susceptible to gene misattribution due to incorrect binning (Sangwan et al. 2016) or contamination. To further probe the association of these genes to their respective genomes and discount the possibility of the *PADI* genes belonging to a transferred genomic segment, we used the k-mer spectra of the genomes to study the possibility of horizontal transfer events. These analytics are regularly used to find transferred regions in prokaryotic genomes (Bernard et al. 2018). Normalized k-mer spectra for DNA sequences were generated by counting occurrences of all k-mers and normalizing by the total amount of words counted to give a unit vector. The results presented in Figure S3 were derived using 4-mers. To detect possible horizontally transferred genomic regions, an average spectrum for the entire genome was calculated. A spectrum was then calculated for a sliding window of 1 kb using 500bp steps and subtracted from the genomic average at each window position. The absolute value of the difference between the genomic average and window spectra is represented over the entire genome. The code for running these kmer-based analyses is available at https://github.com/DessimozLab/PADI.

***Phylogenetic methods***

For all phylogenetic trees, branch support information was visualised and figures produced using FigTree v1.4.3 and iTOL (Letunic and Bork 2016). Amino acid sequences for PADI homologues were obtained from UniProtKB, NCBI and Pathosystems Resource Integration Center (PATRIC) databases using HMMER and BLAST searches (Altschul et al. 1990; Finn et al. 2015; Wattam et al. 2017). PADI2 was used for species with multiple PADI paralogues, as it closest resembles the PADI gene in metazoa with one PADI (such as fish (György et al. 2006)), and with the PADI2 from metazoan species with three PADIs such as birds or reptiles (Figure S7).

***Phylogenetic analysis of other citrullinating enzymes*:** Sequences of the arginine deiminase from *Giardia lamblia* (gADI (Touz et al. 2008)) and the porphyromonas-type peptidylarginine deiminase from *Porphyromonas gingivalis* (pPAD (McGraw et al. 1999)) were used as a seed for Hidden Markov Model (HMM) searches of reference proteomes to identify sequences from other species of similar length and most significant similarity (Finn et al. 2015; Potter et al. 2018). These amino acid sequences were aligned with 25 representative PADI sequences using MAFFT L-ins-I (Katoh et al. 2018) and singly aligning columns were removed. IQTree was used to produce a maximum likelihood phylogenetic tree (Nguyen et al. 2015; Trifinopoulos et al. 2016). The LG empirical rate matrix with 8 categories of rate variation under the FreeRate model (LG +R8) was used, as determined by ModelFinder (Le and Gascuel 2008; Kalyaanamoorthy et al. 2017) according to the corrected Akaike Information Criterion. The Ultrafast Bootstrap 2 with 1000 replicates (Hoang et al. 2018), Shimodaira-Hasegawa (SH)-like approximate likelihood-ratio test (aLRT) with 1000 replicates (Shimodaira and Hasegawa 1999; Shimodaira and Hasegawa 2001; Guindon et al. 2010), and aBayes parametric tests (Anisimova et al. 2011) were used to assess node support.

***Phylogenetic analysis of PADI orthologues:*** All PADI sequences in the UniProtKB rp55 database were obtained using HMMER and fragment sequences (<450 amino acids) were removed (Chen et al. 2011). Sequences were aligned using MAFFT L-ins-I (Katoh et al. 2018) and the alignment trimmed with TrimAL using *gappyout* settings (495 columns)(Capella-Gutiérrez et al. 2009). Bayesian phylogenetic analysis was performed using MrBayes v3.2.7 x64 using the CIPRES Gateway on XSEDE with the MCMC sampling different amino acid rate matrices according to their probability (Aamodelpr=mixed) and 5 gamma distributed rate categories to allow among site rate variation (Ronquist et al. 2012). Maximum likelihood phylogenetic analysis was performed using IQTree (WAG+R5+F) with node support tested using Ultrafast Bootstrap 2 with 1000 replicates (Hoang et al. 2018; Nguyen et al. 2015; Trifinopoulos et al. 2016).

Meaningful statistical inference becomes challenging if the number of parameters exceeds the sample size. A useful proxy for the sample size in phylogenetic analysis is given by the number of columns in the alignment setting a constraint for the total number of taxa that can be analysed in the single gene tree. An unbiased subsample of bacterial sequences was obtained by including all bacterial PADI sequences contained in the PATRIC database when the analysis was performed (82 taxa in total) (Wattam et al. 2017). Metazoan sequences were subsampled to maximise representation of lineages maintaining a PADI (33 in total: the 5 paralogues in *Homo sapiens*, *Pongo abeli*i and in *Mus musculus*, the 3 paralogues found in *Gallus gallus, Chelonia mydas* and *Alligator mississipiensis*, and the single paralogue found in *Xenopus laevis, Takifugu rubripes, Tetraodon nigroviridis, Astyanax mexicanus, Danio rerio*, *Oncorhynchus mykiss, Callorhinchus milii, Branchiostoma floridae* and *Priapulus caudatus*). Finally, 35 fungal sequences were subsampled to span the range of sequence diversity with respect to the human sequence according to HMMER bitscore spanning a range of significances of fungal proteins giving E values between 5.0x10^-26^ and 1.4x10^-46^. Sequences are provided in full in Supplementary Files 3 and 6. The collected amino acid sequences were aligned using MAFFT L-ins-I and singly aligning columns were removed (1100 columns) (Katoh et al. 2018). IQTree was used to produce maximum likelihood phylogenetic trees (Nguyen et al. 2015; Trifinopoulos et al. 2016). The WAG empirical rate matrix with 10 categories of rate variation under the FreeRate model with base frequencies counted from the alignment (WAG+R10+F) was used, as determined by ModelFinder according to the corrected Akaike Information Criterion (Whelan and Goldman 2001; Kalyaanamoorthy et al. 2017). Ultrafast Bootstrap 2 with 1000 replicates, SH-like aLRT with 1000 replicates, and aBayes parametric tests were used to assess node support (Shimodaira and Hasegawa 1999; Shimodaira and Hasegawa 2001; Guindon et al. 2010; Anisimova et al. 2011; Hoang et al. 2018). The tree is shown rooted at the midpoint with solid circles indicating consensus node support of >95%. The critical nodes for testing different evolutionary hypotheses mentioned in later analyses are labelled in full.

***Phylogenetic analysis of subsampled PADI orthologues for topology testing:*** For parameter rich analyses, 50 sequences were subsampled from the larger tree. We removed multiple paralogues in metazoa using the basal paralogue PADI2 and removed sequences with close branches so as to maintain the maximum sequence diversity in the tree (9 fungi, 13 metazoa, 29 bacteria). In addition, both the closest and the most distant bacterial homologues with respect to the metazoan sequence were retained to allow for the broadest distribution of protein sequences. To assess the effect of sequence subsampling, a maximum likelihood phylogenetic tree using the fixed WAG empirical rate matrix was performed, with five categories of rate heterogeneity across sites allowed under the FreeRate model, and with base frequencies estimated by maximum likelihood (WAG+R5+FO) to check for congruence with the larger tree topology. This analysis was then repeated using the original Felsenstein bootstrap with 100 replicates.

Additional maximum likelihood phylogenetic analyses were performed using IQTree using the CIPRES Gateway on XSEDE (Quang et al. 2008) or using IQTree version 1.6.12 with node support assessed by Ultrafast Boostrap 2 with 1000 replicates. Additional parameter rich models included the C20 mixture model of fixed empirical rate matrices with base frequencies estimated from the alignment (C20+FO), the C20 mixture model of empirical rate matrices where rate heterogeneity across sites was also relaxed according to the free rate model (WAG+F+C20+R5), the GHOST model (Crotty et al. 2020), which is specifically designed to analyse heterotachous datasets, where different classes of branch lengths are inferred across the tree (WAG+FO+H4), and the most general form of the GHOST model, where relative rate and base frequency parameters are unlinked and separated across the different branch length classes (WAG+FO*H4). In addition, a constraint tree was inferred using the WAG+F+C20+R5 model, where sequences from opisthokonta were constrained to be monophyletic. All trees were concatenated and used for topology testing in IQTree using the AU test. Log likelihoods and the Bayesian Information Criterion are presented alongside p values for the AU test in Figure 1b.

Bayesian phylogenetic inference was firstly performed using MrBayes v3.2.6 x64 using CIPRES Gateway on XSEDE with mixed model MCMC jumping across different fixed empirical rate matrices and 5 different gamma distributed rate categories (Ronquist et al. 2012). Analysis was performed with 4 runs each of 1000000 chains. The average standard deviation of split frequencies was observed to be <0.005, parameters all had an effective sample size (ESS) > 500 and potential scale reduction factor (PSRF) of 1.000 (to 4 significant figures). The summary tree was generated with a burn-in of 25% over the runs. Posterior probability was used for node support– i.e. where posterior probability was 100, the topology was congruent in every tree sampled by the Markov chain Monte Carlo (MCMC) after burn-in. The *aminoacid model* prior was set as ‘mixed’ such that the MCMC jumps across different models i.e. mixture of models with fixed rate matrices. Poisson, Jones, Dayhoff, Mtrev, Mtmam, Wag, Rtrev, Cprev, Vt and Blosum models were used and all have equal prior probability. The WAG model had posterior probability of 1.000, and standard deviation <0.0001 – and was exclusively sampled from the posterior (Whelan and Goldman 2001). This is consistent with the WAG model being identified as the best empirical matrix identified according to ModelFinder and the corrected Akaike information criterion from the maximum likelihood analysis in IQTree.

A second approach to Bayesian phylogenetic inference was performed using PhyloBayes under the CAT-GTR model (Lartillot and Philippe 2004; Lartillot et al. 2013). This is an infinite mixture model of rate matrices making use of a Dirichlet process prior. Eight chains were performed in parallel for 24 hours such that more than 20000 cycles were achieved as recommended in the PhyloBayes manual using the MRC IGMM and University of Edinburgh computing cluster Eddie3. Readpb, bpcomp, tracecomp tools in PhyloBayes and Tracer software were then used to analyse runs. Posterior consensus trees were generated for each run and were reproducible across the eight different runs. The trace plots for independent runs were also analysed to assess for apparent stationarity aiming for an ESS of at least 100. Maxdiff was observed to be < 0.1 (maxdiff= 0.06209, meandiff= 0.00330).

Tree topologies were congruent across the different methods with tree files provided in full (Figure 1b, Supplementary Files 4 and 5). Topology testing of parameter rich models and maximum likelihood constraint trees was performed using IQTree version 1.6.12 and results are provided in Figure 1b. Additional topology testing was performed in PAUP*4.0a163, where 100 random trees were generated along with the maximum likelihood constrained tree with fungal and metazoan sequences constrained to be monophyletic. The SH-test, approximately unbiased (AU) test and expected likelihood weight (ELW) tests were performed and all other alternative trees, including the constraint tree were rejected (p<0.001) (Shimodaira and Hasegawa 2001; Shimodaira 2002; Strimmer and Rambaut 2002; Susko 2014).

***Phylogenetic analysis excluding synapomorphic regions:*** Phylogenetic analyses from Figure 1 were repeated using an alignment with the PAD_N domain removed and with an alignment in which both the PAD_N domain and regions of synapomorphy (Figure S4) were removed. Maximum likelihood analysis using IQTree with ModelFinder using the same best performing fixed empirical rate matrix (WAG+R5+FO) as above (Nguyen et al. 2015; Trifinopoulos et al. 2016; Kalyaanamoorthy et al. 2017; Hoang et al. 2018). Topologies were congruent with analysis of the whole alignment and node support values (Ultrafast Bootstrap 2) for the clades labelled in Figure 1 are provided in Figure S4b.

***PADI Domain annotation***

To identify putative locations for the three PAD domains within PADI homologue sequences from bacteria and fungi, each target PADI sequence was aligned to five metazoan sequences using TCoffee (Di Tommaso et al. 2011). Putative domain sequence regions were then used as a target query for HMMER or HHPred searches (Söding et al. 2005; Finn et al. 2015). HMMER searches were made against the *UniProtKB* database and HHPred searches were performed, firstly against a database of HMM profiles of protein domains in the Protein Data Bank (PDB_mmCIF_4_Aug) and secondly, against a database of profiles from Pfam (Pfam-A_v31.0) (Alva et al. 2016). Once individual sequences were identified as possessing a specific domain architecture, multiple sequence alignments of groups of sequences with common putative domain architecture were made and these were used as queries for each type of search.

For the reported E-values in Figure S4a the following method was used. All sequences from the highlighted clade in the phylogenetic tree were aligned using TCoffee. PAD_C, PAD_M and PAD_N domains from the cyanobacterial sequences, and secondly PAD_C and PAD_M domains from the clade containing a mixture of bacterial and fungal sequences were extracted. These alignments were used as a seed for searches with HHPred against a database of profiles made of the entire human proteome, and against a database of profiles of Pfam domains (Pfam-A_v31.0). HHPred searches were performed using the MPI Bioinformatics Toolkit of the Max Planck Institute for Developmental Biology, Tübingen, Germany (Alva et al. 2016; Zimmermann et al. 2018).

***Multiple sequence alignment of PAD_N domain***

Amino acid sequences were aligned using the TCoffee algorithm (Edgar 2004; Di Tommaso et al. 2011) and visualised using Jalview (Waterhouse et al. 2009). Putative PAD_N domains from the *SPM*/*NX* clade cyanobacterial PADI sequences were identified using HHPred as showing significant statistical evidence for affinity (E-value: 2.5x10^-5^) (Alva et al. 2016; Zimmermann et al. 2018). These were aligned with the PAD_N domain from human PADI paralogues and *Rhincodon typus* (whale shark). The alignment was presented with the program Belvu using a colouring scheme indicating the average BLOSUM62 scores (which are correlated with amino acid conservation) of each alignment column (Henikoff and Henikoff 1992), as represented in Figure 2a. PsiPred (Jones 1999) was used to predict secondary structure for the cyanobacterial PAD_N domains (beta sheets) and presented with the alignment. The experimental secondary structure of the PAD_N domain of human PADI2 was identified from the crystal structure (PDB: 4n2a) (Slade et al. 2015).

***Synapomorphy analysis of PADI calcium binding sites***

Representative fungal, actinobacterial, cyanobacterial, and metazoan PADI sequences were analysed for the conservation of all of the calcium-binding sites (a minimum of three residues coordinate each calcium binding site) and for other critical residues contained at the active site (Figure 2). PADIs from the following species were used: 1) metazoan PADIs from *Homo sapiens*, *Xenopus laevis*, *Oncorhynchus mykiss*, *Callorhinchus milii*, *Branchiostoma floridae*, *Priapulus caudatus*; 2) cyanobacterial PADIs from *Cyanothece sp. 8801, Stanieria cyanosphaera, Chlorogloeopsis fritschii PCC 6912, Crocosphaera subtropica, Aphanothece sacrum, Cyanothece sp. 7424;* 3) fungal PADIs from *Fusarium sp. FOSC 3-a, Periconia macrospinosa, Paracoccidioides lutzii, Blastomyces parvus, Ajellomyces capsulatus, Emmonsia crescens* and; 4) actinobacterial PADIs from *Streptomyces silvensis, Alteromonas lipolytica, Streptomyces sp. 3214.6, Erythrobacter xanthus, Kibdelosporangium aridum, Nocardia brasiliensis ATCC 700358*. Sequences were aligned using MAFFT L-ins-I and compared to functionally annotated regions from *Slade et al*. 2015 and from crystal structures (Arita et al. 2004; Slade et al. 2015; Katoh et al. 2018).

***Accumulated genetic divergence analysis relative to other proteins***

Bitscore density is calculated by taking the bitscore of a query sequence to the target sequence produced by HMMER and dividing by the bitscore of the query sequence to itself (longer sequences have higher bitscores), which gives a value between 0 and 1 (Finn et al. 2015). The bitscore densities of the similarity of 1) the cyanobacterial homologue to the human sequence: ∆bitscoreD_Cy-Hu_ (AC+AH) and 2) of the branchiostomal homologue to the human sequence: ∆bitscoreD_Br-Hu_ (XB+XH) were both calculated (Figure S7). A measure of the total accumulated genetic divergence between late-diverging cyanobacteria (*Cyanothece spp.*) and the last common ancestor of *Branchiostoma spp.* and *Homo sapiens* was then calculated by subtracting ∆bitscoreD_Br-Hu_ from the ∆bitscoreD_Cy-Hu_. This accumulated genetic divergence (AGD) value was calculated for: (1) 26 ribosomal proteins (uS2, uS3, uS4, uS5, uS7, uS8, uS9, uS10, uS11, uS12, uS13, uS17, uS19, uL1, uL2, uL3, uL4, uL5, uL6, uL11, uL13, uL14, uL15, uL22, uL23, uL24), (2) 19 sequences whose proteins are mitochondrially located so are reasonable EGT candidates from the mitochondrion (OTC, ASS1, ARLY, CPS1, PGK, ENO, GAPDH, PK, NAXE, G6PD, RPIA, FUMH, SDHB, SDHA, CS, MDHM, DLAT, DLDH, ACLY) (Timmis et al. 2004), and (3) all 10 proteins still encoded in the mitochondrial genome (MT-ATP6, MT-CO1, MT-CO2, MT-CO3, MT-CYB, MT-1, MT-2, MT-3, MT-4, MT-5). It is notable that by definition, only very highly conserved proteins have an AGD that can be calculated in this extreme example between the last common ancestor of late diverging cyanobacteria and humans: if a protein has diverged substantially then the similarity of the human homologue to the cyanobacterial will not be discernible and no bitscore can be calculated. AGD values of proteins in each category were tested for deviation from a normal distribution using the Shapiro Wilk test (*W* = *b^2^* ⁄ *SS*) (Shapiro and Wilk 1965). Where the calculated p-value exceeded 0.05, the null hypothesis was retained, and the data treated as being normally distributed. Kurtosis and skew were also within the range of the normal distribution. The AGD for PADI proteins (AGD_PADI proteins_ = 0.07) was then compared to the mean AGD of each category of control proteins (e.g. AGD_ribosomal proteins_ = 0.70) and the z-scores were calculated and are presented as p-values.

To compare the extent of divergence relative to ribosomal RNA (rRNA), nucleotide sequences for rRNA were obtained from the SILVA database (Yilmaz et al. 2014). Nucleotide sequences for PADIs were obtained from NCBI and exons extracted. Comparisons were made with EMBOSS Needle using the Needleman-Wunsch global alignment algorithm (Needleman and Wunsch 1970) (gap open: 10, gap extend: 0.5).

***Sequence divergence analyses***

From the AGD analysis performed above, the median EGT candidate protein was selected as a control (ENO). Sequence divergence analysis was performed on ENO and PADI DNA sequences. BEAST v2.4.8 was used to produce a time tree of the clade of subsampled metazoan PADIs and the full clade of closest *SPM*/*NX* cyanobacteria contained within the PATRIC database using the GTR model with 4 gamma distributed rate categories (Drummond and Rambaut 2007; Bouckaert et al. 2014; Uyeda et al. 2016). ENO sequences from the same species under the same model specifications as PADIs were used for the control analysis. The following metazoan species were used: *Homo sapiens* (HS), *Mus musculus* (MM), *Alligator mississippiensis* (AM), *Chelonia mydas* (CM), *Gallus gallus* (GG), *Xenopus laevis* (XL), *Oncorhynchus mykiss* (OM), *Callorhinchus milii* (CM), *Branchiostoma floridae* (BF), *Priapulus caudatus* (PC). To calibrate nodes on the tree, node times were set as the following normally distributed priors: mean 797.0, sigma 72.5 (clade of HS, MM, AM, CM, GG, XL, OM, CM, BF, PC); mean 692.5, sigma 57.5 (clade of HS, MM, AM, CM, GG, XL, OM, CM, BF); mean 473.5, sigma 14.0 (clade of HS, MM, AM, CM, GG, XL, OM, CM); mean 435.0, sigma 6.5 (clade of HS, MM, AM, CM, GG, XL, OM); mean 311.0, sigma 7.5 (clade of HS, MM, AM, CM, GG); mean 89.5, sigma 3.0 (clade of HS, MM). DNA sequences were translated in silico and sequence before the start codon and after the stop codon was removed. DNA sequences were aligned using MAFFT L-ins-I and singly aligning columns were removed. Metazoan divergence times from the fossil record were obtained from timetree.org with bounds on the distributions chosen to span the range of times reported in the literature centered on the median value (Kumar and Hedges 2011). The calibrated Yule model was used as the tree prior. XML files were generated in BEAUti and the MCMC analysis was run using BEAST2 on the CIPRES Gateway on XSEDE. An initial MCMC run of 5,000,000 chains was run for each clock model (Drummond et al. 2006; Drummond and Suchard 2010). Then analysis was performed with two independent runs of 10,000,000 chains under two different clock models– the strict clock model and the relaxed uncorrelated lognormal (UCLN) clock model. The UCLN model relaxes the strict clock by allowing rate heterogeneity across branches: each branch is assumed to have its own rate that is drawn from a shared parametric rate distribution (the log-normal distribution). The different analyses were additionally run under the tree prior (i.e. in the absence of sequence data). Analysis of parameters was performed in Tracer to assess apparent stationarity for the different tree parameters and for acceptable ESS values and congruence was assessed across the independent runs. The predicted divergence time of the metazoan and cyanobacterial clades was given by the marginal posterior distribution of the age of the root of the whole tree. This is given by the TreeHeight parameter. This data was plotted with the kernel density estimate against the TreeHeight parameter for the different runs and summarised in a box and whisker plot. Summary data for the TreeHeight parameter is provided in Figure 3E and includes the highest posterior density 95% credible interval.

In calculating AGD for each mitochondrially encoded protein as compared to its own closest bacterial homologue (as opposed to the homologue from *Cyanothece sp 8801*), we tested for normality using the Shapiro Wilk test (p = 0.109), retaining the null hypothesis that the points are normally distributed. We then calculate the z-statistic for the PADI AGD to its nearest homologue (z= -2.439). This corresponds to a p value = 0.0073. With a p value of 0.0073, we therefore reject the null hypothesis that PADIs fall within the normal distribution of AGD values calculated for mitochondrially encoded proteins relative to their closest bacterial homologue.

***Preparation of recombinant proteins***

PADI gene sequences were obtained from NCBI and synthesised by Thermo GeneArt with flanking EcoRI (at the 5’ end) and XhoI (at the 3’ end) restriction sites. *Cyanothece sp. 8801* PADI and human PADI4 sequences were subcloned into a modified pGEX vector, which included an additional 10X His tag immediately N-terminal of the enzyme sequence (generous gift from Dr Martin Reijns, MRC Human Genetics Unit), by InFusion cloning. GST-His-PADI4 and GST-His-cyanoPADI were expressed in BL21 (DE3) in 2TY cultures. Cells were grown (37 °C; 180 rpm) to an OD_600_ of 0.6 and induced overnight at 18 ̊C with 0.5 mM β-D-1-thiogalactopyranoside (IPTG). Bacterial pellets were harvested by centrifugation (8,000 x *g*; 10 min) and frozen at -80 ̊C. Cell pellets were resuspended in 50 mM Tris pH 7.5, 500 mM NaCl, 20 mM imidazole, 5% Glycerol, 1 mM DTT (1 g dry cell mass in 4 mL), 1X EDTA-free protease inhibitors (Roche), 5 mM MgCl_2_ and 10 units benzonase at 4 ̊C with stirring. Cells were lysed on ice by sonication (7 x 45 sec, with 45 sec breaks) and the lysate was cleared by centrifugation (20,000 x *g*; 20 min). Supernatant was sterile filtered (0.2 μm) before loading by Superloop onto a 5 mL HisTrap column, which was pre-equilibrated with binding buffer. Proteins were purified using an AKTA FPLC system (GE Healthcare). The column was washed with 50 mM Tris pH 7.5, 500 mM NaCl, 25 mM imidazole, 5% Glycerol, 1 mM DTT and the recombinant proteins were eluted with 50 mM TRIS pH 7.5, 500 mM NaCl, 250 mM imidazole, 5% Glycerol, 1 mM DTT. The purified sample was concentrated using Vivaspin MWCO filters into 50 mM HEPES pH 7.5, 150 mM NaCl, 5 mM DTT, 5% (v/v) glycerol, and the concentration determined using Nanodrop.

***Citrullination activity assays***

***Using mouse cell lysates:*** E14 mouse embryonic stem (ES) cells were cultured in GMEM supplemented with 10% fetal calf serum (FCS), 0.1 mM non-essential amino acids, 2 mM L- glutamine, 1 mM sodium pyruvate, 0.1 mM beta-mercaptoethanol and 10^6^ units/L leukaemia inhibitory factor (LIF) (ESGRO, Millipore) and grown on a six well plate until 70% confluent. Cells were harvested in 0.5% NP-40, 20 mM Tris pH 7.6, 1X EDTA-free protease inhibitors, 5 mM DTT, after two washes in PBS (one in PBS containing 2 mM EDTA and one in plain PBS). To shear chromatin and clarify lysates, benzonase and 2 mM MgCl_2_ were added and samples were rotated at 4 ̊C for 30 mins, sheared by passing through a 25G needle and centrifuged at 13000 rpm for 5 mins. Citrullination activity assays were performed with 500 nM recombinant enzyme in 50 mM HEPES pH 7.5, 150 mM NaCl, 5 mM DTT, 5% (v/v) glycerol, either in the presence of 5mM CaCl_2_ or water. Reactions were incubated for 30 mins at 37 ̊C and quenched by boiling at 95 ̊C for 5 mins. Samples were stored at -80 ̊C before immunoblotting.

***Using recombinant histone H3 substrate:*** Reactions were performed in 50 mM HEPES, 137 mM NaCl, 5 mM DTT with 1.5 μM recombinant H3 (New England Biolabs), vehicle or 500 nM recombinant enzyme, and in the presence of either 5 mM CaCl_2_ or water. Reactions incubated for 30 mins at 15 ̊C or 37 ̊C and quenched by boiling at 95 ̊C for 5 mins before immunoblotting.

***Immunoblotting***

Proteins were separated by SDS-PAGE and transferred to nitrocellulose membrane using wet transfer. Membranes were blocked in 5% BSA in TBS containing 0.1% Tween-20 for 1 h at room temperature. Proteins were detected using primary antibodies against anti-H3 (Abcam ab10799, 1:1000), anti-H3CitR2 (Abcam ab176843, 1:2000), anti-NPM1 (Abcam ab37659, 1:200) and anti-GST (Abcam ab19256, 1:1000) overnight at 4 ̊C and in secondary antibody at 1:5000 for 1 h at room temperature. Membranes were incubated in Pierce ECL reagent and imaged using ImageQuant LAS 4000 (GE). Citrulline-containing proteins were modified on the membrane and detected using the anti-modified citrulline detection kit (Millipore, 17-347) according to manufacturer’s instructions.

**References**

Altschul SF, Gish W, Miller W, Myers EW, Lipman DJ. 1990. Basic local alignment search tool. J Mol Biol. doi:10.1016/S0022-2836(05)80360-2.

Altschul SF, Madden TL, Schäffer AA, Zhang J, Zhang Z, Miller W, Lipman DJ. 1997. Gapped BLAST and PSI-BLAST: A new generation of protein database search programs. Nucleic Acids Res. doi:10.1093/nar/25.17.3389.

Alva V, Nam SZ, Söding J, Lupas AN. 2016. The MPI bioinformatics Toolkit as an integrative platform for advanced protein sequence and structure analysis. Nucleic Acids Res. doi:10.1093/nar/gkw348.

Anisimova M, Gil M, Dufayard JF, Dessimoz C, Gascuel O. 2011. Survey of branch support methods demonstrates accuracy, power, and robustness of fast likelihood-based approximation schemes. Syst Biol. doi:10.1093/sysbio/syr041.

Arita K, Hashimoto H, Shimizu T, Nakashima K, Yamada M, Sato M. 2004. Structural basis for Ca2+-induced activation of human PAD4. Nat Struct Mol Biol. 11(8):777–783. doi:10.1038/nsmb799.

Bernard G, Greenfield P, Ragan MA, Chan CX. 2018. k -mer Similarity, Networks of Microbial Genomes, and Taxonomic Rank . mSystems. doi:10.1128/msystems.00257-18.

Bouckaert R, Heled J, Kühnert D, Vaughan T, Wu CH, Xie D, Suchard MA, Rambaut A, Drummond AJ. 2014. BEAST 2: A Software Platform for Bayesian Evolutionary Analysis. PLoS Comput Biol. doi:10.1371/journal.pcbi.1003537.

Capella-Gutiérrez S, Silla-Martínez JM, Gabaldón T. 2009. trimAl: A tool for automated alignment trimming in large-scale phylogenetic analyses. Bioinformatics. doi:10.1093/bioinformatics/btp348.

Chen C, Natale DA, Finn RD, Huang H, Zhang J, Wu CH, Mazumder R. 2011. Representative Proteomesz: A Stable, Scalable and Unbiased proteome set for sequence analysis and functional annotation. PLoS One. doi:10.1371/journal.pone.0018910.

Crotty SM, Minh BQ, Bean NG, Holland BR, Tuke J, Jermiin LS, Haeseler A Von. 2020. GHOST: Recovering Historical Signal from Heterotachously Evolved Sequence Alignments. Syst Biol. doi:10.1093/sysbio/syz051.

Drummond AJ, Ho SYW, Phillips MJ, Rambaut A. 2006. Relaxed phylogenetics and dating with confidence. PLoS Biol. doi:10.1371/journal.pbio.0040088.

Drummond AJ, Rambaut A. 2007. BEAST: Bayesian evolutionary analysis by sampling trees. BMC Evol Biol. doi:10.1186/1471-2148-7-214.

Drummond AJ, Suchard MA. 2010. Bayesian random local clocks, or one rate to rule them all. BMC Biol. doi:10.1186/1741-7007-8-114.

Eddy SR. 2011. Accelerated profile HMM searches. PLoS Comput Biol. doi:10.1371/journal.pcbi.1002195.

Edgar RC. 2004. MUSCLE: Multiple sequence alignment with high accuracy and high throughput. Nucleic Acids Res. doi:10.1093/nar/gkh340.

Finn RD, Clements J, Arndt W, Miller BL, Wheeler TJ, Schreiber F, Bateman A, Eddy SR. 2015. HMMER web server: 2015 Update. Nucleic Acids Res. doi:10.1093/nar/gkv397.

Guindon S, Dufayard JF, Lefort V, Anisimova M, Hordijk W, Gascuel O. 2010. New algorithms and methods to estimate maximum-likelihood phylogenies: Assessing the performance of PhyML 3.0. Syst Biol. doi:10.1093/sysbio/syq010.

György B, Tóth E, Tarcsa E, Falus A, Buzás EI. 2006. Citrullination: A posttranslational modification in health and disease. Int J Biochem Cell Biol. doi:10.1016/j.biocel.2006.03.008.

Henikoff S, Henikoff JG. 1992. Amino acid substitution matrices from protein blocks. Proc Natl Acad Sci U S A. doi:10.1073/pnas.89.22.10915.

Hildebrand A, Remmert M, Biegert A, Söding J. 2009. Fast and accurate automatic structure prediction with HHpred. Proteins Struct Funct Bioinforma. doi:10.1002/prot.22499.

Hoang DT, Chernomor O, Von Haeseler A, Minh BQ, Vinh LS. 2018. UFBoot2: Improving the ultrafast bootstrap approximation. Mol Biol Evol. doi:10.1093/molbev/msx281.

Holm L, Rosenström P. 2010. Dali server: Conservation mapping in 3D. Nucleic Acids Res. doi:10.1093/nar/gkq366.

Huerta-Cepas J, Serra F, Bork P. 2016. ETE 3: Reconstruction, Analysis, and Visualization of Phylogenomic Data. Mol Biol Evol. doi:10.1093/molbev/msw046.

Huerta-Cepas J, Szklarczyk D, Forslund K, Cook H, Heller D, Walter MC, Rattei T, Mende DR, Sunagawa S, Kuhn M, et al. 2016. EGGNOG 4.5: A hierarchical orthology framework with improved functional annotations for eukaryotic, prokaryotic and viral sequences. Nucleic Acids Res. doi:10.1093/nar/gkv1248.

Jones DT. 1999. Protein secondary structure prediction based on position-specific scoring matrices. J Mol Biol. doi:10.1006/jmbi.1999.3091.

Kalyaanamoorthy S, Minh BQ, Wong TKF, Von Haeseler A, Jermiin LS. 2017. ModelFinder: Fast model selection for accurate phylogenetic estimates. Nat Methods. doi:10.1038/nmeth.4285.

Katoh K, Rozewicki J, Yamada KD. 2018. MAFFT online service: Multiple sequence alignment, interactive sequence choice and visualization. Brief Bioinform. doi:10.1093/bib/bbx108.

Kumar S, Hedges SB. 2011. Timetree2: Species divergence times on the iPhone. Bioinformatics. doi:10.1093/bioinformatics/btr315.

Lartillot N, Philippe H. 2004. A Bayesian mixture model for across-site heterogeneities in the amino-acid replacement process. Mol Biol Evol. doi:10.1093/molbev/msh112.

Lartillot N, Rodrigue N, Stubbs D, Richer J. 2013. Phylobayes mpi: Phylogenetic reconstruction with infinite mixtures of profiles in a parallel environment. Syst Biol. doi:10.1093/sysbio/syt022.

Le SQ, Gascuel O. 2008. An improved general amino acid replacement matrix. Mol Biol Evol. doi:10.1093/molbev/msn067.

Letunic I, Bork P. 2016. Interactive tree of life (iTOL) v3: an online tool for the display and annotation of phylogenetic and other trees. Nucleic Acids Res. doi:10.1093/nar/gkw290.

McGraw WT, Potempa J, Farley D, Travis J. 1999. Purification, characterization, and sequence analysis of a potential virulence factor from Porphyromonas gingivalis, peptidylarginine deiminase. Infect Immun. doi:10.1128/iai.67.7.3248-3256.1999.

Meng EC, Pettersen EF, Couch GS, Huang CC, Ferrin TE. 2006. Tools for integrated sequence-structure analysis with UCSF Chimera. BMC Bioinformatics. doi:10.1186/1471-2105-7-339.

Needleman SB, Wunsch CD. 1970. A general method applicable to the search for similarities in the amino acid sequence of two proteins. J Mol Biol. doi:10.1016/0022-2836(70)90057-4.

Nguyen LT, Schmidt HA, Von Haeseler A, Minh BQ. 2015. IQ-TREE: A fast and effective stochastic algorithm for estimating maximum-likelihood phylogenies. Mol Biol Evol. doi:10.1093/molbev/msu300.

Pettersen EF, Goddard TD, Huang CC, Couch GS, Greenblatt DM, Meng EC, Ferrin TE. 2004. UCSF Chimera - A visualization system for exploratory research and analysis. J Comput Chem. doi:10.1002/jcc.20084.

Potter SC, Luciani A, Eddy SR, Park Y, Lopez R, Finn RD. 2018. HMMER web server: 2018 update. Nucleic Acids Res. doi:10.1093/nar/gky448.

Quang LS, Gascuel O, Lartillot N. 2008. Empirical profile mixture models for phylogenetic reconstruction. Bioinformatics. doi:10.1093/bioinformatics/btn445.

Rambaut A. 2016. FigTree. version 1.4.3. Inst Evol Biol Univ Edinburgh.

Remmert M, Biegert A, Hauser A, Söding J. 2012. HHblits: Lightning-fast iterative protein sequence searching by HMM-HMM alignment. Nat Methods. doi:10.1038/nmeth.1818.

Ronquist F, Teslenko M, Van Der Mark P, Ayres DL, Darling A, Höhna S, Larget B, Liu L, Suchard MA, Huelsenbeck JP. 2012. Mrbayes 3.2: Efficient bayesian phylogenetic inference and model choice across a large model space. Syst Biol. doi:10.1093/sysbio/sys029.

Sangwan N, Xia F, Gilbert JA. 2016. Recovering complete and draft population genomes from metagenome datasets. Microbiome. doi:10.1186/s40168-016-0154-5.

Shapiro SS, Wilk MB. 1965. An Analysis of Variance Test for Normality (Complete Samples). Biometrika. doi:10.2307/2333709.

Shimodaira H. 2002. An approximately unbiased test of phylogenetic tree selection. Syst Biol. doi:10.1080/10635150290069913.

Shimodaira H, Hasegawa M. 1999. Multiple comparisons of log-likelihoods with applications to phylogenetic inference. Mol Biol Evol. doi:10.1093/oxfordjournals.molbev.a026201.

Shimodaira H, Hasegawa M. 2001. CONSEL: for assessing the confidence of phylogenetic tree selection. Bioinformatics. doi:10.1093/bioinformatics/17.12.1246.

Slade DJ, Fang P, Dreyton CJ, Zhang Y, Fuhrmann J, Rempel D, Bax BD, Coonrod SA, Lewis HD, Guo M, et al. 2015. Protein arginine deiminase 2 binds calcium in an ordered fashion: Implications for inhibitor design. ACS Chem Biol. 10(4):1043–1053. doi:10.1021/cb500933j.

Söding J. 2005. Protein homology detection by HMM-HMM comparison. Bioinformatics. doi:10.1093/bioinformatics/bti125.

Söding J, Biegert A, Lupas AN. 2005. The HHpred interactive server for protein homology detection and structure prediction. Nucleic Acids Res. doi:10.1093/nar/gki408.

Steinegger M, Söding J. 2017. MMseqs2 enables sensitive protein sequence searching for the analysis of massive data sets. Nat Biotechnol. doi:10.1038/nbt.3988.

Strimmer K, Rambaut A. 2002. Inferring confidence sets of possibly misspecified gene trees. Proc R Soc B Biol Sci. doi:10.1098/rspb.2001.1862.

Susko E. 2014. Tests for two trees using likelihood methods. Mol Biol Evol. doi:10.1093/molbev/msu039.

Timmis JN, Ayliff MA, Huang CY, Martin W. 2004. Endosymbiotic gene transfer: Organelle genomes forge eukaryotic chromosomes. Nat Rev Genet. doi:10.1038/nrg1271.

Di Tommaso P, Moretti S, Xenarios I, Orobitg M, Montanyola A, Chang JM, Taly JF, Notredame C. 2011. T-Coffee: A web server for the multiple sequence alignment of protein and RNA sequences using structural information and homology extension. Nucleic Acids Res. doi:10.1093/nar/gkr245.

Touz MC, Rópolo AS, Rivero MR, Vranych CV, Conrad JT, Svard SG, Nash TE. 2008. Arginine deiminase has multiple regulatory roles in the biology of Giardia lamblia. J Cell Sci. doi:10.1242/jcs.026963.

Trifinopoulos J, Nguyen LT, von Haeseler A, Minh BQ. 2016. W-IQ-TREE: a fast online phylogenetic tool for maximum likelihood analysis. Nucleic Acids Res. doi:10.1093/nar/gkw256.

Uyeda JC, Harmon LJ, Blank CE. 2016. A comprehensive study of cyanobacterial morphological and ecological evolutionary dynamics through deep geologic time. PLoS One. doi:10.1371/journal.pone.0162539.

Waterhouse AM, Procter JB, Martin DMA, Clamp M, Barton GJ. 2009. Jalview Version 2-A multiple sequence alignment editor and analysis workbench. Bioinformatics. doi:10.1093/bioinformatics/btp033.

Wattam AR, Davis JJ, Assaf R, Boisvert S, Brettin T, Bun C, Conrad N, Dietrich EM, Disz T, Gabbard JL, et al. 2017. Improvements to PATRIC, the all-bacterial bioinformatics database and analysis resource center. Nucleic Acids Res. doi:10.1093/nar/gkw1017.

Whelan S, Goldman N. 2001. A general empirical model of protein evolution derived from multiple protein families using a maximum-likelihood approach. Mol Biol Evol. doi:10.1093/oxfordjournals.molbev.a003851.

Yilmaz P, Parfrey LW, Yarza P, Gerken J, Pruesse E, Quast C, Schweer T, Peplies J, Ludwig W, Glöckner FO. 2014. The SILVA and “all-species Living Tree Project (LTP)” taxonomic frameworks. Nucleic Acids Res. doi:10.1093/nar/gkt1209.

Zimmermann L, Stephens A, Nam SZ, Rau D, Kübler J, Lozajic M, Gabler F, Söding J, Lupas AN, Alva V. 2018. A Completely Reimplemented MPI Bioinformatics Toolkit with a New HHpred Server at its Core. J Mol Biol. doi:10.1016/j.jmb.2017.12.007.
