# Supplementary material for: Citrullination Was Introduced into Animals by Horizontal Gene Transfer from Cyanobacteria
Source: Mol Biol Evol. 2021 Nov 3;39(2):msab317. doi: 10.1093/molbev/msab317 (PMC8826395; doi:10.1093/molbev/msab317)
Supplement: msab317_Supplementary_Data [file msab317_supplementary_data.zip › Cummings_PADI evolution_SupplementaryFigures_Revised.pdf]

## Figure S1

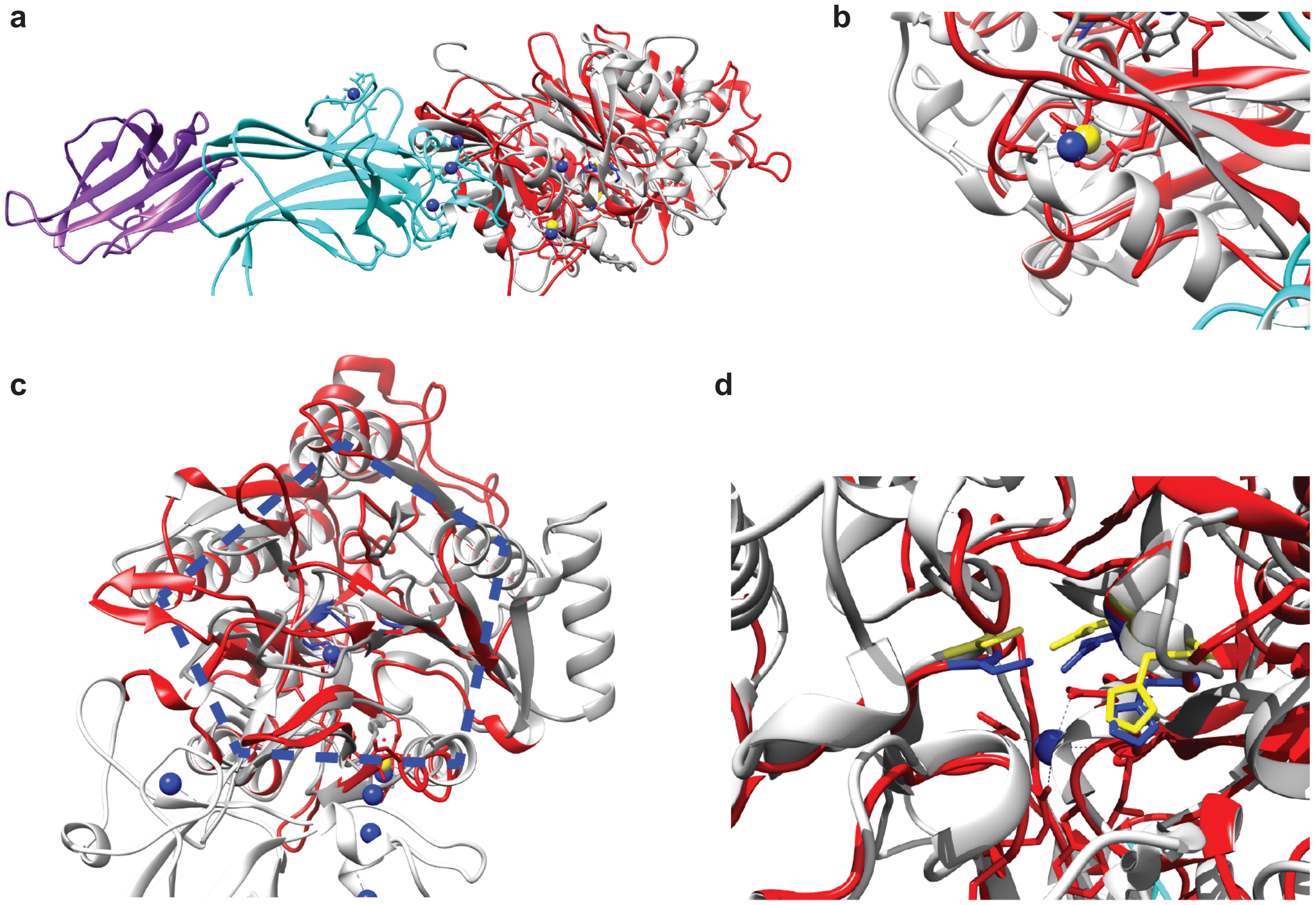


**Figure S1: The active site of human PADI2 adopts an ancient configuration.** PADI2 structure (PDB: 4n2c) in purple (PAD_N domain), cyan (PAD_M domain) and red (PAD_C domain) was superimposed with the structure of Agmatine deiminase from Chlorobium tepidum (ctAgD) in grey (PDB: 1xkn). **a)** Full structure of PADI2 shows that Agmatine deminase (grey) superimposes onto the PAD_C pfam domain (red). **b)** Detail of the conserved metal ion binding site between the two enzymes (Calcium binding site 2 in PADI2, blue sphere, and Na+ site in ctAgD, yellow sphere). **c)** Detail of the PAD_C domain shows the conservation of the overall pentein fold (blue dotted line shows five-fold rotational symmetry). **d)** Active site configuration and positioning is conserved between PADI2 (blue: His-Asp-Cys triad) and ctAgD (yellow: His-Asp-Cys triad).

## Figure S2

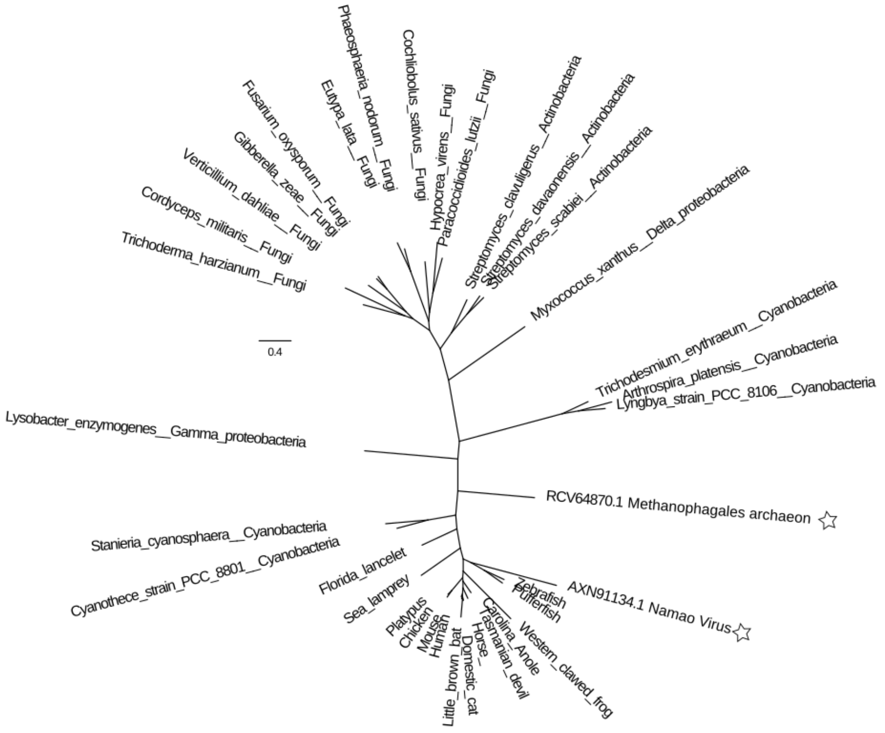


**Figure S2: Phylogeny of two spurious putative *PADI* sequences from unexpected clades within the core set of *PADI* sequences.** The putative *PADI* homologues from *Namao virus* and *Methanophagales archeon* are indicated with a star icon. The phylogeny of *PADI* homologues places the *Namao virus* sequence within the clade containing bony fish. Since this virus is known to infect *Acipenser fulvescens* (lake sturgeon) and the particular genome in question was isolated from an infected tissue sample, it is highly likely that this sequence is the result of sample contamination from the host organism. The *Methanophagales archeon* sequence is placed very close to bacterial sequences at a distance which is inconsistent with even the slow divergence of core housekeeping genes shared between bacteria and archaea. As *PADI* does not appear to be part of the core archaeal genome as it is not a housekeeping gene vital for archaeal cell survival, it is unlikely that this sequence would show such a slow rate of evolution while also being lost simultaneously within all other archaeal species.

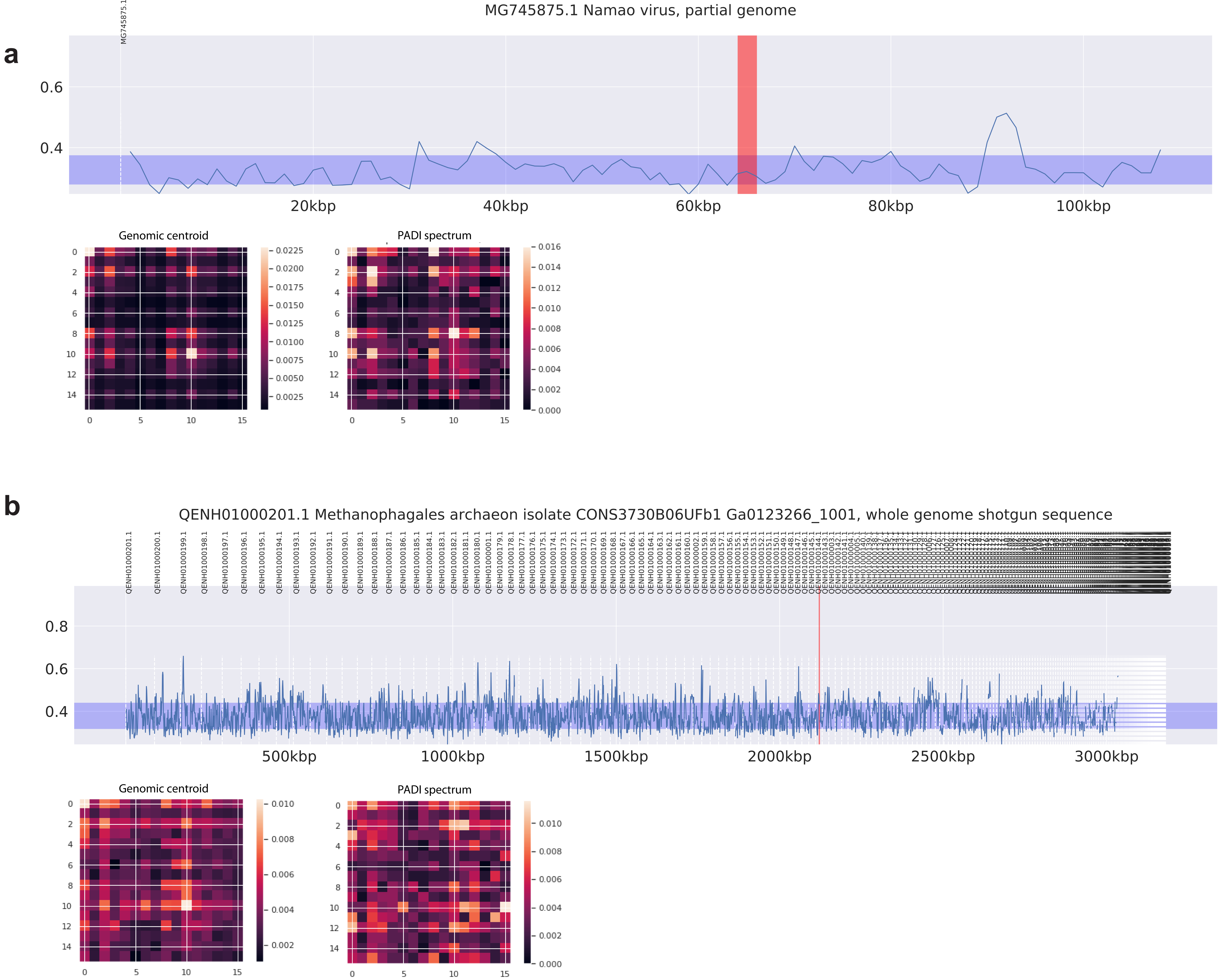

a
MG745875.1 Namao virus, partial genome
MG745875.1
0.6
0.4
20kbp
40kbp
60kbp
80kbp
100kbp
Genomic centroid
PADI spectrum
b
QENH01000201.1 Methanophagales archaeon isolate CONS3730B06UFb1 Ga0123266_1001, whole genome shotgun sequence
0.8
0.6
0.4
500kbp
1000kbp
1500kbp
2000kbp
2500kbp
3000kbp
Genomic centroid
PADI spectrum

**Figure S3:** K-mer spectra analysis of genomic contigs of spurious viral **(a)** and archaeal **(b)** hits. The norm of the vector defined by the difference between the genomic average and the K-mer spectrum of each window is shown over all contigs in both assemblies. Vertical white dashed lines denote the start of a contig. The horizontal blue shaded region shows the standard deviation around the average distance of all windows relative to the genomic average. The red vertical shaded area shows the position of the putative PADI homologue within the genome. Contig identifiers are not shown for the genome of Methanophagales archeon due to the high number of small contigs. The assembly quality of this genome is fairly poor, contigs are ordered by length and do not represent the actual arrangement. K-mer spectra for contigs shorter than 1kbp were not calculated. Neither of the genomes present a region showing anomalous K-mer spectra typical of a continuous horizontally transferred genomic region. This indicates that the two PADI sequences were falsely attributed to these genomes during assembly due to their K-mer spectra being close to the genomic average of these two organisms by chance.

## Figure S4

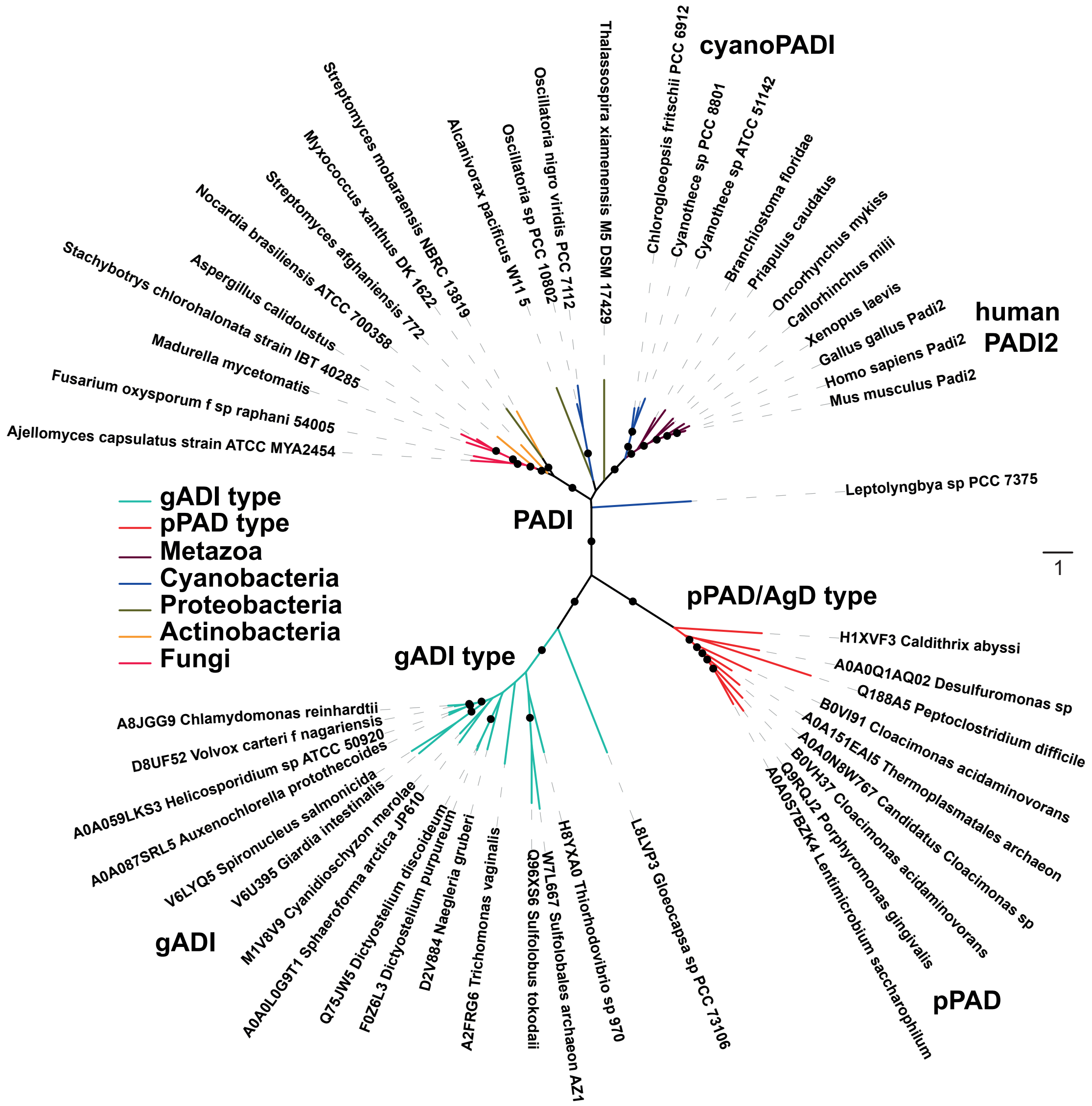


**Figure S4: Metazoan PADI sequences are evolutionarily distinct from other bacterial and eukaryotic citrullinating enzymes.** PADIs form distinct clades to ADIs, AgDs, gADI (arginine deiminase from Giardia lamblia) and pPAD (porphyromonas-type peptidylarginine deiminase from Porphyromonas gingivalis) sequences. The tree is shown unrooted with solid circles indicating consensus node support of >95%.

# Figure S5

**a**

## Bayesian Tree (Mr Bayes) without subsampling

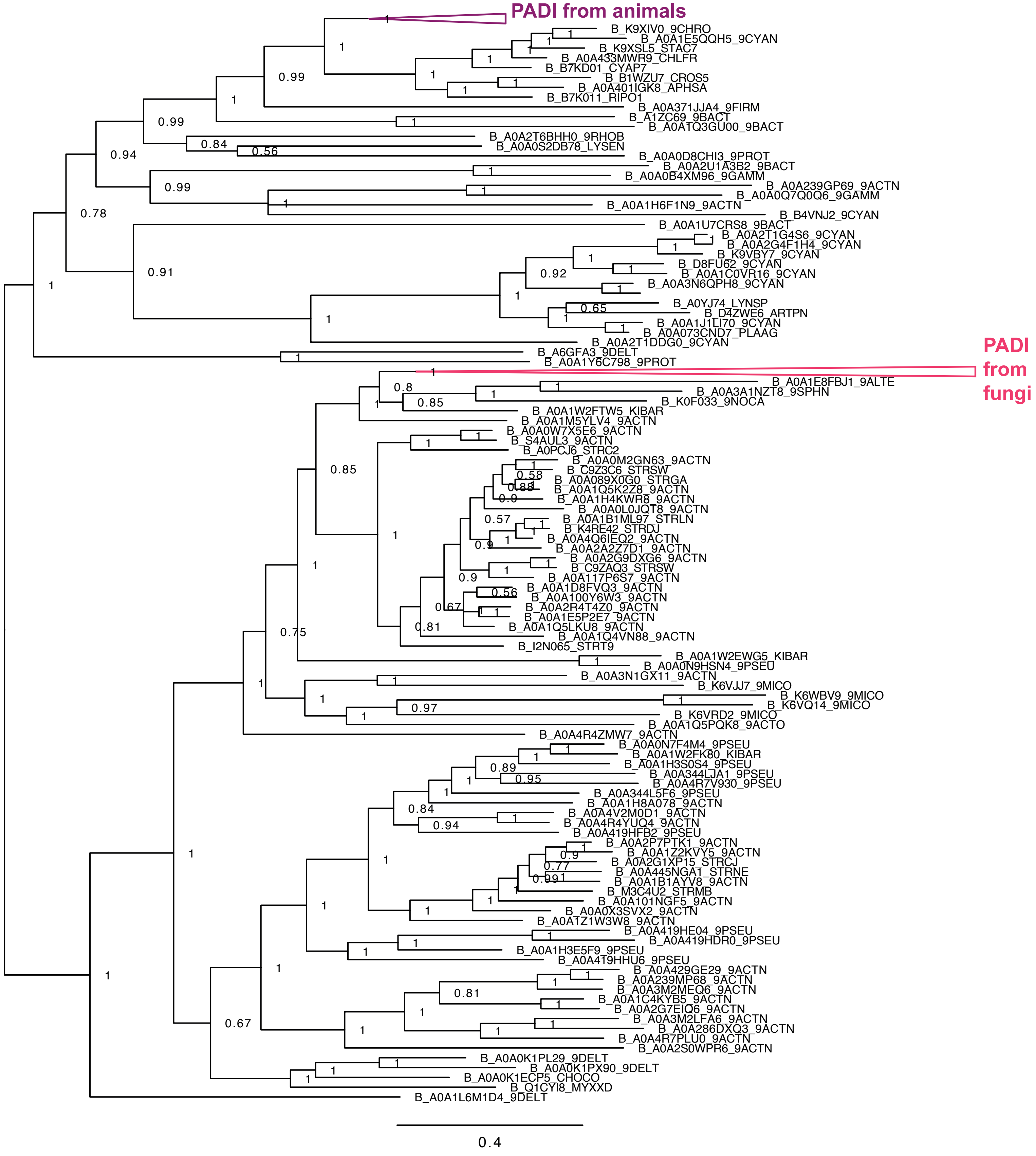

# Figure S5

**b**

## Maximum Likelihood Tree (IQTree) without subsampling

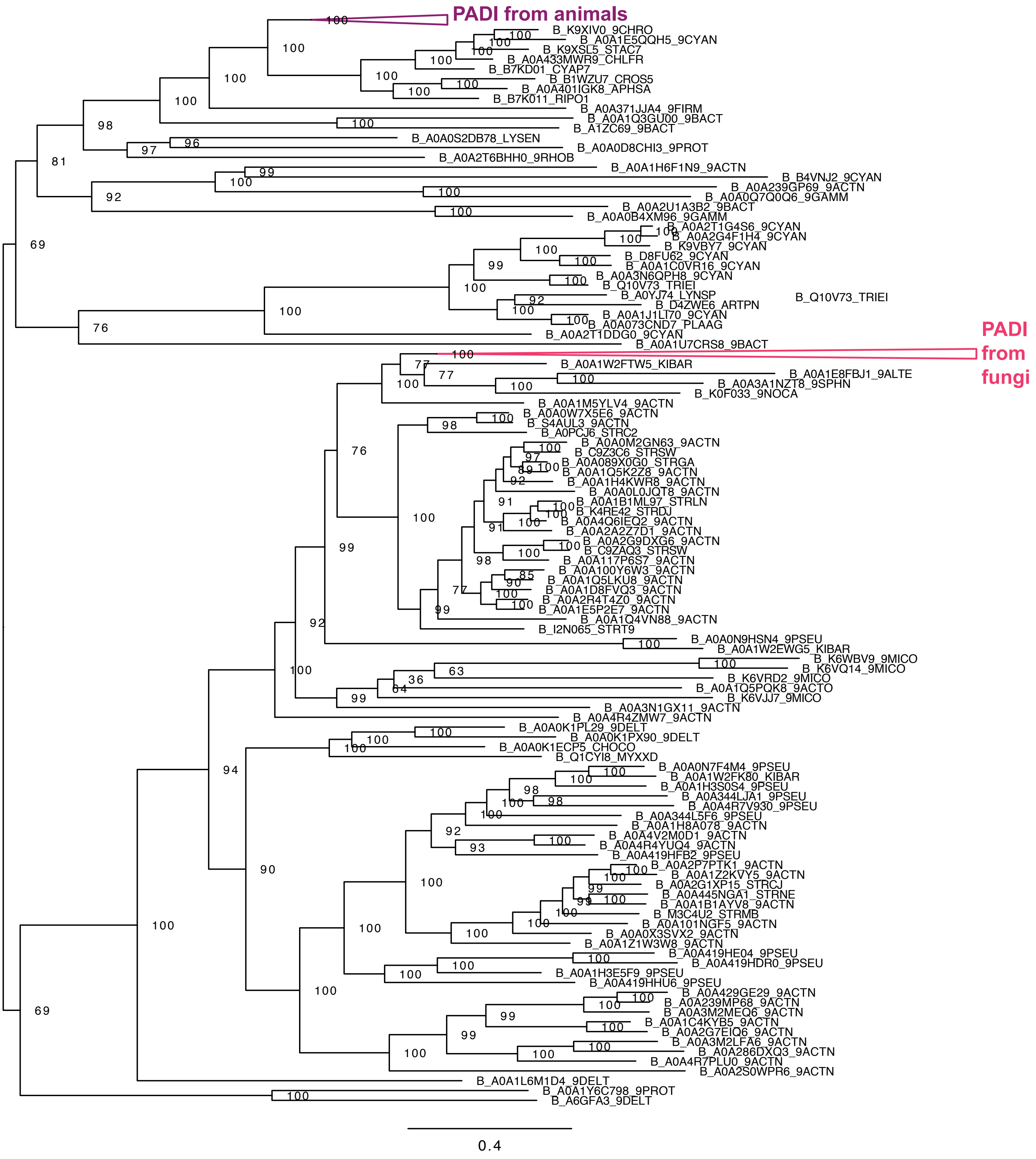

## Figure S5

**c**

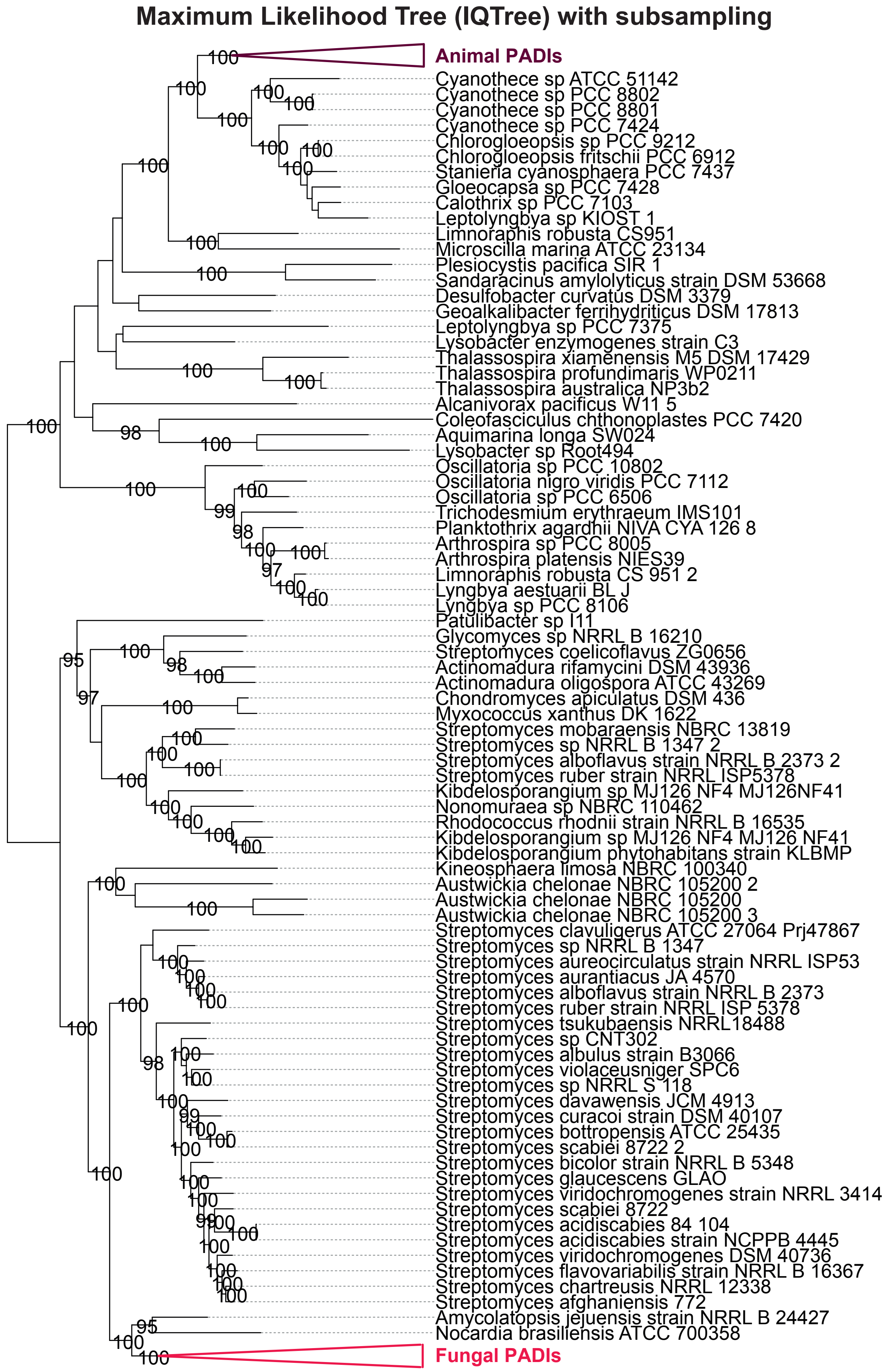


**Figure S5: PADI phylogeny. a, b)** PADI phylogeny without subsampling. All PADI sequences were obtained from UniProtKB rp55 using HMMER. The alignment was made using MAFFT-L-ins-I and phylogenetic analysis performed using MrBayes (a) and IQTree (b,c). Posterior probability (a) or bootstrap values using Ultrafast Bootstrap 2 with 1000 replicates (b,c) respectively are shown on each tree denoting node support. Metazoan sequences were recovered as a single clade; fungal PADI sequences were also recovered as a single clade. All bacterial sequences are shown uncollapsed on the tree. The tree is shown rooted at the midpoint. **c)** PADI phylogeny with subsampling. Phylogenetic analysis of all putative bacterial PADI sequences in the PATRIC database and with representative animal and fungal sequences to obtain 150 sequences. The tree is shown rooted at the midpoint with solid circles indicating consensus node support of >95% and a number of critical nodes corresponding to Figure 1 labelled in full.

# Figure S6

**a**

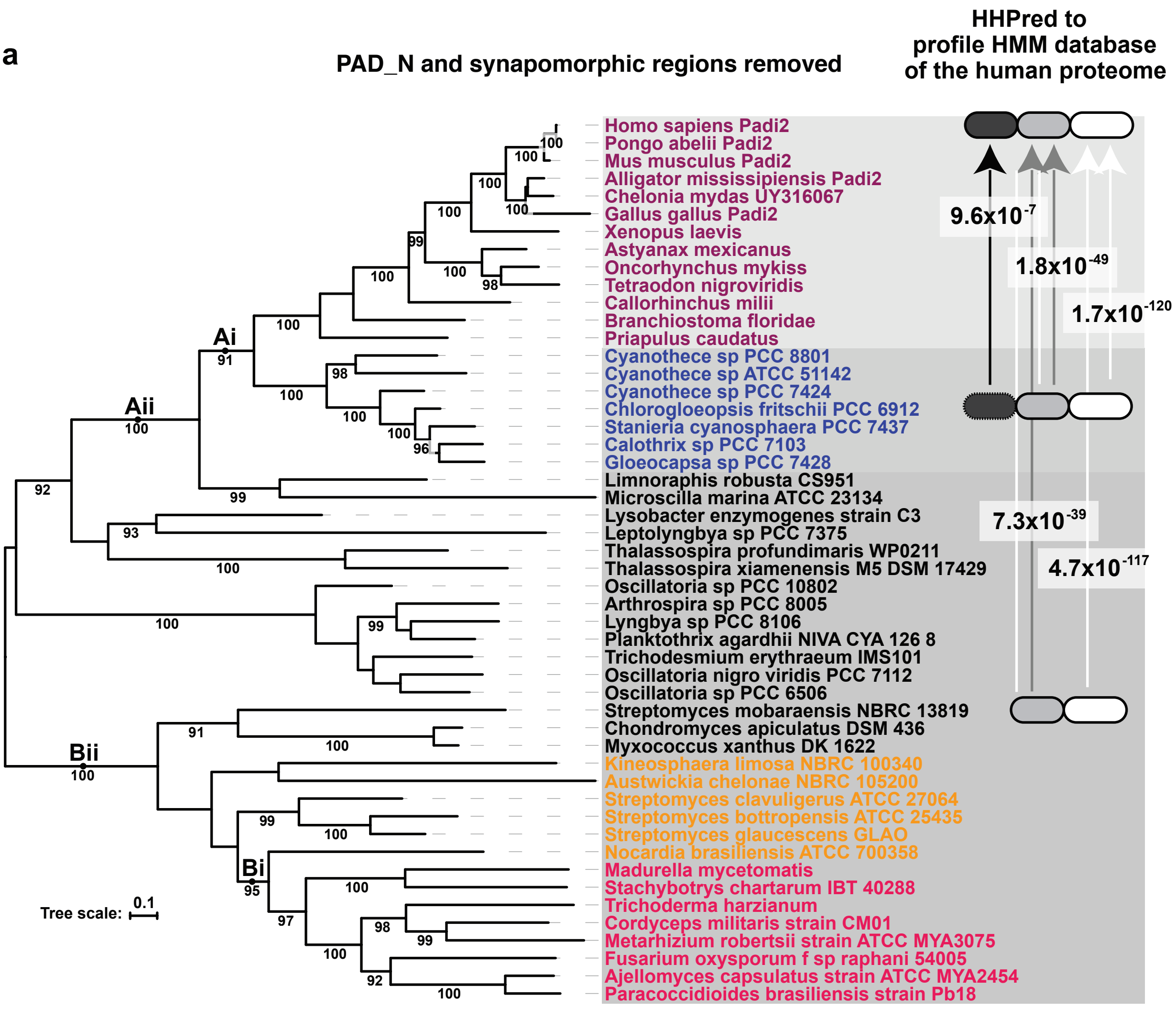


**b**

| Cartoon: | Region of alignment used: | Node support for clade: | | | |
|---|---|---|---|---|---|
| | | Ai | Aii | Bi | Bii |
| | Whole protein | 99 | 100 | 99 | 100 |
| | PAD_N removed | 97 | 100 | 99 | 100 |
| * * | PAD_N and synapomorphy removed | 91 | 100 | 95 | 100 |

**Figure S6: Domain architecture analysis of PADI orthologues. a)** Representation of PADI domain architecture across the phylogenetic tree. Protein regions aligning to the metazoan PADI domains were extracted from sequences represented in the different clades of the tree (firstly of cyanobacterial sequences and secondly of a mixture of bacterial and fungal sequences). An HMM profile was made and HHPred was used to search against a database of profiles made of the entire human proteome, and against a database of Pfam domain profiles. The E-values are given for these searches where significant sequence similarity could be identified from HHPred searches. **b)** Phylogenetic analyses from Figure 1a (top row) were repeated using an alignment where the PAD_N domain (middle row), or both the PAD_N domain and regions of synapomorphy (bottom row) were removed. Maximum likelihood inference using IQTree was used in all three cases and ModelFinder was used to select the best performing fixed empirical rate matrix (WAG + R5 +F0). Node support values correspond to clades annotated in Figure 1a as topologies were congruent.

# Figure S7

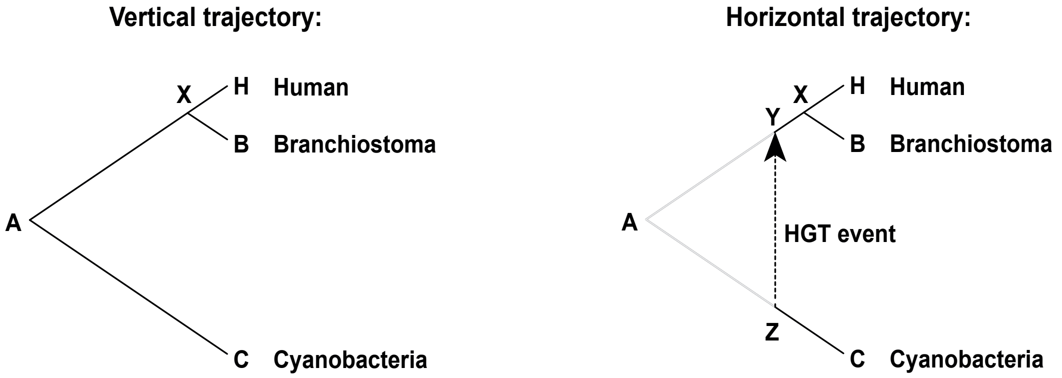


Accumulated genetic divergence =
ΔBitscore(Cy-Hu) - ΔBitscore(Br-Hu)
(AC + AH) - (XB + XH)
= (AC + AX) - XB

If vertical, expect (AC + AX) - XB >> 0

Accumulated genetic divergence =
ΔBitscore(Cy-Hu) - ΔBitscore(Br-Hu)
(AC + AH) - (XB + XH)

If horizontal, AC = ZC and AH = YH
=> (ZC + YH) - (XB + XH)
= (ZC + YX) - XB

If YX ≈ 0, then (ZC + YX) - XB can be ≈ 0

**Figure S7: Calculating the AGD of a given protein between its homologues in *Homo sapiens, Branchiostoma spp. and Cyanothece spp.*** The bitscore density of the similarity of the cyanobacterial homologue to the human sequence $\Delta$bitscore$_{Cy\text{-}Hu}$ (AC+AH) and the bitscore density of the similarity of the branchiostomal homologue to the human sequence $\Delta$bitscore$_{Br\text{-}Hu}$ (XB+XH) are calculated. In a vertical scenario the AGD, given by (AC+AX) – XB, will be much greater than zero. In a horizontal scenario, (ZC+YX) may be approximately equal to XB and so the AGD, given by (ZC + YX) – XB may be close to zero.

## Figure S8a

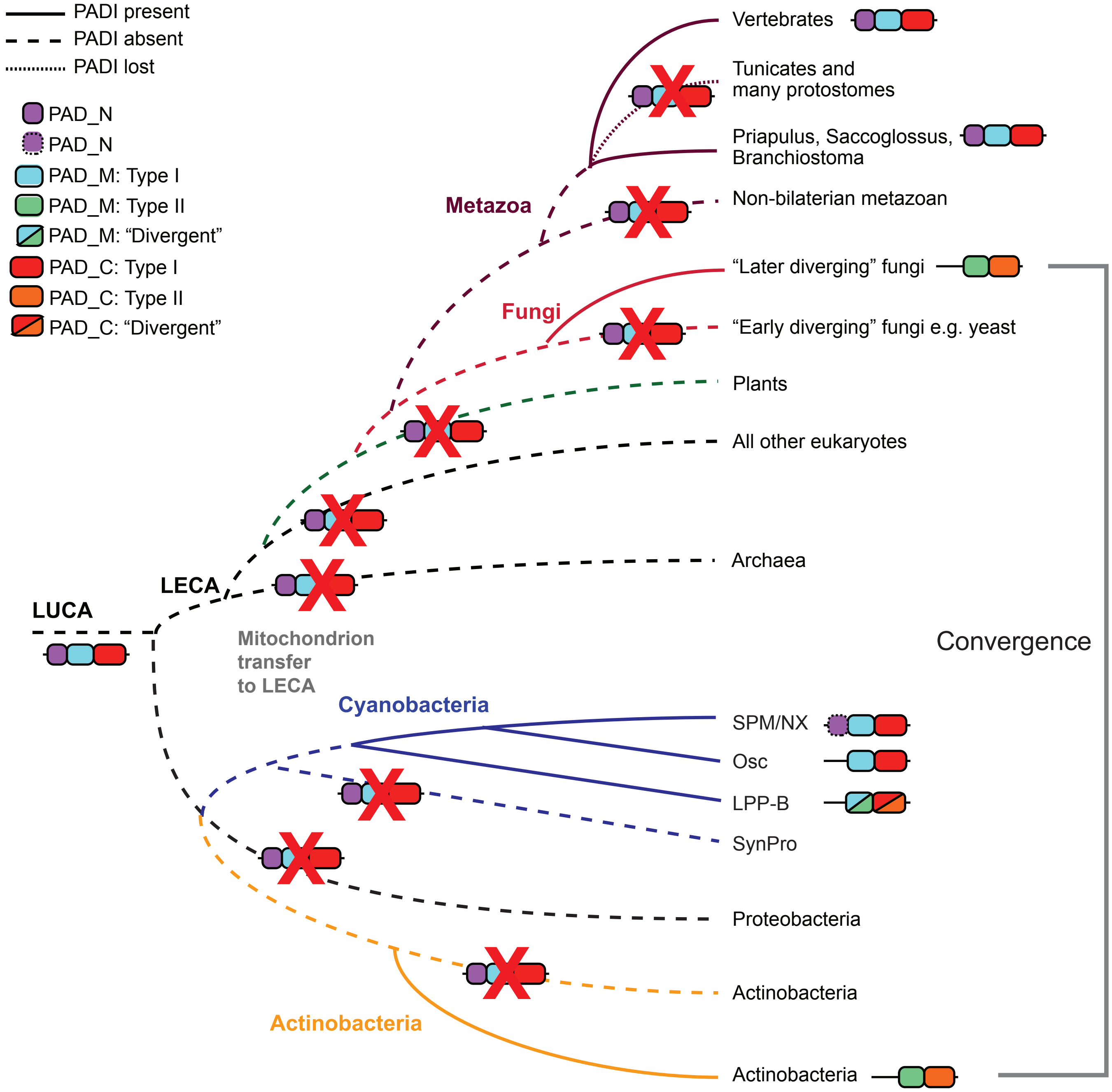


**One PADI in LUCA, many losses and convergence**

## Figure S8b

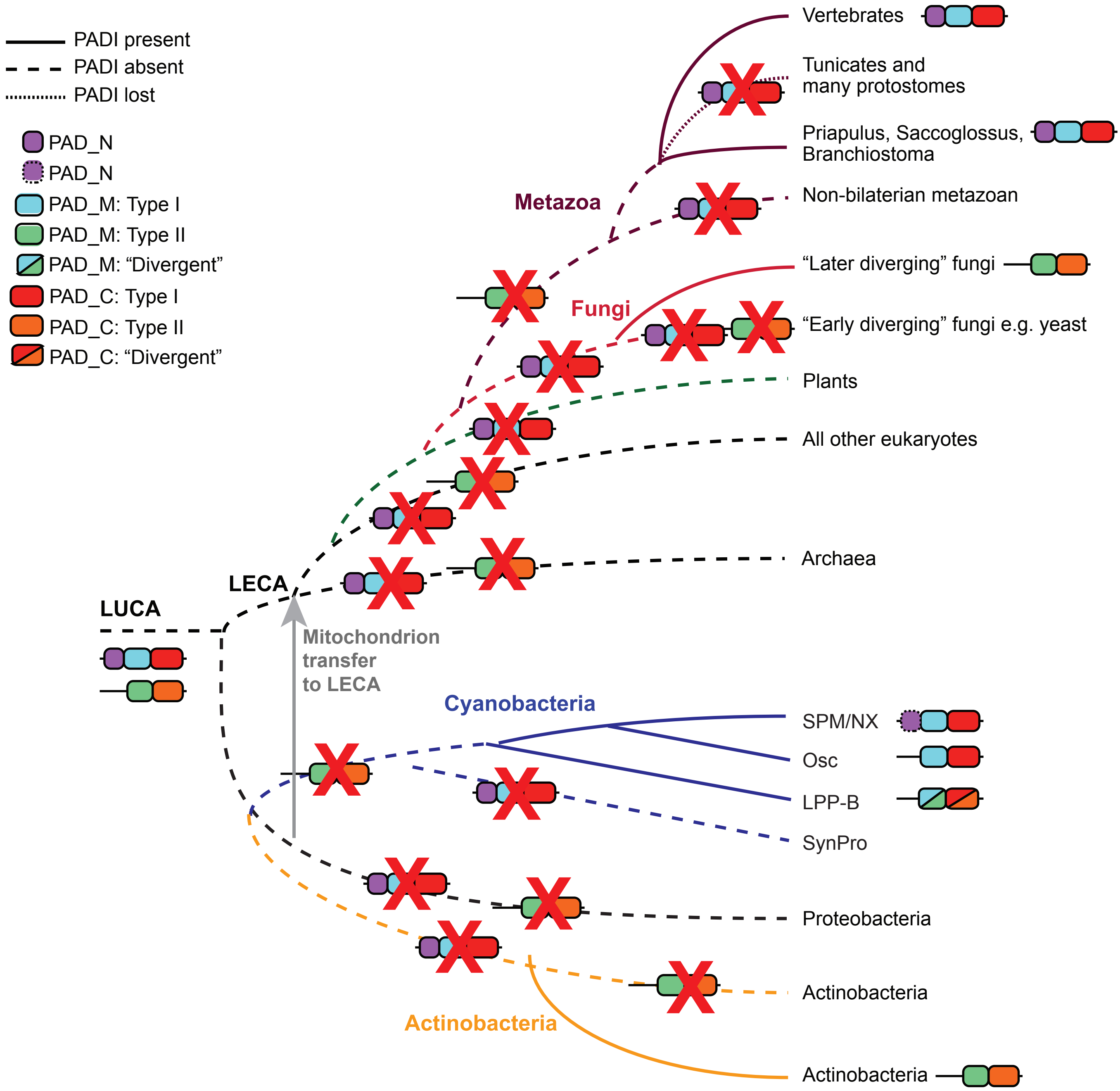


**Two PADIs in LUCA, many losses**

## Figure S8c

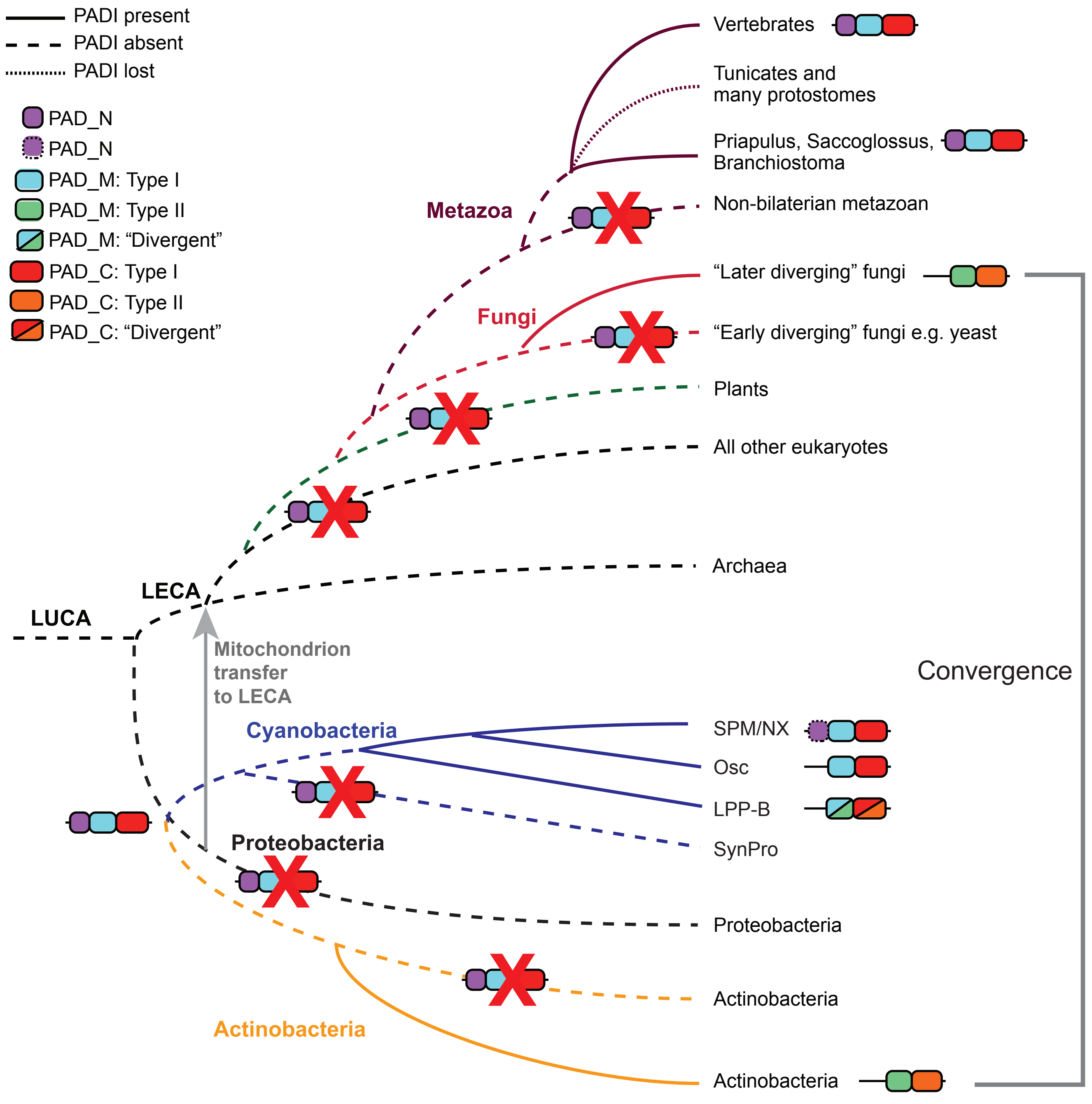


**One PADI transfered by EGT, many losses and convergence**

## Figure S8d

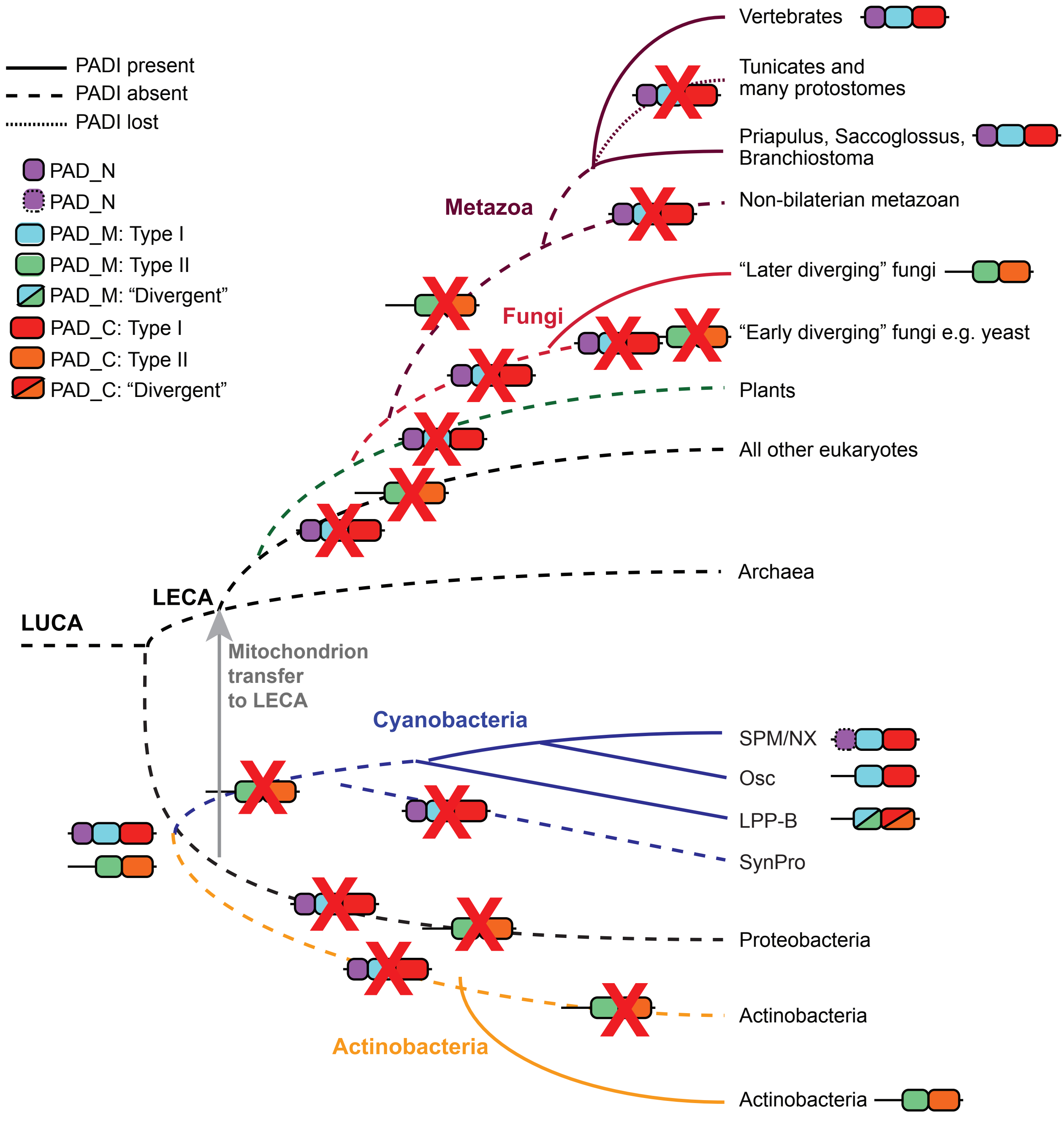


**Two PADIs transfered by EGT, many losses**

**Figure S8: Schematic representation of theoretical scenarios of vertical evolution of the PADI sequence.** Dotted lines indicate lineages where a PADI sequence cannot be observed in modern genomes. Crosses indicate loss of a sequence within a certain lineage. Domain architecture of the PADI sequences is depicted in the schematic legend.

**a) One PADI in the LUCA, many losses and convergence.** If one PADI sequence existed in the LUCA, a vertical evolution scenario requires that (i) this sequence has undergone very little sequence change between cyanobacteria and animals; (ii) despite this degree of conservation, the PADI gene was lost in many independent events across evolution; (iii) more divergent, 2-domain PADI sequences exist in earlier diverging cyanobacteria and actinobacteria and (iv) the 2-domain PADI sequence also arose in fungi by convergent evolution.

**b) Two PADIs in the LUCA, many losses.** The number of independent losses of the 2-domain and 3-domain PADI are depicted for a scenario where the two existed as paralogous sequences in the LUCA.

**c) One PADI transferred by endosymbiont gene transfer (EGT), many losses and convergence.** An alternative scenario, where one PADI sequence existed in the LUCA and was transferred to the LECA by (EGT) also requires extensive gene losses and independent and convergent evolution of the second PADI sequence in actinobacteria and fungi.

**d) Two PADIs transferred by EGT, many losses.** The number of independent losses of the 2-domain and 3-domain PADI are depicted for a scenario where two paralogous PADI sequences were transferred to the LECA by EGT.

# Figure S9

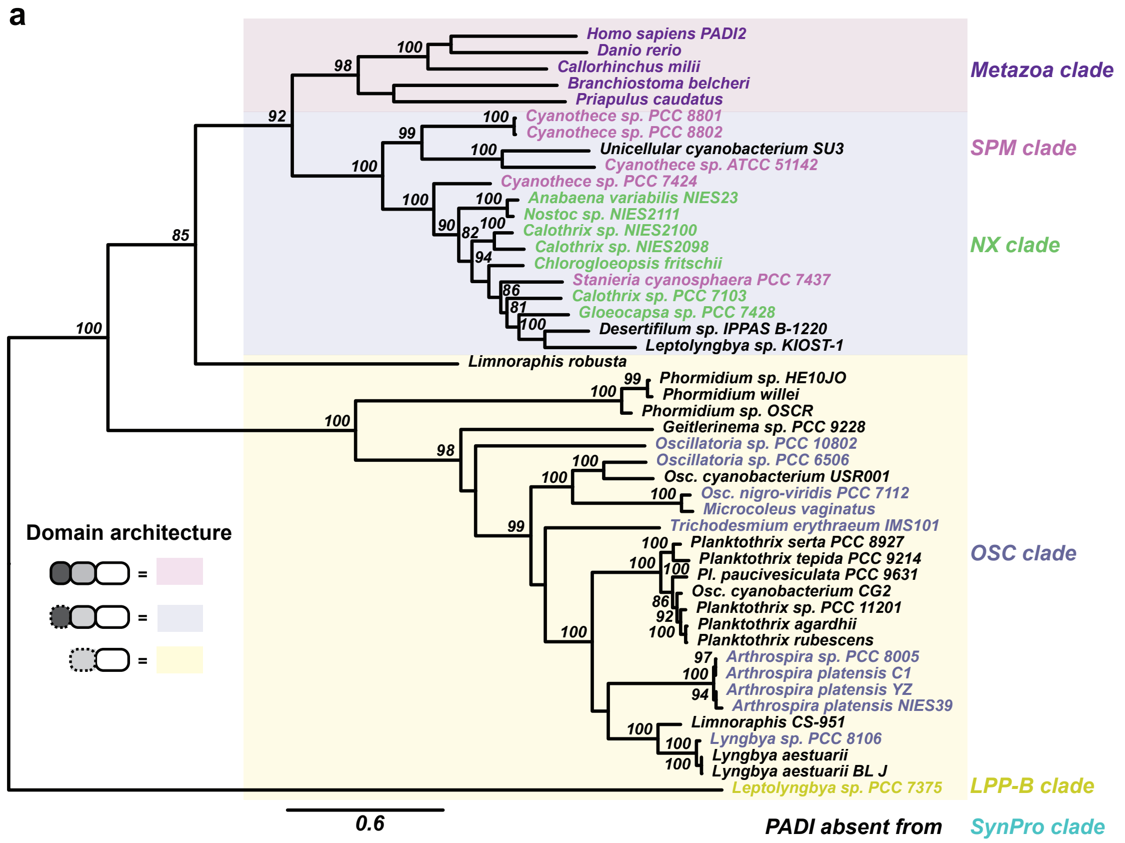


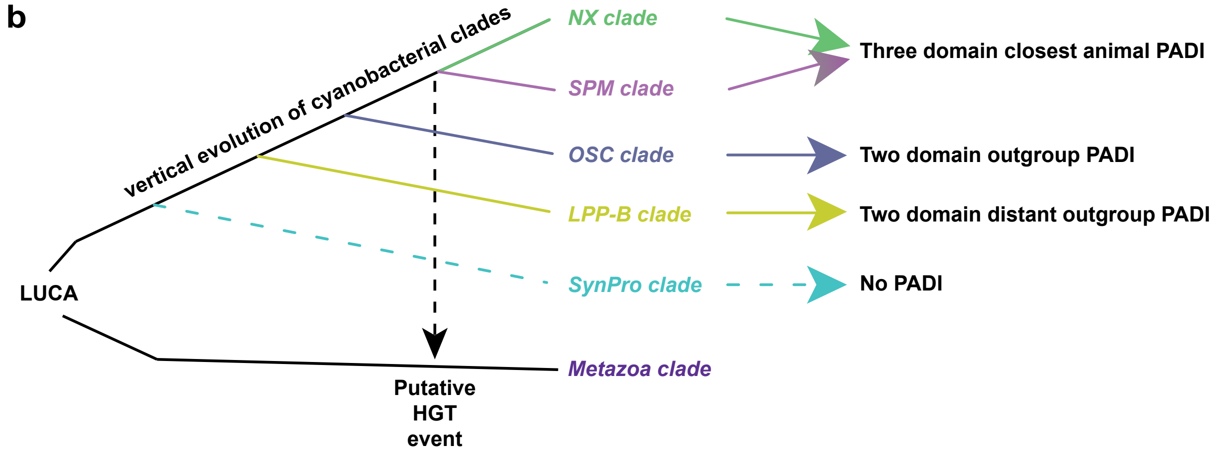


**Figure S9: Cyanobacterial origin of the PADI sequence. a)** Phylogenetic analysis of cyanobacterial PADI sequences reproduces the known clades of cyanobacterial evolution. Colours and names of clades are used as in Uyeda *et al*. Species that were not analysed in Uyeda *et al*. are in black. Sequences were aligned with MUSCLE and the tree was built using PhyML 3.0. Bootstrap support >80% is indicated on branches from 250 bootstrap replicates. The domain architecture of each sequence was analysed and is represented in the legend. **b)** Schematic showing the proposed HGT event from cyanobacteria to metazoa. PADI present in a last common ancestor of the *NX/SPM* clades of cyanobacteria, which possess a three-domain PADI, and was transferred to an ancient last common ancestor in the metazoan lineage.

## Figure S10

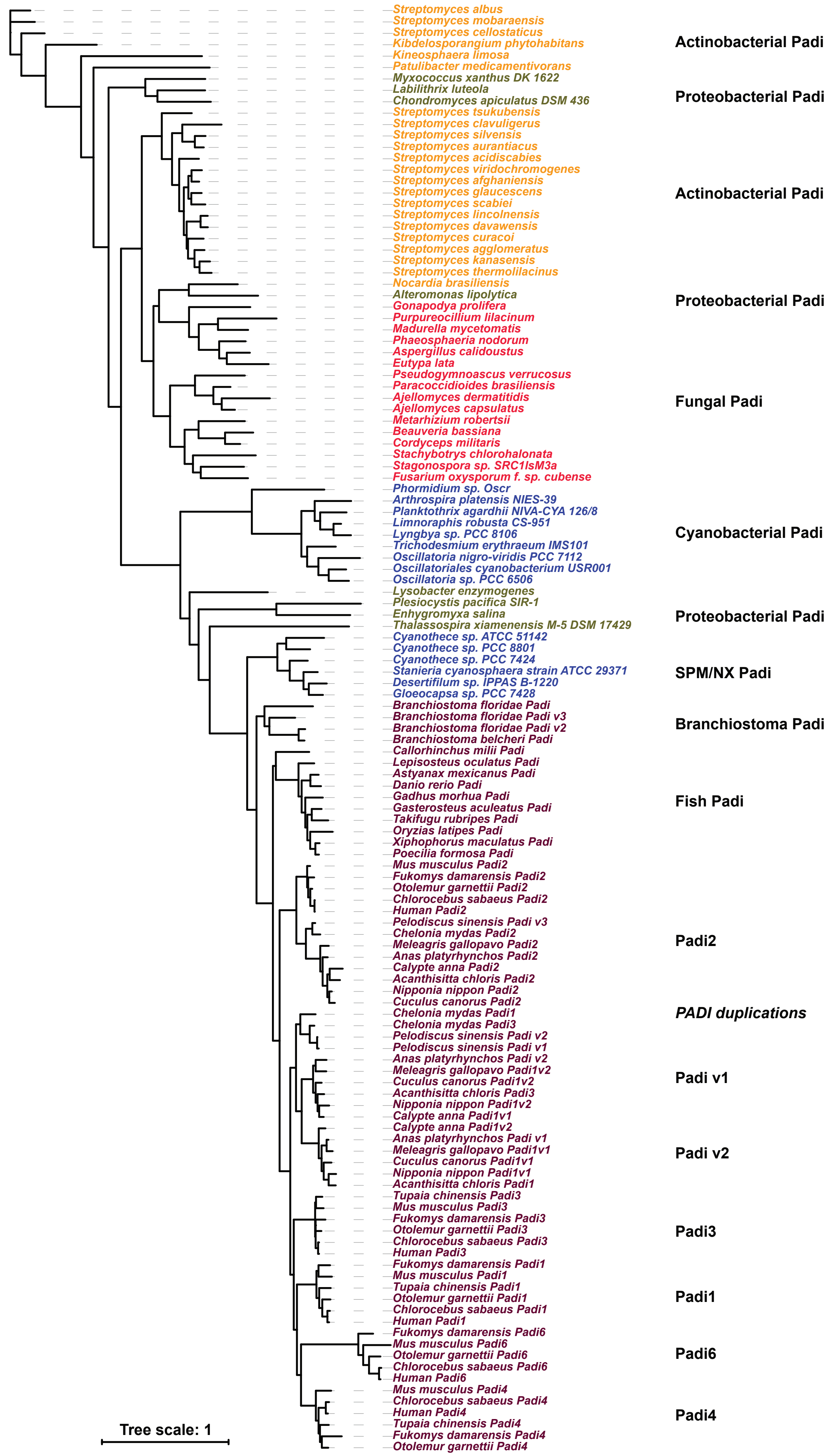
Streptomyces albus
Streptomyces mobaraensis
Streptomyces cellostaticus
Kibdelosporangium phytohabitans
Kineosphaera limosa
Patulibacter medicamentivorans
Myxococcus xanthus DK 1622
Labilithrix luteola
Chondromyces apiculatus DSM 436
Streptomyces tsukubensis
Streptomyces clavuligerus
Streptomyces silvensis
Streptomyces aurantiacus
Streptomyces acidiscabies
Streptomyces viridochromogenes
Streptomyces afghaniensis
Streptomyces glaucescens
Streptomyces scabiei
Streptomyces lincolnensis
Streptomyces davawensis
Streptomyces curacoi
Streptomyces agglomeratus
Streptomyces kanasensis
Streptomyces thermolilacinus
Nocardia brasiliensis
Alteromonas lipolytica
Gonapodya prolifera
Purpureocillium lilacinum
Madurella mycetomatis
Phaeosphaeria nodorum
Aspergillus calidoustus
Eutypa lata
Pseudogymnoascus verrucosus
Paracoccidioides brasiliensis
Ajellomyces dermatitidis
Ajellomyces capsulatus
Metarhizium robertsii
Beauveria bassiana
Cordyceps militaris
Stachybotrys chlorohalonata
Stagonospora sp. SRC1lsM3a
Fusarium oxysporum f. sp. cubense
Phormidium sp. Oscr
Arthrospira platensis NIES-39
Planktothrix agardhii NIVA-CYA 126/8
Limnoraphis robusta CS-951
Lyngbya sp. PCC 8106
Trichodesmium erythraeum IMS101
Oscillatoria nigro-viridis PCC 7112
Oscillatoriales cyanobacterium USR001
Oscillatoria sp. PCC 6506
Lysobacter enzymogenes
Plesiocystis pacifica SIR-1
Enhygromyxa salina
Thalassospira xiamenensis M-5 DSM 17429
Cyanothece sp. ATCC 51142
Cyanothece sp. PCC 8801
Cyanothece sp. PCC 7424
Stanieria cyanosphaera strain ATCC 29371
Desertifilum sp. IPPAS B-1220
Gloeocapsa sp. PCC 7428
Branchiostoma floridae Padi
Branchiostoma floridae Padi v3
Branchiostoma floridae Padi v2
Branchiostoma belcheri Padi
Callorhinchus milii Padi
Lepisosteus oculatus Padi
Astyanax mexicanus Padi
Danio rerio Padi
Gadhus morhua Padi
Gasterosteus aculeatus Padi
Takifugu rubripes Padi
Oryzias latipes Padi
Xiphophorus maculatus Padi
Poecilia formosa Padi
Mus musculus Padi2
Fukomys damarensis Padi2
Otolemur garnettii Padi2
Chlorocebus sabaeus Padi2
Human Padi2
Pelodiscus sinensis Padi v3
Chelonia mydas Padi2
Meleagris gallopavo Padi2
Anas platyrhynchos Padi2
Calypte anna Padi2
Acanthisitta chloris Padi2
Nipponia nippon Padi2
Cuculus canorus Padi2
Chelonia mydas Padi1
Chelonia mydas Padi3
Pelodiscus sinensis Padi v2
Pelodiscus sinensis Padi v1
Anas platyrhynchos Padi v2
Meleagris gallopavo Padi1v2
Cuculus canorus Padi1v2
Acanthisitta chloris Padi3
Nipponia nippon Padi1v2
Calypte anna Padi1v1
Calypte anna Padi1v2
Anas platyrhynchos Padi v1
Meleagris gallopavo Padi1v1
Cuculus canorus Padi1v1
Nipponia nippon Padi1v1
Acanthisitta chloris Padi1
Tupaia chinensis Padi3
Mus musculus Padi3
Fukomys damarensis Padi3
Otolemur garnettii Padi3
Chlorocebus sabaeus Padi3
Human Padi3
Fukomys damarensis Padi1
Mus musculus Padi1
Tupaia chinensis Padi1
Otolemur garnettii Padi1
Chlorocebus sabaeus Padi1
Human Padi1
Fukomys damarensis Padi6
Mus musculus Padi6
Otolemur garnettii Padi6
Chlorocebus sabaeus Padi6
Human Padi6
Mus musculus Padi4
Chlorocebus sabaeus Padi4
Human Padi4
Tupaia chinensis Padi4
Fukomys damarensis Padi4
Otolemur garnettii Padi4
Actinobacterial Padi
Proteobacterial Padi
Actinobacterial Padi
Proteobacterial Padi
Fungal Padi
Cyanobacterial Padi
Proteobacterial Padi
SPM/NX Padi
Branchiostoma Padi
Fish Padi
Padi2
PADI duplications
Padi v1
Padi v2
Padi3
Padi1
Padi6
Padi4
Tree scale: 1

**Figure S10: The PADI sequence was retained and underwent multiple duplications in metazoa.** Phylogenetic analysis of a large number of putative PADI sequences showing the multiple duplications in metazoa. The first duplication produced two new orthologues present in reptiles and birds which cluster in their own groups denoted PADIv1 and PADIv2. Subsequent duplications in mammals produced the separation of clusters of PADI1, PADI3, PADI4 and PADI6.
