# Supplementary material for: Citrullination Was Introduced into Animals by Horizontal Gene Transfer from Cyanobacteria
Source: Mol Biol Evol. 2021 Nov 3;39(2):msab317. doi: 10.1093/molbev/msab317 (PMC8826395; doi:10.1093/molbev/msab317)
Supplement: msab317_Supplementary_Data [file msab317_supplementary_data.zip › Suppl_File_5_Full_Trees.pdf]

**Supplementary File 3: Trees for different phylogenetic analyses using parameter rich models.** Ultrafast bootstrap 2 values with 1000 replicates for trees 1, 3, 4, 5, 6, 7; Felsenstein bootstrap values with 100 replicates for tree 2; or posterior probabilities for trees 10 and 11 are presented on each tree. **Tree 1)** WAG+R5+FO; **2)** WAG+R5+FO with Felsenstein bootstrapping; **3)** LG+R6+FO; **4)** C20+FO; **5)** WAG+F+C20+R5; **6)** WAG+FO+H4; **7)** WAG+FO*H4; **8)** Constraint tree using the model from Tree 1; **9)** Constraint tree using the model from Tree 5; **10)** Bayesian phylogenetic analysis using MrBayes; **11)** Bayesian phylogenetic analysis using PhyloBayes under the CAT-GTR model. Constrained nodes (for which the bootstrap value was constrained to be 100) are denoted in red.

1

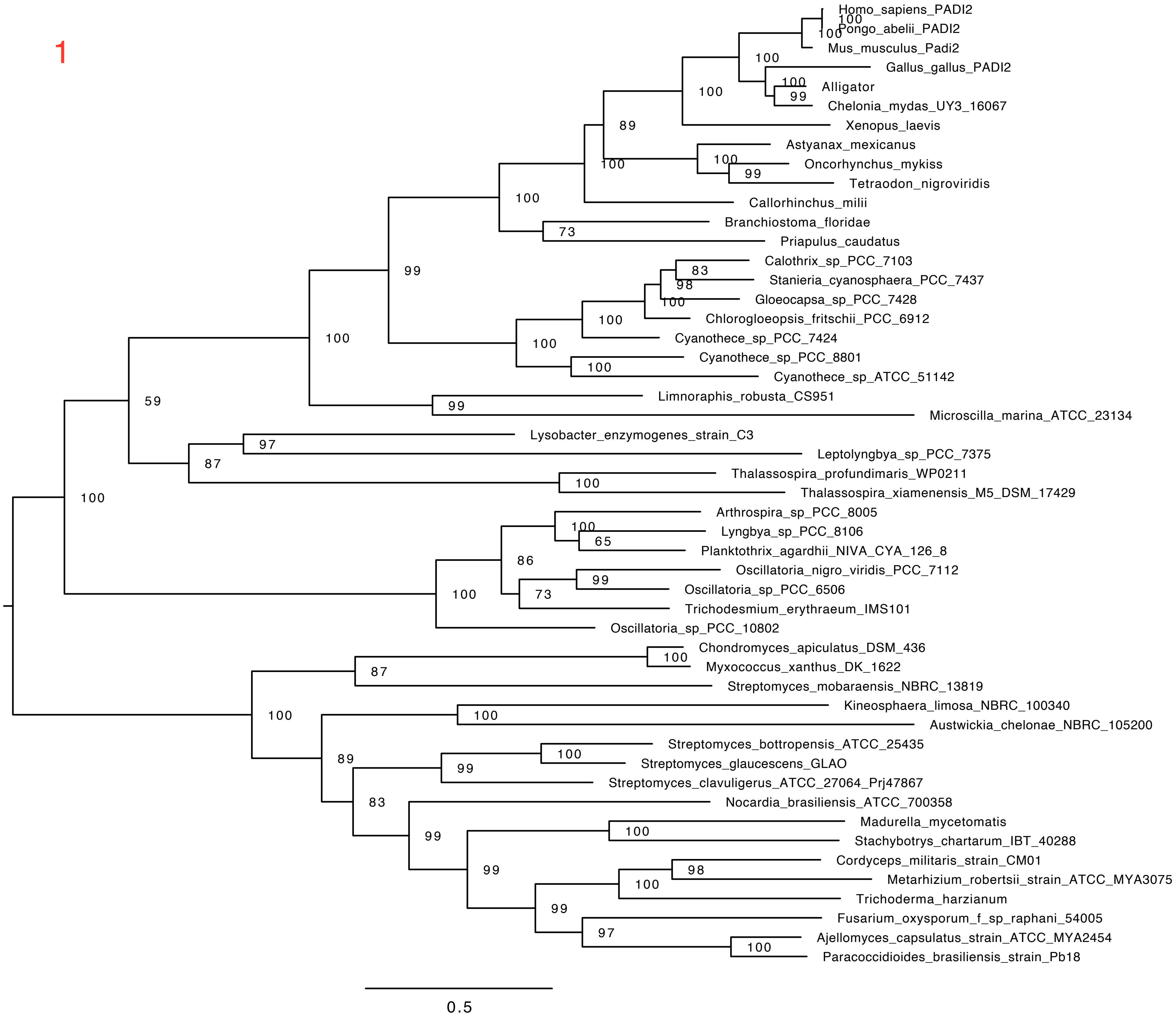
Homo_sapiens_PADI2
Pongo_abelii_PADI2
Mus_musculus_Padi2
Gallus_gallus_PADI2
Alligator
Chelonia_mydas_UY3_16067
Xenopus_laevis
Astyanax_mexicanus
Oncorhynchus_mykiss
Tetraodon_nigroviridis
Callorhinchus_milii
Branchiostoma_floridae
Priapulus_caudatus
Calothrix_sp_PCC_7103
Stanieria_cyanosphaera_PCC_7437
Gloeocapsa_sp_PCC_7428
Chlorogloeopsis_fritschii_PCC_6912
Cyanothece_sp_PCC_7424
Cyanothece_sp_PCC_8801
Cyanothece_sp_ATCC_51142
Limnoraphis_robusta_CS951
Microscilla_marina_ATCC_23134
Lysobacter_enzymogenes_strain_C3
Leptolyngbya_sp_PCC_7375
Thalassospira_profundimaris_WP0211
Thalassospira_xiamenensis_M5_DSM_17429
Arthrospira_sp_PCC_8005
Lyngbya_sp_PCC_8106
Planktothrix_agardhii_NIVA_CYA_126_8
Oscillatoria_nigro_viridis_PCC_7112
Oscillatoria_sp_PCC_6506
Trichodesmium_erythraeum_IMS101
Oscillatoria_sp_PCC_10802
Chondromyces_apiculatus_DSM_436
Myxococcus_xanthus_DK_1622
Streptomyces_mobaraensis_NBRC_13819
Kineosphaera_limosa_NBRC_100340
Austwickia_chelonae_NBRC_105200
Streptomyces_bottropensis_ATCC_25435
Streptomyces_glaucescens_GLAO
Streptomyces_clavuligerus_ATCC_27064_Prj47867
Nocardia_brasiliensis_ATCC_700358
Madurella_mycetomatis
Stachybotrys_chartarum_IBT_40288
Cordyceps_militaris_strain_CM01
Metarhizium_robertsii_strain_ATCC_MYA3075
Trichoderma_harzianum
Fusarium_oxysporum_f_sp_raphani_54005
Ajellomyces_capsulatus_strain_ATCC_MYA2454
Paracoccidioides_brasiliensis_strain_Pb18
0.5

2

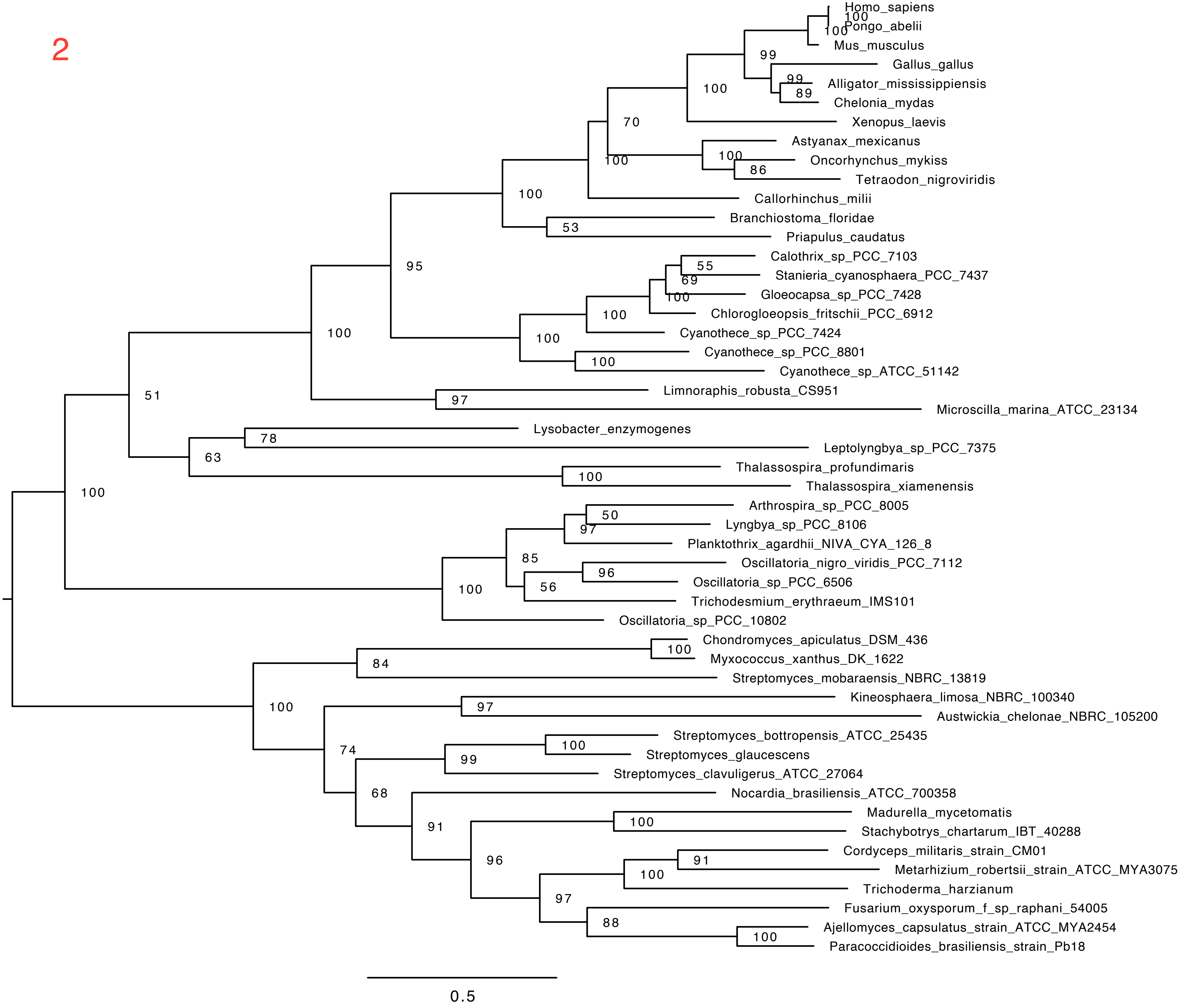
Homo_sapiens
Pongo_abelii
Mus_musculus
Gallus_gallus
Alligator_mississippiensis
Chelonia_mydas
Xenopus_laevis
Astyanax_mexicanus
Oncorhynchus_mykiss
Tetraodon_nigroviridis
Callorhinchus_milii
Branchiostoma_floridae
Priapulus_caudatus
Calothrix_sp_PCC_7103
Stanieria_cyanosphaera_PCC_7437
Gloeocapsa_sp_PCC_7428
Chlorogloeopsis_fritschii_PCC_6912
Cyanothece_sp_PCC_7424
Cyanothece_sp_PCC_8801
Cyanothece_sp_ATCC_51142
Limnoraphis_robusta_CS951
Microscilla_marina_ATCC_23134
Lysobacter_enzymogenes
Leptolyngbya_sp_PCC_7375
Thalassospira_profundimaris
Thalassospira_xiamenensis
Arthrospira_sp_PCC_8005
Lyngbya_sp_PCC_8106
Planktothrix_agardhii_NIVA_CYA_126_8
Oscillatoria_nigro_viridis_PCC_7112
Oscillatoria_sp_PCC_6506
Trichodesmium_erythraeum_IMS101
Oscillatoria_sp_PCC_10802
Chondromyces_apiculatus_DSM_436
Myxococcus_xanthus_DK_1622
Streptomyces_mobaraensis_NBRC_13819
Kineosphaera_limosa_NBRC_100340
Austwickia_chelonae_NBRC_105200
Streptomyces_bottropensis_ATCC_25435
Streptomyces_glaucescens
Streptomyces_clavuligerus_ATCC_27064
Nocardia_brasiliensis_ATCC_700358
Madurella_mycetomatis
Stachybotrys_chartarum_IBT_40288
Cordyceps_militaris_strain_CM01
Metarhizium_robertsii_strain_ATCC_MYA3075
Trichoderma_harzianum
Fusarium_oxysporum_f_sp_raphani_54005
Ajellomyces_capsulatus_strain_ATCC_MYA2454
Paracoccidioides_brasiliensis_strain_Pb18
0.5

3

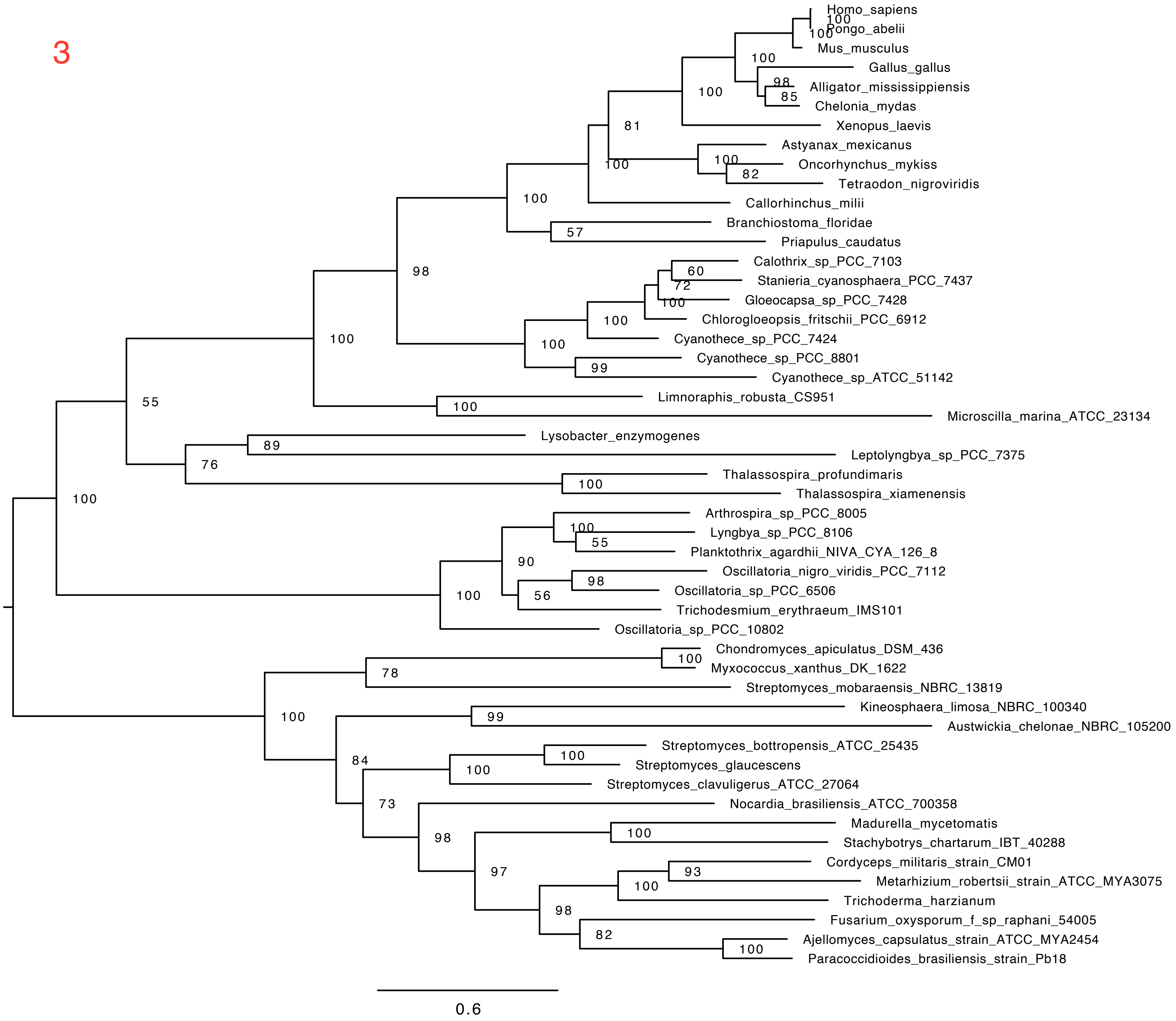
Homo_sapiens
Pongo_abelii
Mus_musculus
Gallus_gallus
Alligator_mississippiensis
Chelonia_mydas
Xenopus_laevis
Astyanax_mexicanus
Oncorhynchus_mykiss
Tetraodon_nigroviridis
Callorhinchus_milii
Branchiostoma_floridae
Priapulus_caudatus
Calothrix_sp_PCC_7103
Stanieria_cyanosphaera_PCC_7437
Gloeocapsa_sp_PCC_7428
Chlorogloeopsis_fritschii_PCC_6912
Cyanothece_sp_PCC_7424
Cyanothece_sp_PCC_8801
Cyanothece_sp_ATCC_51142
Limnoraphis_robusta_CS951
Microscilla_marina_ATCC_23134
Lysobacter_enzymogenes
Leptolyngbya_sp_PCC_7375
Thalassospira_profundimaris
Thalassospira_xiamenensis
Arthrospira_sp_PCC_8005
Lyngbya_sp_PCC_8106
Planktothrix_agardhii_NIVA_CYA_126_8
Oscillatoria_nigro_viridis_PCC_7112
Oscillatoria_sp_PCC_6506
Trichodesmium_erythraeum_IMS101
Oscillatoria_sp_PCC_10802
Chondromyces_apiculatus_DSM_436
Myxococcus_xanthus_DK_1622
Streptomyces_mobaraensis_NBRC_13819
Kineosphaera_limosa_NBRC_100340
Austwickia_chelonae_NBRC_105200
Streptomyces_bottropensis_ATCC_25435
Streptomyces_glaucescens
Streptomyces_clavuligerus_ATCC_27064
Nocardia_brasiliensis_ATCC_700358
Madurella_mycetomatis
Stachybotrys_chartarum_IBT_40288
Cordyceps_militaris_strain_CM01
Metarhizium_robertsii_strain_ATCC_MYA3075
Trichoderma_harzianum
Fusarium_oxysporum_f_sp_raphani_54005
Ajellomyces_capsulatus_strain_ATCC_MYA2454
Paracoccidioides_brasiliensis_strain_Pb18
0.6

4

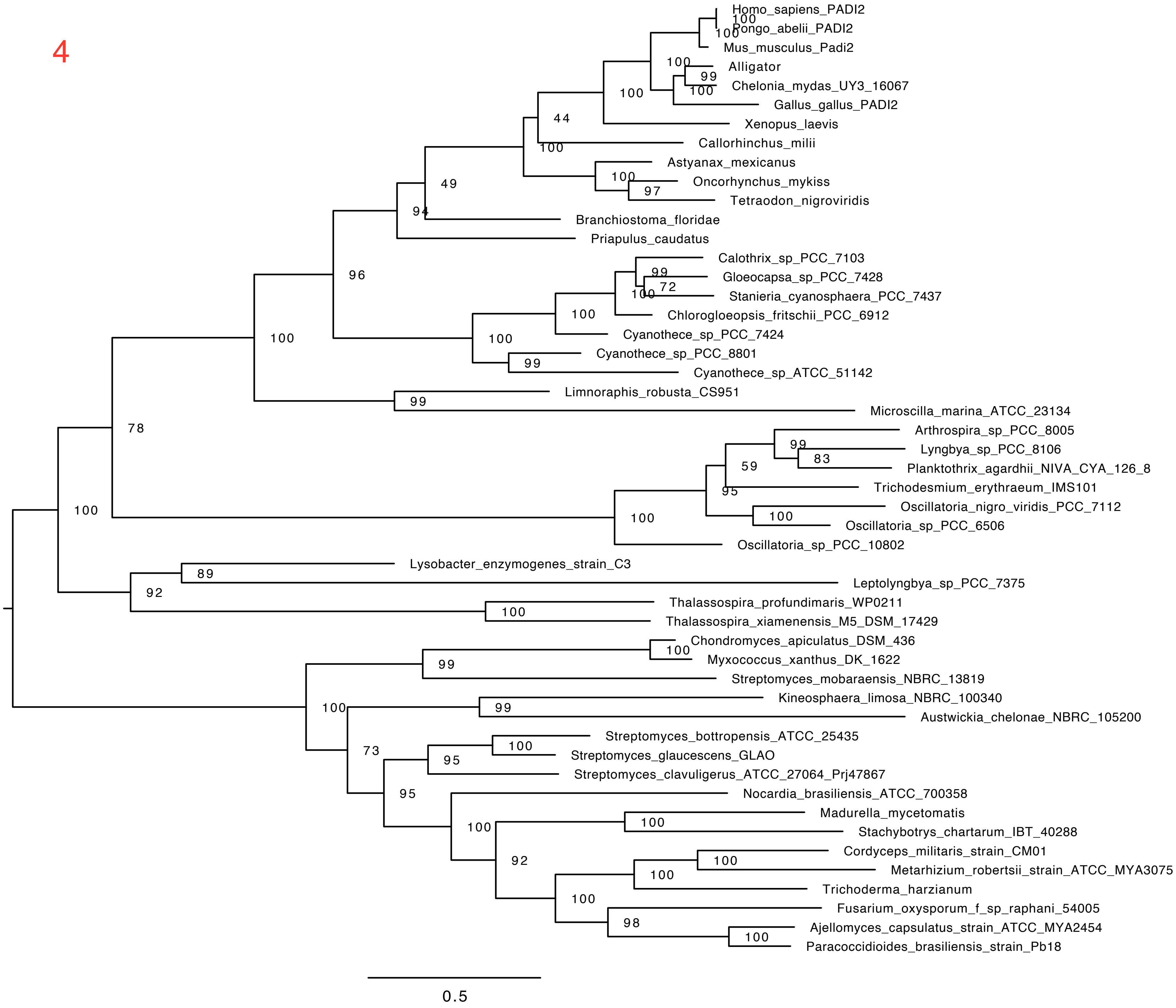

Homo_sapiens_PADI2
100
Pongo_abelii_PADI2
100
Mus_musculus_Padi2
100
Alligator
99
Chelonia_mydas_UY3_16067
100
Gallus_gallus_PADI2
100
Xenopus_laevis
44
Callorhinchus_milii
100
Astyanax_mexicanus
100
Oncorhynchus_mykiss
97
Tetraodon_nigroviridis
49
94
Branchiostoma_floridae
Priapulus_caudatus
96
Calothrix_sp_PCC_7103
99
Gloeocapsa_sp_PCC_7428
72
100
Stanieria_cyanosphaera_PCC_7437
100
Chlorogloeopsis_fritschii_PCC_6912
Cyanothece_sp_PCC_7424
100
100
Cyanothece_sp_PCC_8801
99
Cyanothece_sp_ATCC_51142
Limnoraphis_robusta_CS951
99
Microscilla_marina_ATCC_23134
78
Arthrospira_sp_PCC_8005
99
Lyngbya_sp_PCC_8106
83
59
Planktothrix_agardhii_NIVA_CYA_126_8
Trichodesmium_erythraeum_IMS101
95
Oscillatoria_nigro_viridis_PCC_7112
100
100
Oscillatoria_sp_PCC_6506
100
Oscillatoria_sp_PCC_10802
Lysobacter_enzymogenes_strain_C3
89
Leptolyngbya_sp_PCC_7375
92
Thalassospira_profundimaris_WP0211
100
Thalassospira_xiamenensis_M5_DSM_17429
Chondromyces_apiculatus_DSM_436
100
Myxococcus_xanthus_DK_1622
99
Streptomyces_mobaraensis_NBRC_13819
Kineosphaera_limosa_NBRC_100340
100
99
Austwickia_chelonae_NBRC_105200
Streptomyces_bottropensis_ATCC_25435
73
100
Streptomyces_glaucescens_GLAO
95
Streptomyces_clavuligerus_ATCC_27064_Prj47867
95
Nocardia_brasiliensis_ATCC_700358
Madurella_mycetomatis
100
100
Stachybotrys_chartarum_IBT_40288
Cordyceps_militaris_strain_CM01
92
100
Metarhizium_robertsii_strain_ATCC_MYA3075
100
Trichoderma_harzianum
100
Fusarium_oxysporum_f_sp_raphani_54005
98
Ajellomyces_capsulatus_strain_ATCC_MYA2454
100
Paracoccidioides_brasiliensis_strain_Pb18
0.5

5

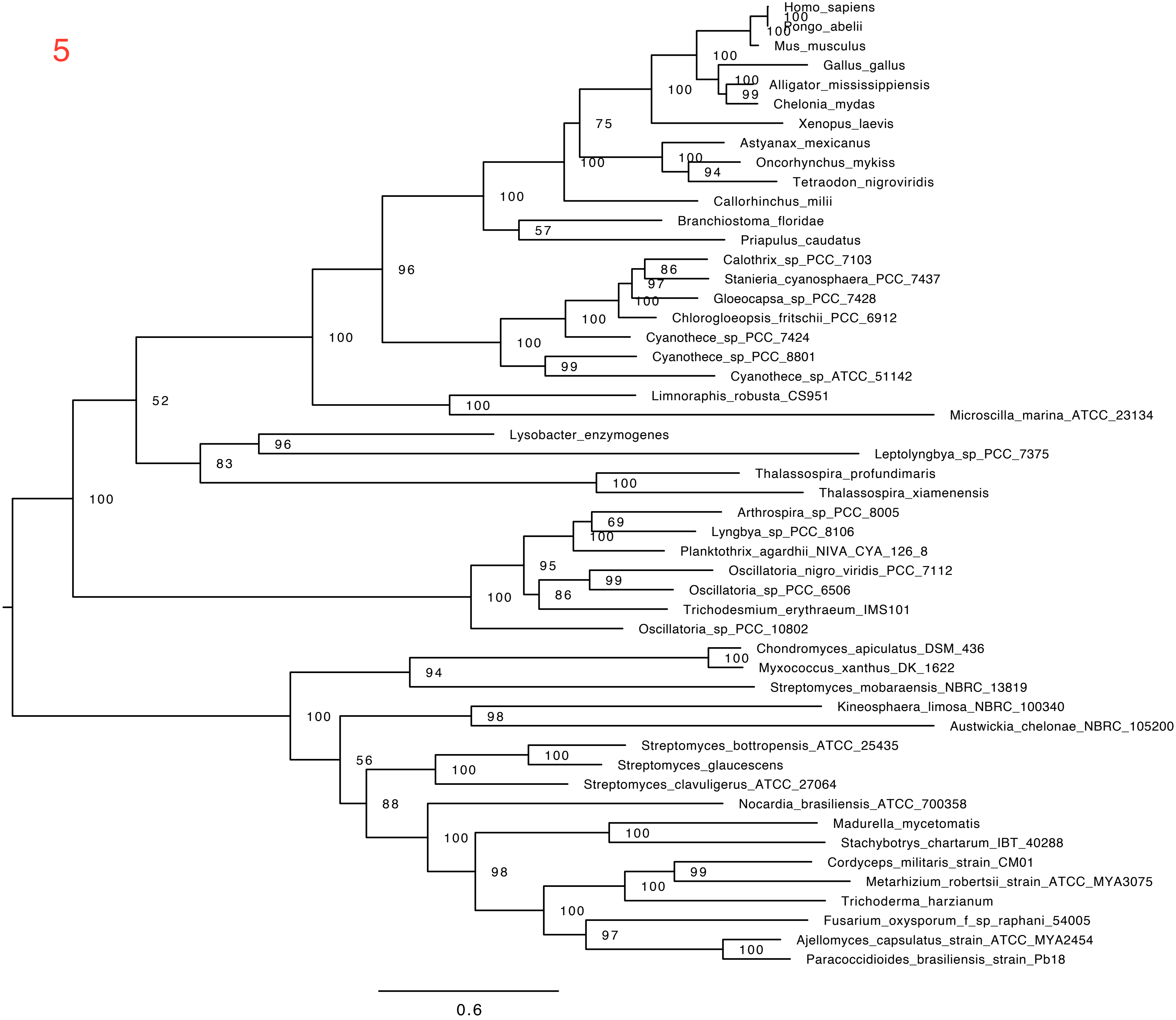
Homo_sapiens
Pongo_abelii
Mus_musculus
Gallus_gallus
Alligator_mississippiensis
Chelonia_mydas
Xenopus_laevis
Astyanax_mexicanus
Oncorhynchus_mykiss
Tetraodon_nigroviridis
Callorhinchus_milii
Branchiostoma_floridae
Priapulus_caudatus
Calothrix_sp_PCC_7103
Stanieria_cyanosphaera_PCC_7437
Gloeocapsa_sp_PCC_7428
Chlorogloeopsis_fritschii_PCC_6912
Cyanothece_sp_PCC_7424
Cyanothece_sp_PCC_8801
Cyanothece_sp_ATCC_51142
Limnoraphis_robusta_CS951
Microscilla_marina_ATCC_23134
Lysobacter_enzymogenes
Leptolyngbya_sp_PCC_7375
Thalassospira_profundimaris
Thalassospira_xiamenensis
Arthrospira_sp_PCC_8005
Lyngbya_sp_PCC_8106
Planktothrix_agardhii_NIVA_CYA_126_8
Oscillatoria_nigro_viridis_PCC_7112
Oscillatoria_sp_PCC_6506
Trichodesmium_erythraeum_IMS101
Oscillatoria_sp_PCC_10802
Chondromyces_apiculatus_DSM_436
Myxococcus_xanthus_DK_1622
Streptomyces_mobaraensis_NBRC_13819
Kineosphaera_limosa_NBRC_100340
Austwickia_chelonae_NBRC_105200
Streptomyces_bottropensis_ATCC_25435
Streptomyces_glaucescens
Streptomyces_clavuligerus_ATCC_27064
Nocardia_brasiliensis_ATCC_700358
Madurella_mycetomatis
Stachybotrys_chartarum_IBT_40288
Cordyceps_militaris_strain_CM01
Metarhizium_robertsii_strain_ATCC_MYA3075
Trichoderma_harzianum
Fusarium_oxysporum_f_sp_raphani_54005
Ajellomyces_capsulatus_strain_ATCC_MYA2454
Paracoccidioides_brasiliensis_strain_Pb18
0.6

6

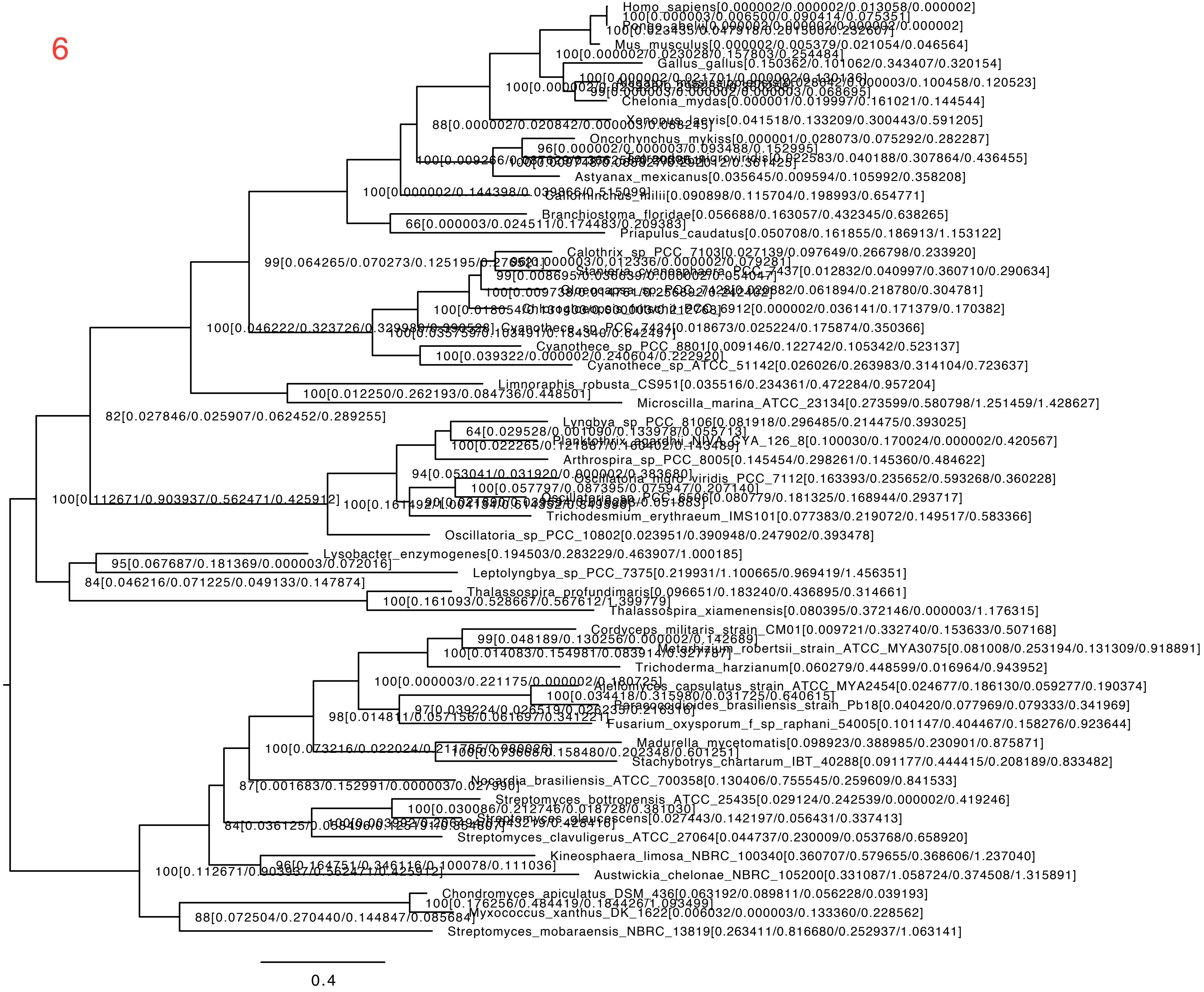

Homo_sapiens[0.000002/0.000002/0.013058/0.000002]
100[0.000003/0.006500/0.090414/0.075351]
Mus_musculus[0.000002/0.005379/0.021054/0.046564]
100[0.000002/0.023028/0.157803/0.254484]
Gallus_gallus[0.150362/0.101062/0.343407/0.320154]
Chelonia_mydas[0.000001/0.019997/0.161021/0.144544]
Xenopus_laevis[0.041518/0.133209/0.300443/0.591205]
Oncorhynchus_mykiss[0.000001/0.028073/0.075292/0.282287]
96[0.000002/0.000003/0.093488/0.152995]
Astyanax_mexicanus[0.035645/0.009594/0.105992/0.358208]
Callorhinchus_milii[0.090898/0.115704/0.198993/0.654771]
Branchiostoma_floridae[0.056688/0.163057/0.432345/0.638265]
66[0.000003/0.024511/0.174483/0.209383]
Priapulus_caudatus[0.050708/0.161855/0.186913/1.153122]
Calothrix_sp_PCC_7103[0.027139/0.097649/0.266798/0.233920]
Cyanothece_sp_PCC_8801[0.009146/0.122742/0.105342/0.523137]
100[0.039322/0.000002/0.240604/0.222920]
Cyanothece_sp_ATCC_51142[0.026026/0.263983/0.314104/0.723637]
Limnoraphis_robusta_CS951[0.035516/0.234361/0.472284/0.957204]
100[0.012250/0.262193/0.084736/0.448501]
Microscilla_marina_ATCC_23134[0.273599/0.580798/1.251459/1.428627]
82[0.027846/0.025907/0.062452/0.289255]
Lyngbya_sp_PCC_8106[0.081918/0.296485/0.214475/0.393025]
Arthrospira_sp_PCC_8005[0.145454/0.298261/0.145360/0.484622]
100[0.057797/0.087395/0.075947/0.207140]
100[0.112671/0.903937/0.562471/0.425912]
Trichodesmium_erythraeum_IMS101[0.077383/0.219072/0.149517/0.583366]
Oscillatoria_sp_PCC_10802[0.023951/0.390948/0.247902/0.393478]
Lysobacter_enzymogenes[0.194503/0.283229/0.463907/1.000185]
95[0.067687/0.181369/0.000003/0.072016]
Leptolyngbya_sp_PCC_7375[0.219931/1.100665/0.969419/1.456351]
84[0.046216/0.071225/0.049133/0.147874]
Thalassospira_profundimaris[0.096651/0.183240/0.436895/0.314661]
100[0.161093/0.528667/0.567612/1.399779]
Thalassospira_xiamenensis[0.080395/0.372146/0.000003/1.176315]
Cordyceps_militaris_strain_CM01[0.009721/0.332740/0.153633/0.507168]
99[0.048189/0.130256/0.000002/0.142689]
Trichoderma_harzianum[0.060279/0.448599/0.016964/0.943952]
100[0.000003/0.221175/0.000002/0.180725]
100[0.034418/0.315980/0.031725/0.640615]
Fusarium_oxysporum_f_sp_raphani_54005[0.101147/0.404467/0.158276/0.923644]
Madurella_mycetomatis[0.098923/0.388985/0.230901/0.875871]
Stachybotrys_chartarum_IBT_40288[0.091177/0.444415/0.208189/0.833482]
Nocardia_brasiliensis_ATCC_700358[0.130406/0.755545/0.259609/0.841533]
87[0.001683/0.152991/0.000003/0.027990]
Streptomyces_bottropensis_ATCC_25435[0.029124/0.242539/0.000002/0.419246]
100[0.030086/0.212746/0.018728/0.381030]
Streptomyces_clavuligerus_ATCC_27064[0.044737/0.230009/0.053768/0.658920]
Kineosphaera_limosa_NBRC_100340[0.360707/0.579655/0.368606/1.237040]
96[0.164751/0.346116/0.100078/0.111036]
Austwickia_chelonae_NBRC_105200[0.331087/1.058724/0.374508/1.315891]
Chondromyces_apiculatus_DSM_436[0.063192/0.089811/0.056228/0.039193]
100[0.176256/0.484419/0.184426/1.093499]
Myxococcus_xanthus_DK_1622[0.006032/0.000003/0.133360/0.228562]
88[0.072504/0.270440/0.144847/0.085684]
Streptomyces_mobaraensis_NBRC_13819[0.263411/0.816680/0.252937/1.063141]
0.4

7

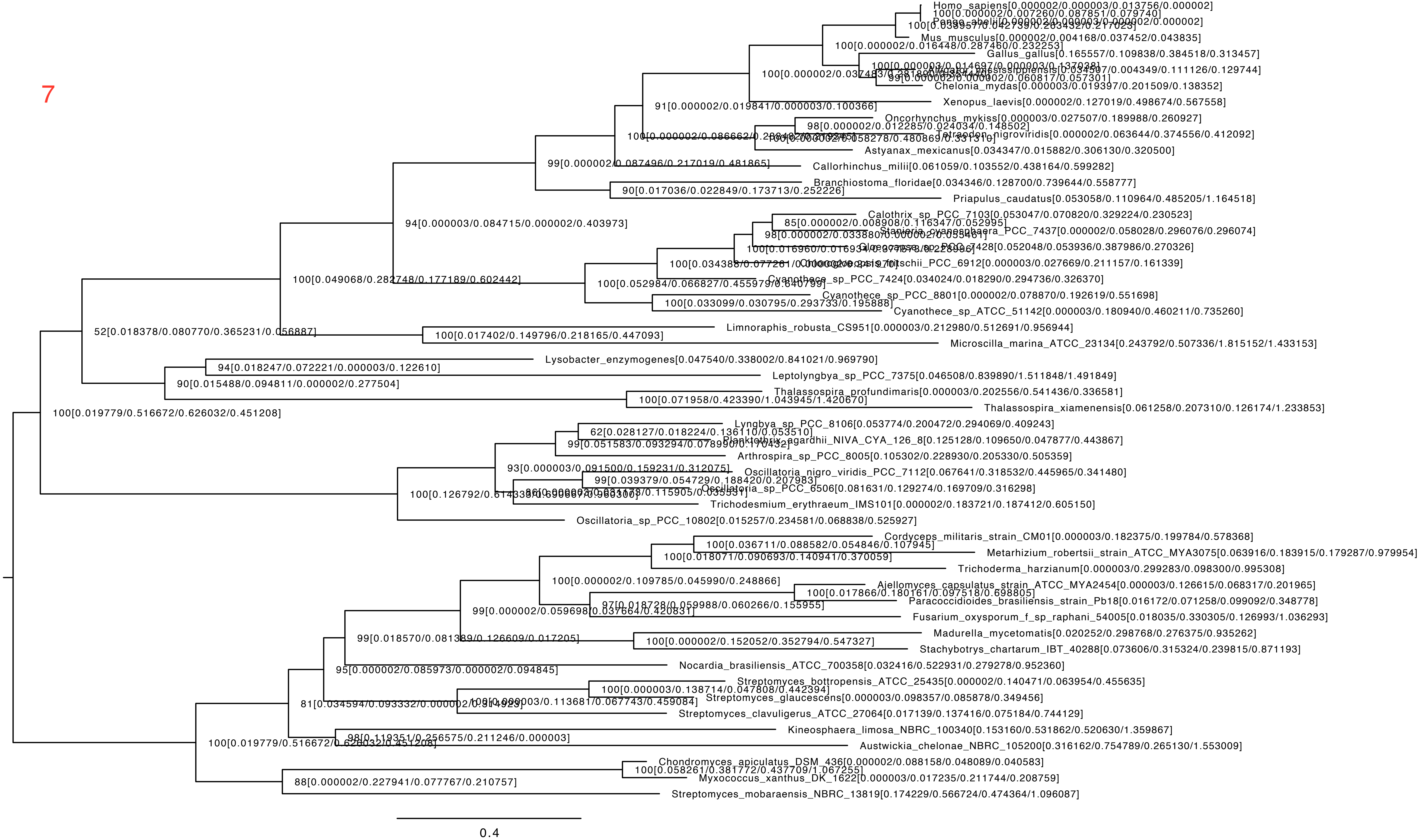

Homo_sapiens[0.000002/0.000003/0.013756/0.000002]
100[0.000002/0.007260/0.087851/0.079740]
Mus_musculus[0.000002/0.004168/0.037452/0.043835]
100[0.000002/0.016448/0.287460/0.232253]
Gallus_gallus[0.165557/0.109838/0.384518/0.313457]
Chelonia_mydas[0.000003/0.019397/0.201509/0.138352]
Xenopus_laevis[0.000002/0.127019/0.498674/0.567558]
91[0.000002/0.019841/0.000003/0.100366]
Oncorhynchus_mykiss[0.000003/0.027507/0.189988/0.260927]
98[0.000002/0.012285/0.024034/0.148502]
Tetraodon_nigroviridis[0.000002/0.063644/0.374556/0.412092]
Astyanax_mexicanus[0.034347/0.015882/0.306130/0.320500]
99[0.000002/0.087496/0.217019/0.481865]
Callorhinchus_milii[0.061059/0.103552/0.438164/0.599282]
Branchiostoma_floridae[0.034346/0.128700/0.739644/0.558777]
90[0.017036/0.022849/0.173713/0.252226]
Priapulus_caudatus[0.053058/0.110964/0.485205/1.164518]
Calothrix_sp_PCC_7103[0.053047/0.070820/0.329224/0.230523]
85[0.000002/0.008908/0.116347/0.052995]
94[0.000003/0.084715/0.000002/0.403973]
Gloeocapsa_sp_PCC_7428[0.052048/0.053936/0.387986/0.270326]
Chroococcidiopsis_thermalis_PCC_7203
Cyanothece_sp_PCC_7424[0.034024/0.018290/0.294736/0.326370]
100[0.049068/0.282748/0.177189/0.602442]
100[0.052984/0.066827/0.455979/0.640799]
Cyanothece_sp_PCC_8801[0.000002/0.078870/0.192619/0.551698]
100[0.033099/0.030795/0.293733/0.195888]
Cyanothece_sp_ATCC_51142[0.000003/0.180940/0.460211/0.735260]
Limnoraphis_robusta_CS951[0.000003/0.212980/0.512691/0.956944]
52[0.018378/0.080770/0.365231/0.056887]
100[0.017402/0.149796/0.218165/0.447093]
Microscilla_marina_ATCC_23134[0.243792/0.507336/1.815152/1.433153]
Lysobacter_enzymogenes[0.047540/0.338002/0.841021/0.969790]
94[0.018247/0.072221/0.000003/0.122610]
Leptolyngbya_sp_PCC_7375[0.046508/0.839890/1.511848/1.491849]
90[0.015488/0.094811/0.000002/0.277504]
Thalassospira_profundimaris[0.000003/0.202556/0.541436/0.336581]
100[0.071958/0.423390/1.043945/1.420670]
Thalassospira_xiamenensis[0.061258/0.207310/0.126174/1.233853]
100[0.019779/0.516672/0.626032/0.451208]
Lyngbya_sp_PCC_8106[0.053774/0.200472/0.294069/0.409243]
62[0.028127/0.018224/0.136110/0.053510]
Planktothrix_agardhii_NIVA_CYA_126_8[0.125128/0.109650/0.047877/0.443867]
Arthrospira_sp_PCC_8005[0.105302/0.228930/0.205330/0.505359]
93[0.000003/0.091500/0.159231/0.312075]
Oscillatoria_nigro_viridis_PCC_7112[0.067641/0.318532/0.445965/0.341480]
99[0.039379/0.054729/0.188420/0.207983]
Oscillatoria_sp_PCC_6506[0.081631/0.129274/0.169709/0.316298]
Trichodesmium_erythraeum_IMS101[0.000002/0.183721/0.187412/0.605150]
Oscillatoria_sp_PCC_10802[0.015257/0.234581/0.068838/0.525927]
Cordyceps_militaris_strain_CM01[0.000003/0.182375/0.199784/0.578368]
100[0.036711/0.088582/0.054846/0.107945]
Metarhizium_robertsii_strain_ATCC_MYA3075[0.063916/0.183915/0.179287/0.979954]
100[0.018071/0.090693/0.140941/0.370059]
Trichoderma_harzianum[0.000003/0.299283/0.098300/0.995308]
100[0.000002/0.109785/0.045990/0.248866]
Ajellomyces_capsulatus_strain_ATCC_MYA2454[0.000003/0.126615/0.068317/0.201965]
100[0.017866/0.180161/0.097518/0.698805]
Paracoccidioides_brasiliensis_strain_Pb18[0.016172/0.071258/0.099092/0.348778]
97[0.018728/0.059988/0.060266/0.155955]
99[0.000002/0.059698/0.037664/0.420831]
Fusarium_oxysporum_f_sp_raphani_54005[0.018035/0.330305/0.126993/1.036293]
Madurella_mycetomatis[0.020252/0.298768/0.276375/0.935262]
99[0.018570/0.081389/0.126609/0.017205]
100[0.000002/0.152052/0.352794/0.547327]
Stachybotrys_chartarum_IBT_40288[0.073606/0.315324/0.239815/0.871193]
Nocardia_brasiliensis_ATCC_700358[0.032416/0.522931/0.279278/0.952360]
95[0.000002/0.085973/0.000002/0.094845]
Streptomyces_bottropensis_ATCC_25435[0.000002/0.140471/0.063954/0.455635]
100[0.000003/0.138714/0.047808/0.442394]
Streptomyces_glaucescens[0.000003/0.098357/0.085878/0.349456]
Streptomyces_clavuligerus_ATCC_27064[0.017139/0.137416/0.075184/0.744129]
Kineosphaera_limosa_NBRC_100340[0.153160/0.531862/0.520630/1.359867]
98[0.119351/0.256575/0.211246/0.000003]
100[0.019779/0.516672/0.626032/0.451208]
Austwickia_chelonae_NBRC_105200[0.316162/0.754789/0.265130/1.553009]
Chondromyces_apiculatus_DSM_436[0.000002/0.088158/0.048089/0.040583]
100[0.058261/0.381772/0.437709/1.067255]
Myxococcus_xanthus_DK_1622[0.000003/0.017235/0.211744/0.208759]
88[0.000002/0.227941/0.077767/0.210757]
Streptomyces_mobaraensis_NBRC_13819[0.174229/0.566724/0.474364/1.096087]
0.4

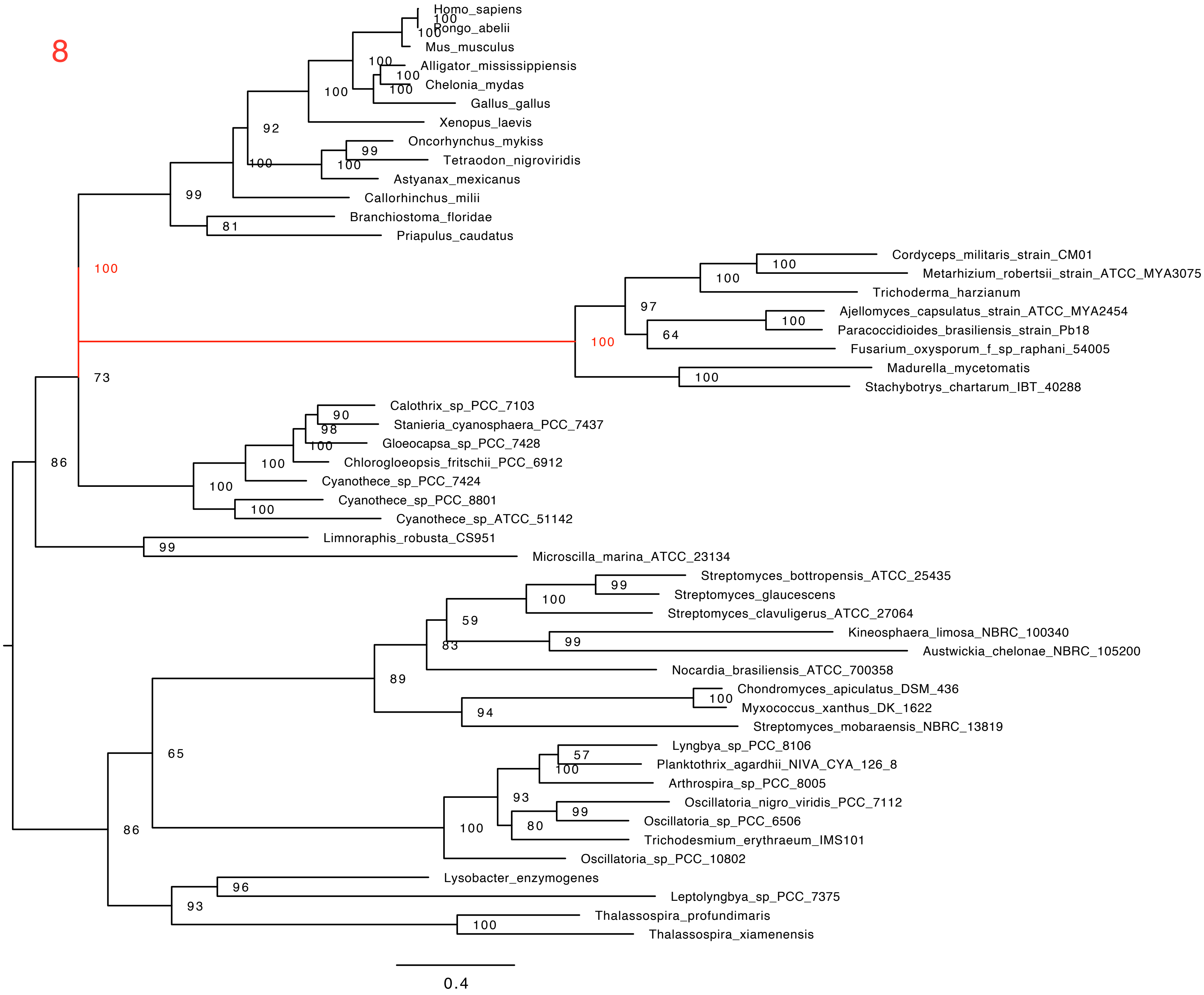
8
Homo_sapiens
Pongo_abelii
Mus_musculus
Alligator_mississippiensis
Chelonia_mydas
Gallus_gallus
Xenopus_laevis
Oncorhynchus_mykiss
Tetraodon_nigroviridis
Astyanax_mexicanus
Callorhinchus_milii
Branchiostoma_floridae
Priapulus_caudatus
Cordyceps_militaris_strain_CM01
Metarhizium_robertsii_strain_ATCC_MYA3075
Trichoderma_harzianum
Ajellomyces_capsulatus_strain_ATCC_MYA2454
Paracoccidioides_brasiliensis_strain_Pb18
Fusarium_oxysporum_f_sp_raphani_54005
Madurella_mycetomatis
Stachybotrys_chartarum_IBT_40288
Calothrix_sp_PCC_7103
Stanieria_cyanosphaera_PCC_7437
Gloeocapsa_sp_PCC_7428
Chlorogloeopsis_fritschii_PCC_6912
Cyanothece_sp_PCC_7424
Cyanothece_sp_PCC_8801
Cyanothece_sp_ATCC_51142
Limnoraphis_robusta_CS951
Microscilla_marina_ATCC_23134
Streptomyces_bottropensis_ATCC_25435
Streptomyces_glaucescens
Streptomyces_clavuligerus_ATCC_27064
Kineosphaera_limosa_NBRC_100340
Austwickia_chelonae_NBRC_105200
Nocardia_brasiliensis_ATCC_700358
Chondromyces_apiculatus_DSM_436
Myxococcus_xanthus_DK_1622
Streptomyces_mobaraensis_NBRC_13819
Lyngbya_sp_PCC_8106
Planktothrix_agardhii_NIVA_CYA_126_8
Arthrospira_sp_PCC_8005
Oscillatoria_nigro_viridis_PCC_7112
Oscillatoria_sp_PCC_6506
Trichodesmium_erythraeum_IMS101
Oscillatoria_sp_PCC_10802
Lysobacter_enzymogenes
Leptolyngbya_sp_PCC_7375
Thalassospira_profundimaris
Thalassospira_xiamenensis
0.4

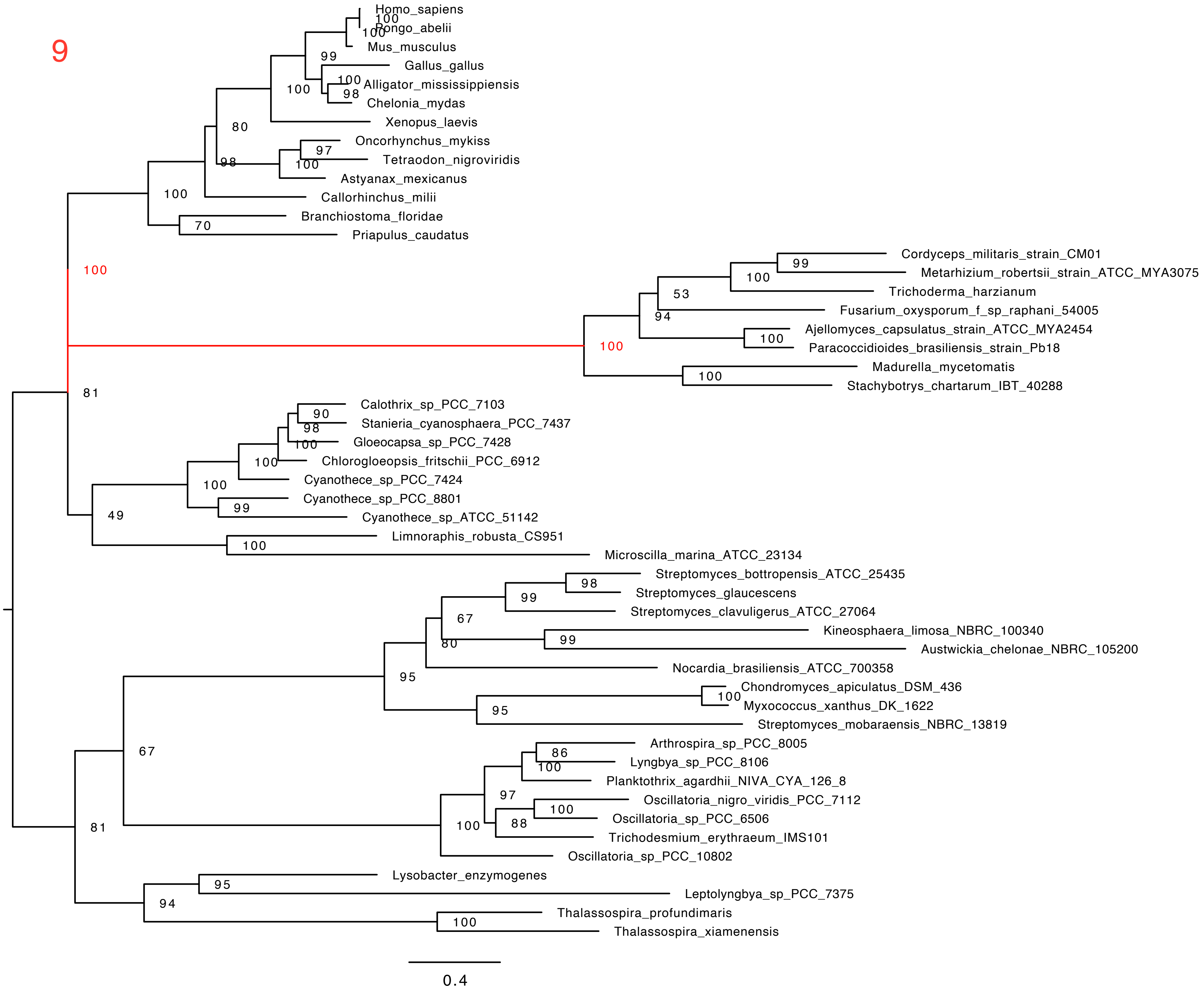
9
Homo_sapiens
Pongo_abelii
Mus_musculus
Gallus_gallus
Alligator_mississippiensis
Chelonia_mydas
Xenopus_laevis
Oncorhynchus_mykiss
Tetraodon_nigroviridis
Astyanax_mexicanus
Callorhinchus_milii
Branchiostoma_floridae
Priapulus_caudatus
Cordyceps_militaris_strain_CM01
Metarhizium_robertsii_strain_ATCC_MYA3075
Trichoderma_harzianum
Fusarium_oxysporum_f_sp_raphani_54005
Ajellomyces_capsulatus_strain_ATCC_MYA2454
Paracoccidioides_brasiliensis_strain_Pb18
Madurella_mycetomatis
Stachybotrys_chartarum_IBT_40288
Calothrix_sp_PCC_7103
Stanieria_cyanosphaera_PCC_7437
Gloeocapsa_sp_PCC_7428
Chlorogloeopsis_fritschii_PCC_6912
Cyanothece_sp_PCC_7424
Cyanothece_sp_PCC_8801
Cyanothece_sp_ATCC_51142
Limnoraphis_robusta_CS951
Microscilla_marina_ATCC_23134
Streptomyces_bottropensis_ATCC_25435
Streptomyces_glaucescens
Streptomyces_clavuligerus_ATCC_27064
Kineosphaera_limosa_NBRC_100340
Austwickia_chelonae_NBRC_105200
Nocardia_brasiliensis_ATCC_700358
Chondromyces_apiculatus_DSM_436
Myxococcus_xanthus_DK_1622
Streptomyces_mobaraensis_NBRC_13819
Arthrospira_sp_PCC_8005
Lyngbya_sp_PCC_8106
Planktothrix_agardhii_NIVA_CYA_126_8
Oscillatoria_nigro_viridis_PCC_7112
Oscillatoria_sp_PCC_6506
Trichodesmium_erythraeum_IMS101
Oscillatoria_sp_PCC_10802
Lysobacter_enzymogenes
Leptolyngbya_sp_PCC_7375
Thalassospira_profundimaris
Thalassospira_xiamenensis
0.4

10

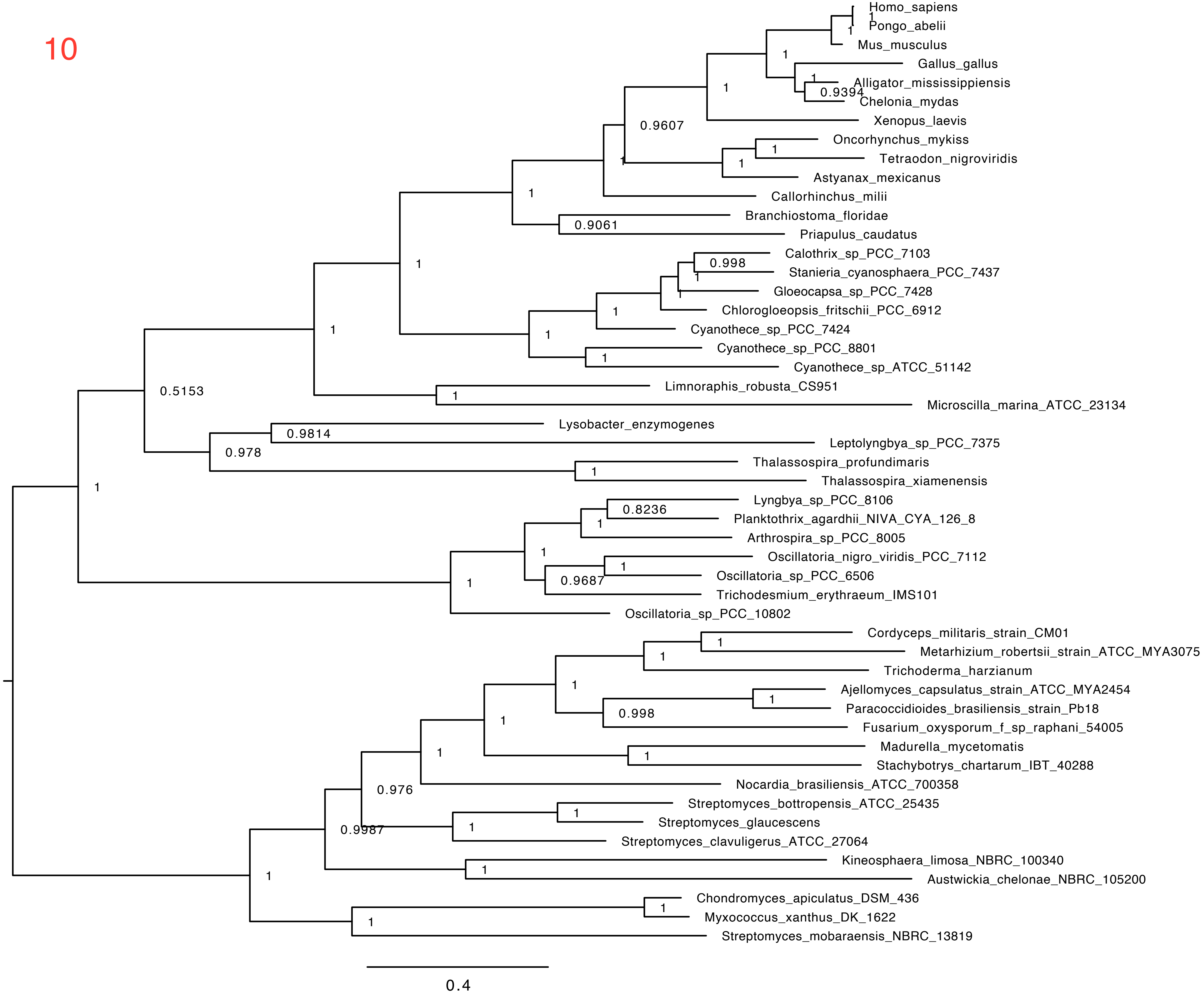
Homo_sapiens
Pongo_abelii
Mus_musculus
Gallus_gallus
Alligator_mississippiensis
Chelonia_mydas
Xenopus_laevis
Oncorhynchus_mykiss
Tetraodon_nigroviridis
Astyanax_mexicanus
Callorhinchus_milii
Branchiostoma_floridae
Priapulus_caudatus
Calothrix_sp_PCC_7103
Stanieria_cyanosphaera_PCC_7437
Gloeocapsa_sp_PCC_7428
Chlorogloeopsis_fritschii_PCC_6912
Cyanothece_sp_PCC_7424
Cyanothece_sp_PCC_8801
Cyanothece_sp_ATCC_51142
Limnoraphis_robusta_CS951
Microscilla_marina_ATCC_23134
Lysobacter_enzymogenes
Leptolyngbya_sp_PCC_7375
Thalassospira_profundimaris
Thalassospira_xiamenensis
Lyngbya_sp_PCC_8106
Planktothrix_agardhii_NIVA_CYA_126_8
Arthrospira_sp_PCC_8005
Oscillatoria_nigro_viridis_PCC_7112
Oscillatoria_sp_PCC_6506
Trichodesmium_erythraeum_IMS101
Oscillatoria_sp_PCC_10802
Cordyceps_militaris_strain_CM01
Metarhizium_robertsii_strain_ATCC_MYA3075
Trichoderma_harzianum
Ajellomyces_capsulatus_strain_ATCC_MYA2454
Paracoccidioides_brasiliensis_strain_Pb18
Fusarium_oxysporum_f_sp_raphani_54005
Madurella_mycetomatis
Stachybotrys_chartarum_IBT_40288
Nocardia_brasiliensis_ATCC_700358
Streptomyces_bottropensis_ATCC_25435
Streptomyces_glaucescens
Streptomyces_clavuligerus_ATCC_27064
Kineosphaera_limosa_NBRC_100340
Austwickia_chelonae_NBRC_105200
Chondromyces_apiculatus_DSM_436
Myxococcus_xanthus_DK_1622
Streptomyces_mobaraensis_NBRC_13819
0.9394
0.9607
0.9061
0.998
0.5153
0.9814
0.978
0.8236
0.9687
0.998
0.976
0.9987
0.4

11

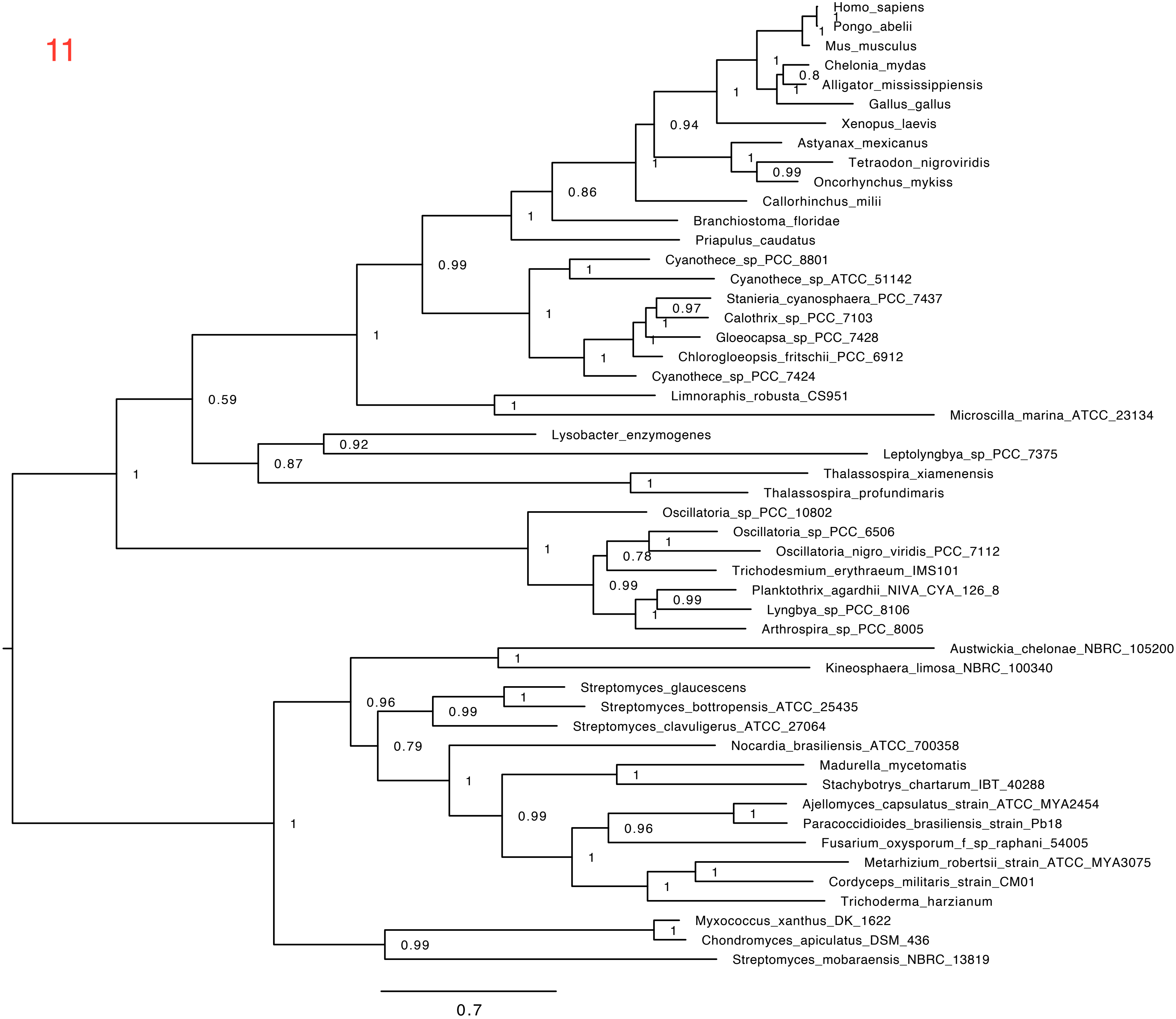
Homo_sapiens
Pongo_abelii
Mus_musculus
Chelonia_mydas
Alligator_mississippiensis
Gallus_gallus
Xenopus_laevis
Astyanax_mexicanus
Tetraodon_nigroviridis
Oncorhynchus_mykiss
Callorhinchus_milii
Branchiostoma_floridae
Priapulus_caudatus
Cyanothece_sp_PCC_8801
Cyanothece_sp_ATCC_51142
Stanieria_cyanosphaera_PCC_7437
Calothrix_sp_PCC_7103
Gloeocapsa_sp_PCC_7428
Chlorogloeopsis_fritschii_PCC_6912
Cyanothece_sp_PCC_7424
Limnoraphis_robusta_CS951
Microscilla_marina_ATCC_23134
Lysobacter_enzymogenes
Leptolyngbya_sp_PCC_7375
Thalassospira_xiamenensis
Thalassospira_profundimaris
Oscillatoria_sp_PCC_10802
Oscillatoria_sp_PCC_6506
Oscillatoria_nigro_viridis_PCC_7112
Trichodesmium_erythraeum_IMS101
Planktothrix_agardhii_NIVA_CYA_126_8
Lyngbya_sp_PCC_8106
Arthrospira_sp_PCC_8005
Austwickia_chelonae_NBRC_105200
Kineosphaera_limosa_NBRC_100340
Streptomyces_glaucescens
Streptomyces_bottropensis_ATCC_25435
Streptomyces_clavuligerus_ATCC_27064
Nocardia_brasiliensis_ATCC_700358
Madurella_mycetomatis
Stachybotrys_chartarum_IBT_40288
Ajellomyces_capsulatus_strain_ATCC_MYA2454
Paracoccidioides_brasiliensis_strain_Pb18
Fusarium_oxysporum_f_sp_raphani_54005
Metarhizium_robertsii_strain_ATCC_MYA3075
Cordyceps_militaris_strain_CM01
Trichoderma_harzianum
Myxococcus_xanthus_DK_1622
Chondromyces_apiculatus_DSM_436
Streptomyces_mobaraensis_NBRC_13819
0.7
