# Supplementary material for: Citrullination Was Introduced into Animals by Horizontal Gene Transfer from Cyanobacteria
Source: Mol Biol Evol. 2021 Nov 3;39(2):msab317. doi: 10.1093/molbev/msab317 (PMC8826395; doi:10.1093/molbev/msab317)
Supplement: msab317_Supplementary_Data [file msab317_supplementary_data.zip › Suppl_File_7_Topology testing.pdf]

Supplementary File 5

Tree	logL	deltaL	bp-RELL	Null rejected (–)	p-KH	Null rejected (–)	p-SH	Null rejected (–)	p-WKH	Null rejected (–)	p-WSH	Null rejected (–)	c-ELW	Null rejected (–)	p-AU	Null rejected (–)
1	-51668.34	1.14	0.013	-	0.440	+	0.921	+	0.439	+	0.958	+	0.042	+	0.466	+
2	-51667.2	0.00	0.169	+	0.502	+	1.000	+	0.502	+	0.930	+	0.174	+	0.583	+
3	-51668.34	1.14	0.025	-	0.439	+	0.921	+	0.439	+	0.960	+	0.041	-	0.465	+
4	-51678.85	11.65	0.092	+	0.231	+	0.550	+	0.185	+	0.416	+	0.088	+	0.163	+
5	-51667.2	0.00	0.174	+	0.498	+	0.948	+	0.498	+	0.947	+	0.173	+	0.584	+
6	-51667.9	0.71	0.232	+	0.467	+	0.904	+	0.467	+	0.901	+	0.207	+	0.569	+
7	-51668.34	1.14	0.037	+	0.439	+	0.921	+	0.440	+	0.990	+	0.041	+	0.466	+
8	-52073.61	406.41	0.000	-	0.000	-	0.000	-	0.000	-	0.000	-	0.000	-	0.000	-
9	-52071.33	404.13	0.000	-	0.000	-	0.000	-	0.000	-	0.000	-	0.000	-	0.000	-
10	-51668.34	1.14	0.045	+	0.440	+	0.921	+	0.439	+	0.979	+	0.042	+	0.466	+
11	-51669.1	1.91	0.213	+	0.427	+	0.867	+	0.427	+	0.855	+	0.192	+	0.480	+

Supplementary File 5: Topology Testing. Trees from Figure 1 were subjected to topology testing within IQTree under the model for tree 5, the best performing maximum likelihood tree. The table shows the deltaL, the ln(L) difference from the maximal ln(L) in the set; bp-RELL, the bootstrap proportion using the RELL method; p-KH, the p-value of the one sided Kishino-Hasegawa test; p-SH, the p-value of the Shimodaira-Hasegawa test; p-WKH, the p-value of the weighted KH test; p-WSH, the p-value of the weighted SH test; c-ELW, the Expected Likelihood Weight; and p-AU, the p-value of the approximately unbiased test. A minus sign denotes a tree that was rejected according to the topology test. Constraint trees (8 and 9), where eukaryotic sequences were constrained to be monophyletic, were rejected with a p value < 0.001 by all topology tests and are coloured in red.
